# Administration of 2-deoxy-D-glucose induces pyroptosis in murine breast cancer cells via cAMP/PKA/HK2 to impair tumor survival

**DOI:** 10.3389/fimmu.2025.1724476

**Published:** 2025-12-03

**Authors:** Tingting Pan, Shengqi Jin, Wei Gao, Kidong Eom, Jing Dong, Lin Li

**Affiliations:** 1 College of Animal Science and Veterinary Medicine, Shenyang Agricultural University, Shenyang, China; 2 Key Laboratory of Livestock Infectious Diseases, Ministry of Education, Shenyang Agricultural University, Shenyang, Liaoning, China; 3 The Fourth Affiliated Hospital of Heilongjiang University of Chinese Medicine, Harbin, Heilongjiang, China; 4 Department of Veterinary Diagnostic Imaging, College of Veterinary Medicine, Konkuk University, Seoul, Republic of Korea

**Keywords:** pyroptosis, 2-deoxy-D-glucose (2-DG), cAMP, PKA, HK2, breast cancer cells, mouse model

## Abstract

**Background:**

Breast cancer poses a severe threat owing to its high morbidity and mortality rates, which are largely attributed to drug resistance. Therefore, novel therapeutic targets need to be identified. Pyroptosis is an inflammatory cell death process mediated by gasdermin (GSDM) and dependent on caspases. Moreover, pyroptosis plays a role in regulating tumor progression and response to therapy. Furthermore, 2-deoxy-D-glucose (2-DG) is a glucose analogue that confers anticancer effects via metabolic interference. However, its mechanism of action in breast cancer remains unclear.

**Methods:**

To explore the effect of 2-DG on pyroptosis in EMT6/4T1 breast cancer cells, cell viability assays, immunoblotting, immunofluorescence, co-immunoprecipitation, and morphological analyses were performed. For *in vivo* studies, antitumor effects of 2-DG were assessed using xenograft models, and its safety was evaluated by monitoring body weight and conducting histological analysis.

**Results:**

2-DG induced cytotoxicity and pyroptosis in EMT6/4T1 breast cancer cells. Mechanistically, 2-DG activated cyclic adenosine monophosphate (cAMP)/protein kinase A (PKA) signalling, suppressed hexokinase 2 (HK2), and triggered caspase-3/GSDME-dependent pyroptosis. *In vivo* experiments demonstrated that 2-DG inhibited breast tumor growth without causing severe toxicity.

**Conclusions:**

These findings identified a novel metabolic-inflammatory axis (cAMP/PKA-HK2-caspase-3/GSDME) in breast cancer. Furthermore, study highlights the *in vivo* efficacy and safety of 2-DG and its ability to induce pyroptosis, thereby providing a basis for targeting drug resistance in breast cancer.

## Graphical Abstract

**Figure:**
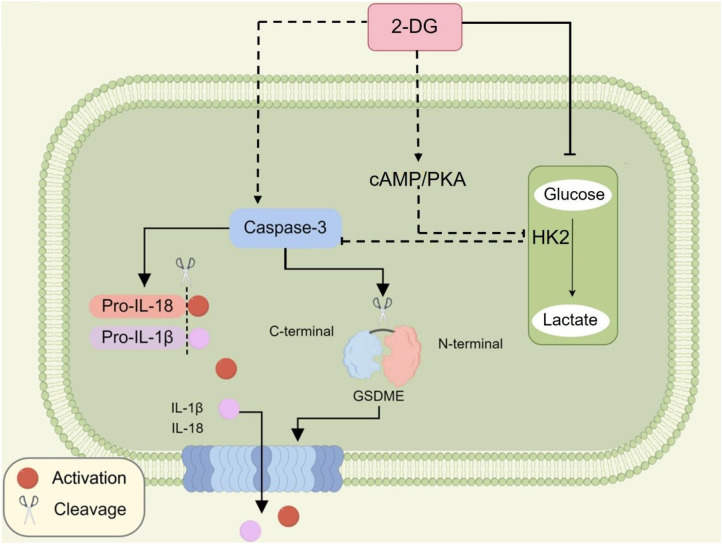

## Introduction

Breast cancer remains a formidable challenge, ranking as the most prevalent malignancy in women worldwide and imposing a heavy health burden (1). Despite substantial advancements in chemotherapy, targeted therapies, and immunotherapies, drug resistance and metastatic recurrence remain major barriers. Hence, long-term survival rates of patients with advanced-stage disease are low (2). Complexity of the disease, amplified by inter- and intra-tumor heterogeneity coupled with adaptive resistance mechanisms, frequently undermines therapeutic efficacy (3). Therefore, novel therapeutic agents are urgently needed to overcome these obstacles in breast cancer treatment.

Pyroptosis is an inflammatory programmed cell death driven by inflammasomes and mediated by gasdermin (GSDM) cleavage. This process is a pivotal modulator of tumor progression and a compelling target for cancer immunotherapy (4). GSDMs, including GSDMA to GSDME and non-death-inducing pejvakin, feature conserved N-terminal domains that oligomerize into membrane pores upon activation (5). GSDMD is activated via cleavage by inflammatory caspases (caspase-1/4/5 in humans and caspase-11 in mice), which relieves C-terminal autoinhibition (6). Conversely, GSDME is cleaved by caspase-3, which shifts cell death toward pyroptosis in expressing cells (6). Broader activation diversity has been revealed. Specifically, GSDMB activation by granzyme A, GSDMA by bacterial proteases, and non-mammalian GSDMA by caspase-1 (7, 8). These pores disrupt osmotic balance, thereby inducing lysis and releasing proinflammatory cytokines (interleukin [IL]- 1β, IL-18) and damage-associated molecular patterns. These amplify immune responses (e.g., recruiting cytotoxic T cells) and reinforce the role of pyroptosis in tumor immunosurveillance (6). However, against the backdrop of distinct metabolic reprogramming in breast cancer, such as increased glycolysis and subtype-specific metabolic traits, a critical knowledge gap remains regarding the mechanism by which these metabolic cues impinge on GSDM -mediated pyroptosis.

**Figure 1:**
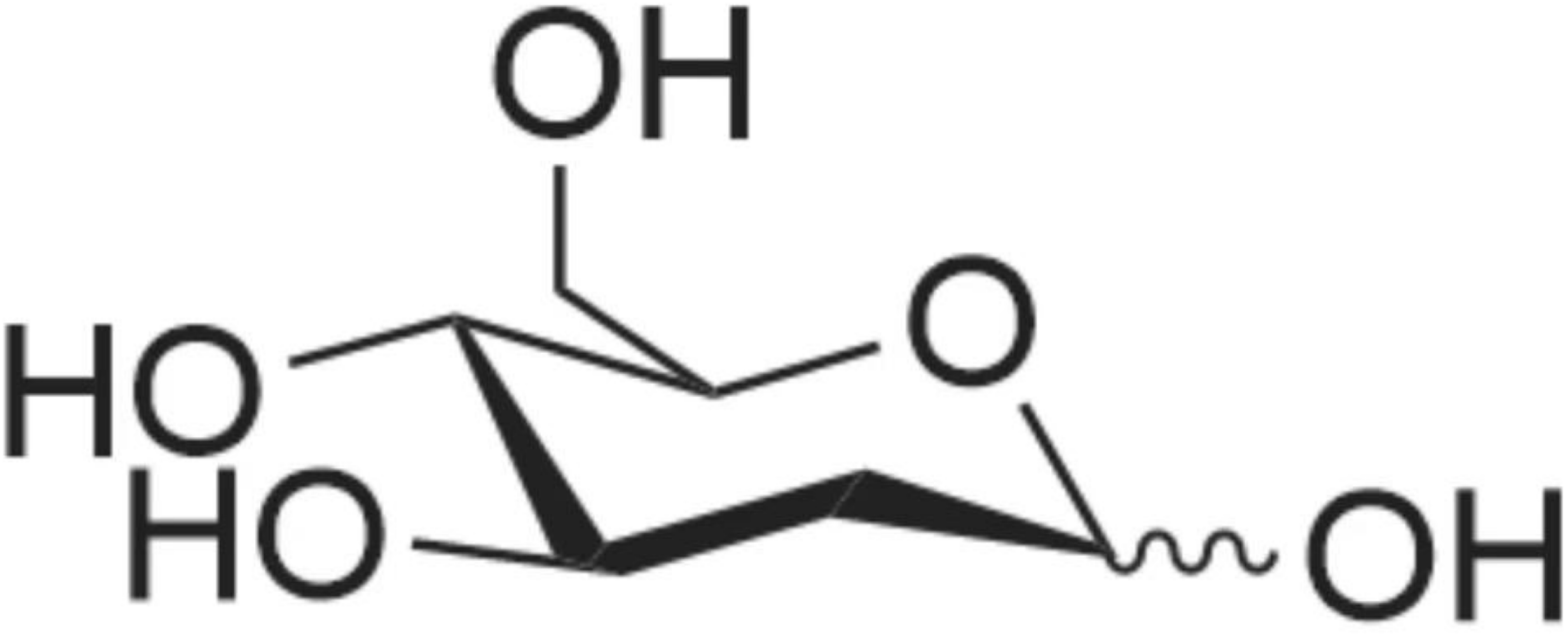
Chemical structure of 2-DG.

The glucose analogue 2- deoxy-D-glucose (2-DG, **Figure 1**) exerts antitumor effects by inhibiting hexokinase, the rate-limiting enzyme in glycolysis, thereby targeting cancer cells that rely on aerobic glycolysis (Warburg effect) for energy production and biomass synthesis (9). Furthermore, 2-DG suppresses the growth of lung, colon, and pancreatic cancer models via energy stress and autophagic cell death (10–12). However, role of 2-DG in regulating pyroptosis, especially in breast cancer, where metabolic traits (e.g., elevated glycolysis) (13) and subtype heterogeneity (e.g., higher hexokinase-2 (HK2) expression in triple-negative breast cancer) (13) may alter responses, remains unclear.

The cyclic adenosine monophosphate (cAMP)-protein kinase A (PKA) signalling axis operates as a master regulator of cellular energy balance. Extracellular stimuli fine-tune cAMP production to trigger PKA activation and the subsequent phosphorylation of diverse substrates and metabolic enzymes (14). Aberrant cAMP-PKA signalling accelerates breast cancer progression and fosters therapeutic resistance. Moreover, PKA governs glycolytic flow through the modulation of rate-limiting enzymes, such as HK2 (15). For instance, in hepatocellular carcinoma models, PKA activation diminishes HK2 expression, indicating a potential regulatory circuit that mediates the action of 2-DG in breast cancer (15). Interestingly, HK2 suppression has been linked to caspase activation, which suggests that 2-DG-induced HK2 downregulation may act as a molecular conduit linking metabolic stress and pyroptosis (16). However, in the context of breast cancer, it remains unclear whether 2-DG directly induces pyroptosis in breast cancer cells, whether cAMP-PKA signalling acts as a key driver of this process, or whether HK2 serves as a central mediator linking metabolic disturbances to inflammatory cell death.

This study investigated the specific mechanism by which metabolic cues regulate pyroptosis in mouse breast cancer by examining the glycolytic inhibitor 2-DG and its modulation of GSDM -driven inflammatory cell death in EMT6 and 4T1 models. Data showed that 2-DG engages in cAMP/PKA signalling to downregulate HK2, thereby initiating caspase-3-dependent GSDME-mediated pyroptosis. These findings reveal a novel crosstalk between glycolytic suppression and inflammatory cell death, thereby advancing our understanding of the metabolic control over cell death in breast cancer. Furthermore, this study highlights targetable nodes that support repurposing 2-DG or combinatorial regimens, including immunotherapies, to exploit pyroptosis and boost antitumor activity and outcomes.

## Methods

### Experimental model and study participant details

#### Cell lines

Procell Life Science & Technology Co., Ltd. (Wuhan, China) provided the mouse breast cancer cell lines EMT6 and 4T1, as well as the non-cancerous murine mammary epithelial cell line HC11. Every cell was maintained at 37 °C in a humidified atmosphere with 5% CO2 in a plastic culture plate or flask. EMT6 and 4T1 cells were cultivated in RPMI-1640 media supplemented with 10% heat-inactivated fetal bovine serum and 1% penicillin-streptomycin. Cells were subcultured every two to three days after reaching 80% confluence to prevent senescence.

#### Animal care, tumor model establishment, grouping, and drug administration

Female BALB/c mice, weighing 19 ± 1 g and aged 6–8 weeks, were acquired from Liaoning Changsheng Biotechnology Co., Ltd. Guide for the Care and Use of Laboratory Animals was followed in all animal research, which were authorized by Shenyang Agricultural University’s Institutional Animal Care and Use Committee. A 12-h light/dark cycle, autoclaved corn cob bedding that was changed every day, and a controlled temperature of 23 ± 1 °C were all features of the mice’s specified pathogen-free (SPF) housing. Throughout the trial, there was unlimited access to filtered water and sterilized mouse feed.

For tumor implantation, confluent 4T1 breast cancer cells were trypsinized, centrifuged at 1000 × g for 5 min, resuspended in PBS, and adjusted to 1.2×10^7^ cells/mL. Suspension was aliquoted into 1.5-mL tubes (1 mL each) and stored at 4 °C. Prior to inoculation, suspension was equilibrated to room temperature and gently resuspended with a 1-mL syringe. A 0.1-mL aliquot was subcutaneously injected into the left fourth mammary fat pad of each mouse. Tumor formation was confirmed 7 days post-inoculation by palpation, defining successful modeling.

Mice were grouped at random. Two main groups assessed 2-DG’s anti-tumor efficacy and safety: the anti-tumor efficacy group, where tumor-bearing mice with established xenografts were assigned to 3 subgroups (n=4/group): Model (MOD, daily oral PBS), 2-DG low-dose (2-DG-L, 300 mg/kg daily oral), and 2-DG high-dose (2-DG-H, 600 mg/kg daily oral) with 21 consecutive days of treatment; and the safety assessment group, where non-tumor-bearing mice were allocated to 2 subgroups (n=5/group): Control (CON, daily oral PBS) and 2-DG (1000 mg/kg daily oral) with 40 days of treatment. Euthanasia was performed via CO_2_ inhalation (flow rate: 50% chamber volume/min) followed by cervical dislocation to ensure humane endpoints.

### Method details

#### Preparation solutions

Compounds utilized for *in vitro* experiments were commercially sourced and detailed as follows. 2-DG (GLPBIO, USA, Cat# GC40110) was dissolved in sterile PBS (Yamei, China, Cat# P1010). Stock solutions of pharmacological inhibitorsgicalngomy Z-VAD-FMK (pan-caspase inhibitor; MedChemExpress, USA, Cat# HY-16658B), Z-DEVD-FMK (caspase-3-specific inhibitor; MedChemExpress, USA, Cat# HY-12466), and Necrostatin-1 (Nec-1, necroptosis inhibitor; MedChemExpress, USA, Cat# HY-15760)press prepared at 10 mmol/L in DMSO (Sigma-Aldrich) and further diluted in RPMI-1640 medium to working concentrations. The concentration used in this experiment was consistent with that described in Reference [34, 35]. Treatment conditions (dose/duration) were optimized based on preliminary experiments, with final DMSO concentrations maintained at <0.1% to preclude solvent-mediated toxicity [34].

#### Cell counting kit-8 assays

Cells were trypsinized, centrifuged at 300 × g for 5 minutes, and the pellet was resuspended in full media to a density of 7×10^4^ cells/mL for cell viability tests. Each well of a 96-well plate received a 100 μL aliquot of the cell suspension, which was then cultivated until 90–100% confluency was achieved. After that, cells were exposed to 2-DG for 24 h at titrated doses (0–20 mM). Each well received 10 μL of CCK-8 reagent (Solarbio, Beijing, China, Cat# CA1210) an hour before conclusion of the treatment period. At 450 nm, absorbance was measured. Cell viability (%) = (Absorbance of experimental group/Absorbance of solvent control group) × 100% was the formula used to calculate cell viability.

#### Colony formation assay

Cells were first plated on 6-well culture plates for the colony formation test. After the cultures reached about 80% confluence, they were subjected to various pharmacological agents as directed. Cells were fixed with 4% paraformaldehyde solution for 15 minutes at room temperature after specified incubation period. Following fixation, PBS was used to completely rinse each well before applying 0.1% crystal violet stain, which was made in 20% methanol. 30 minutes were spent staining, and any excess pigment was then washed off with distilled water. A Nikon Eclipse TS100 inverted phase-contrast microscope with a 10× objective lens was used to take digital pictures of the dyed cell monolayers.

#### Morphological observations

Logarithmic phase cells were plated in 6-well plates with three technical replicates per group for morphological evaluation. Cells were then grown for 24 to 48 h to allow adhesion and achieve 90hieve confluency. Each well was treated with two milliliters of compound-containing complete medium following two PBS rinses. After being incubated for 24 h at 37 °C with 5% CO_2_, the cells were gently rinsed once with PBS and immediately examined under a phase-contrast microscope (Nikon Eclipse TS100; 10× objective). To preserve data comparability, imaging parameters (such as exposure time and light intensity) were maintained constant across all experimental groups.

#### Western blot

Cells cultivated under certain conditions were gathered and lysed on ice for 30 minutes in RIPA buffer containing protease inhibitors (Beyotime, Shanghai, China) in preparation for Western blot analysis. Supernatants containing total proteins were obtained after lysates were centrifuged at 12,000 rpm for 20 minutes at 4 °C to pellet debris. BCA reagent (Beyotime, Shanghai, China) was used to measure the protein concentrations. SDS-PAGE was used to separate equal amounts of protein (30μg each sample), which were then transferred to PVDF membranes (Millipore, Massachusetts, USA). Following the transfer, membranes were blocked with rapid blocking solution for 20 minutes at room temperature. Then, they were incubated with the following primary antibodies for an entire night at 4 °C (**Table 1**): HK2 (1:1000, Cat No. 2867S, Cell Signaling Technology), Caspase-3 (1:1000, Cat No. 9662S, Cell Signaling Technology), GSDME (1:1000, Cat No. ab215191, Abcam), cAMP (1:2000, Cat No. A0151, ABclonal), PKA (1:1500, Cat No. 5106S, Cell Signaling Technology), IL-1β (1:1000, Cat No. 16806-1-AP, Proteintech), IL-18 (1:1200, Cat No. 20786-1-AP, Proteintech), and β-actin (1:50000, Cat No. 66009-1-Ig, Proteintech). Following primary incubation, membranes were washed and incubated with HRP-conjugated Goat Anti-Mouse IgG (H+L) (1:5000, Cat No. SA00001-1, Proteintech) and HRP-conjugated Goat Anti-Rabbit IgG (H+L) (1:5000, Cat No. SA00001-2, Proteintech) for 2 h at room temperature. Protein detection was performed using Super ECL Plus reagent (UElandy, Suzhou, China) and visualized with the Tanon chemiluminescent imaging system (Tanon, Shanghai, China).

**Table 1:** Key resources table.

Reagent or resource	Source	Identifier
Antibodies		
Caspase-1 rabbit polyclonal	ABclonal	Cat# A0964; Dilution: 1:1000
Caspase-3 rabbit polyclonal	ABclonal	Cat# A25309; Dilution: 1:1000
Caspase-8 rabbit polyclonal	ABclonal	Cat# A0251; Dilution: 1:1000
GSDMC rabbit polyclonal	ABclonal	Cat# A14550; Dilution: 1:1000
GSDMD rabbit polyclonal	ABclonal	Cat# A5526; Dilution: 1:1000
GSDME rabbit polyclonal	ABclonal	Cat# A25293; Dilution: 1:1000
IL-1β rabbit polyclonal	ABclonal	Cat# A16288; Dilution: 1:1000
IL-18 rabbit polyclonal	ABclonal	Cat# A23076; Dilution: 1:1000
CREB rabbit polyclonal	ABclonal	Cat# A13263; Dilution: 1:1000
p-CREB rabbit polyclonal	ABclonal	Cat# AP0647; Dilution: 1:1000
PKA rabbit polyclonal	ABclonal	Cat# A12446; Dilution: 1:1000
p-PKA rabbit polyclonal	ABclonal	Cat# AP0661; Dilution: 1:1000
HK2 rabbit polyclonal	ABclonal	Cat# A20829; Dilution: 1:1000
β-actin mouse monoclonal	ABclonal	Cat# AC004; Dilution: 1:2000
Goat Anti-Mouse IgG (H+L)	UpingBio	Cat# YP848536; Dilution: 1:5000
Goat Anti-Rabbit IgG (H+L)	ABclonal	Cat# RM70003; Dilution: 1:5000
siRNAs		
si GSDME-1	Sangon Biotech	Sense: 5’-GCAGCUUGUGGUACUGGAATT-3’ Antisense: 5’-UUCAUCAGCUGUCUGUUCCTT-3’
si GSDME-2	Sangon Biotech	Sense: 5’-GGAAGACACAGCUGAUGAATT-3’ Antisense: 5’-UUCUCCAGUACCACAAGCTT-3’
NC (Negative Control)	Sangon Biotech	5’-UUCUCCGAACGUGUCACGUTT-3’

#### Immunofluorescence

For immunofluorescence staining, cells were fixed in 4% paraformaldehyde (Biosharp, Cat# BL539A) in PBS (pH 7.4) at 37 °C for 60 min, rinsed three times with PBS, and permeabilized with 0.1% Triton X-100 (Beyotime, Cat# ST795) for 10 min at room temperature. Following blocking with 5% normal sheep serum (Solarbio, Cat# SL038) in PBST for 30 min, samples were incubated overnight at 4°C with primary antibody against GSDME (Abclonal, 1:100 dilution in blocking buffer). After washing, specimens were incubated with species-matched Cy3- or FITC-conjugated secondary antibodies (Abclonal, 1:100) for 1 h at 37 °C in the dark. Nuclei were counterstained with DAPI (Solarbio, Cat# C0065; 1 μg/mL) for 5 min prior to mounting with ProLong Diamond Antifade Mountant (Thermo Fisher, Cat# P36961). Fluorescence images were acquired using a Leica TCS SP8 confocal microscope with standardized parameters: DAPI (405 nm excitation/410–480 nm emission), FITC (488 nm excitation/500–550 nm emission), and Cy3 (552 nm excitation/560–620 nm emission). Identical laser power (15%), gain (600 V), and exposure time (2 μs) were maintained across all samples to ensure quantitative comparability.

#### Transfection

Cells were seeded in 6-well plates at 3×10^5^ cells/well in antibiotic-free complete medium until 50–60% confluence, then medium was replaced with 1.8 mL fresh antibiotic-free RPMI-1640. For transfection, 50 nM siRNA (targeting GSDME or NC) diluted in 125 μL Opti-MEM™ (Thermo Fisher, Cat# 31985070) was mixed with 5 μL Lipofectamine™ 3000 (Invitrogen, Cat# L3000015) diluted in 125 μL Opti-MEM™, incubated 15 min at room temperature to form complexes, and the 250 μL mixture was added dropwise to wells with gentle swirling; after 6 h incubation at 37 °C/5% CO_2_, medium was replaced with 2 mL complete medium (10% FBS + antibiotics), cells were harvested at 48 h post-transfection, with efficiency validated by Western blot (n=3).

#### Co-IP

For co-immunoprecipitation assays, cells were lysed in ice-cold RIPA buffer (50 mmol/L Tris-HCl, pH 7.4; 150 mmol/L NaCl; 1% Triton X-100; 0.1% SDS; 1 mmol/L EDTA; 1 mmol/L NaF; 1 mmol/L N_3_VO_4_) supplemented with 1× protease inhibitor cocktail (Roche, 4693159001). Lysates were vortexed intermittently over 30 min on ice, then centrifuged at 12,000 × g for 15 min at 4 °C to pellet debris. Following protein quantification via BCA assay (Pierce™, 23225), 500 μg of total protein was pre-cleared with Protein A/G Magnetic Beads (Biolinkedin, BLG-M020) for 1 h at 4 °C with gentle rotation. Pre-cleared lysates were incubated overnight at 4 °C with 2 μg of anti-HK2 (CST, 2867S) or anti-caspase-3 (CST, 9662S) antibodies under continuous rotation, followed by 2 h of bead capture. Immunocomplexes were washed 4 times with lysis buffer, eluted in 2× Laemmli buffer by heating at 95 °C for 5 min, and separated by 10% SDS-PAGE. Proteins were transferred to PVDF membranes (Millipore, IPVH00010), probed with primary antibodies (HK2: 1:1000; caspase-3: 1:1000) and HRP-conjugated secondary antibodies (CST, 7074S/7076S; 1:5000), then visualized using ECL Prime (Millipore, WBKLS0500) and quantified with ImageJ software (NIH).

#### Hematoxylin-eosin staining

Fresh tissue samples were promptly preserved in a 4% paraformaldehyde (PFA) solution and kept at 4 °t for histological examination. Dehydration, embedding, sectioning, and HE staining were among the histological procedures performed on the fixed tissues after they were transferred to Servicebio Technology Co., Ltd. (Wuhan, China). To provide a consistent morphological evaluation, all procedures were performed in accordance with accepted histology methods.

#### Immunohistochemical staining

Freshly harvested tissues were immediately fixed in 4% paraformaldehyde (PFA) and kept at 4°C for immunohistochemistry (IHC) examination. To target the proteins GSDME and HK2, fixed tissues were then transferred to Servicebio Technology Co., Ltd. (Wuhan, China) for IHC staining. Using tissue dehydration, paraffin embedding, sectioning, and antigen-antibody reaction, process adhered to the company’s normal practices. Strict quality control was applied to every sample’s processing to guarantee accurate and consistent staining findings.

#### Blood glucose, ALT, AST, creatinine, and BUN assay

Commercial ELISA kits were used to assess the levels of blood glucose, ALT, AST, creatinine, and BUN for biochemical analysis (Jianglai Biotech, China). To guarantee consistent detection and trustworthy findings, every test was carried out strictly in compliance with manufacturer’s instructions.

#### LDH release assay

For LDH release detection, cell, serum, and tumor tissue samples were processed as follows: Cell supernatants were collected post-treatment, centrifuged at 3,000 × g for 10 min at 4°C to remove debris, and stored at -80°C until analysis. Serum samples were obtained by centrifuging blood at 3,000 × g for 15 min at room temperature; separated serum was aliquoted and stored at -80°C. For tumor tissues, samples were weighed and homogenized in ice-cold lysis buffer (10 mM Tris-HCl, pH 7.4; 150 mM NaCl; 1% Triton X-100) using a tissue homogenizer. Homogenates were centrifuged at 12,000 × g for 15 min at 4°C, and supernatants were collected. LDH activity in all samples was measured using a commercial LDH Cytotoxicity Assay Kit (Jianglai Biotech, China) following the manufacturer’s instructions. Absorbance was read at 490 nm with a microplate reader (Molecular Devices, SpectraMax i3x), and LDH release was calculated relative to the kit-provided maximum LDH release control. All assays were performed in triplicate, with data expressed as mean ± standard deviation.

#### IL-18 and IL-1β assay

Commercial kits for the enzyme-linked immunosorbent test (ELISA) were used to detect IL-18 and IL-1β in cell, serum, and tumor tissue samples. After treatment, cell supernatants were gathered, centrifuged for 10 minutes at 4°C at 3,000 × g to remove debris, and then stored at -80°C until analysis. After centrifuging blood at 3,000 × g for 15 minutes at room temperature, the separated serum was aliquoted and kept at -80°C. Tumor tissue samples were weighed and homogenized using a tissue homogenizer in ice-cold PBS with 0.1% Triton X-100. Supernatants were then collected by centrifuging the samples at 12,000 × g for 15 minutes at 4°C. ELISA kits (Jianglai Biotech, China) were used to measure the levels of IL-18 and IL-1β in each sample in accordance with the manufacturer’s instructions. A microplate reader (Molecular Devices, SpectraMax i3x) was used to measure absorbance at 450 nm, and standard curves were used to quantify concentrations. Every test was carried out in triplicate, and the results were shown as mean ± standard deviation.

#### Quantification and statistical analysis

Graph Prism 8.0 and SPSS 22.0 software were used to analyze the data, which were then displayed as the mean ± standard deviation (SD) of three separate experiments. Unpaired t test was used to evaluate differences between two groups, and p values were computed using either a one-way or two-way analysis of variance (ANOVA). Statistical significance was indicated with ∗, ∗∗, ^, and ^^. (∗*P* < 0.01; ∗∗*P* < 0.01; ^*P* < 0.05; ^^*P* < 0.01). All statistical details of the experiments can be found in the figure legends.

## Results

### Cytotoxicity of 2-DG in EMT6 and 4T1 breast cancer cells

**Figure 2:**
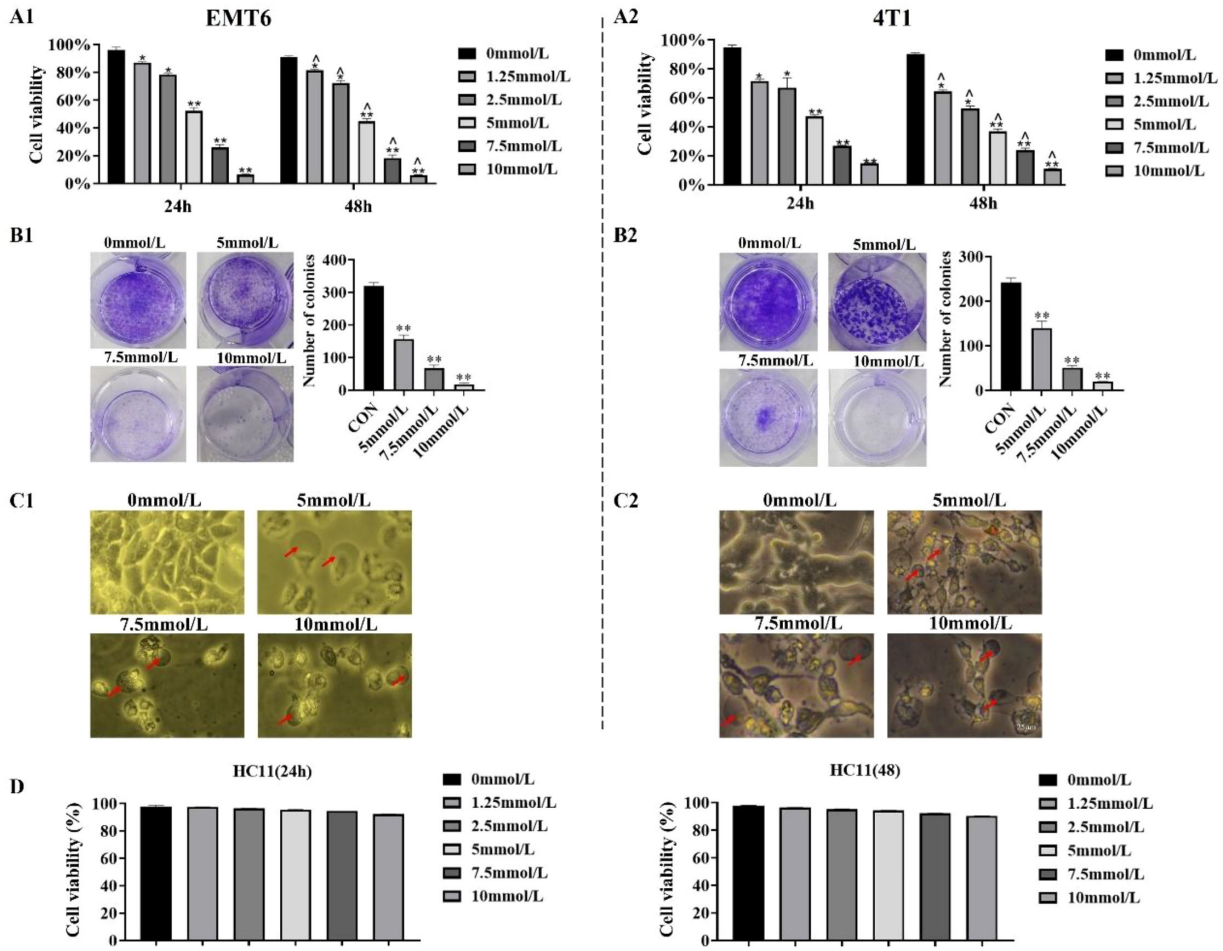
2-DG induces death of EMT6 and 4T1 cells. **(A1, A2)** CCK-8 assays showed dose-dependent cytotoxicity (n = 6). Data were mean ± SD *P < 0.05, **P < 0.01, ^*P* < 0.05, ^^*P* < 0.01. **(B1, B2)** Crystal violet staining revealed concentration-dependent reduction in clonogenicity (n = 3, **P < 0.01). **(C1, C2)** Light microscopy images displayed morphological changes, with red arrows indicating cellular swelling, membrane rupture, and gas bubble extrusion (n = 3). **(D)** CCK-8 assays showed no significant cell viability changes in HC11 cells across 2-DG concentrations at 24 h and 48 h (n = 6, Data were mean ± SD).

Cell counting kit 8 (CCK-8; **Figures 2A1, A2**) and crystal violet staining (**Figures 2B1, B2**) were used to explore 2-DG-driven toxicity in EMT6 and 4T1 cells to determine effects of 2-DG on cell proliferation and clonogenic capacity. 2-DG induced dose-dependent cytotoxicity across both lines, with CCK-8 assays defining a 24-h 50% inhibitory concentration of 5 mmol/L for proliferation impairment. Additionally, 2-DG suppressed colony formation in a concentration-dependent manner, thereby highlighting its ability to induce cell death. Morphological analysis (**Figures 2C1, C2**) revealed that 2-DG-treated cells displayed signature damage features, including cellular swelling, striking plasma membrane breakdown, and release of gas-like vesicles. Based on these morphological features, the study hypothesized that 2-DG induced pyroptosis or necroptosis in EMT6 and 4T1 cells. In contrast, CCK-8 assays on HC11 cells (**Figure 2D**) showed no significant changes in cell viability across different 2-DG concentrations at both 24h and 48h, indicating 2-DG exhibits selective toxicity towards EMT6 and 4T1 cancer cells without affecting normal HC11 cells.

### 2-DG induces pyroptosis in EMT6 and 4T1 breast cancer cells

**Figure 3:**
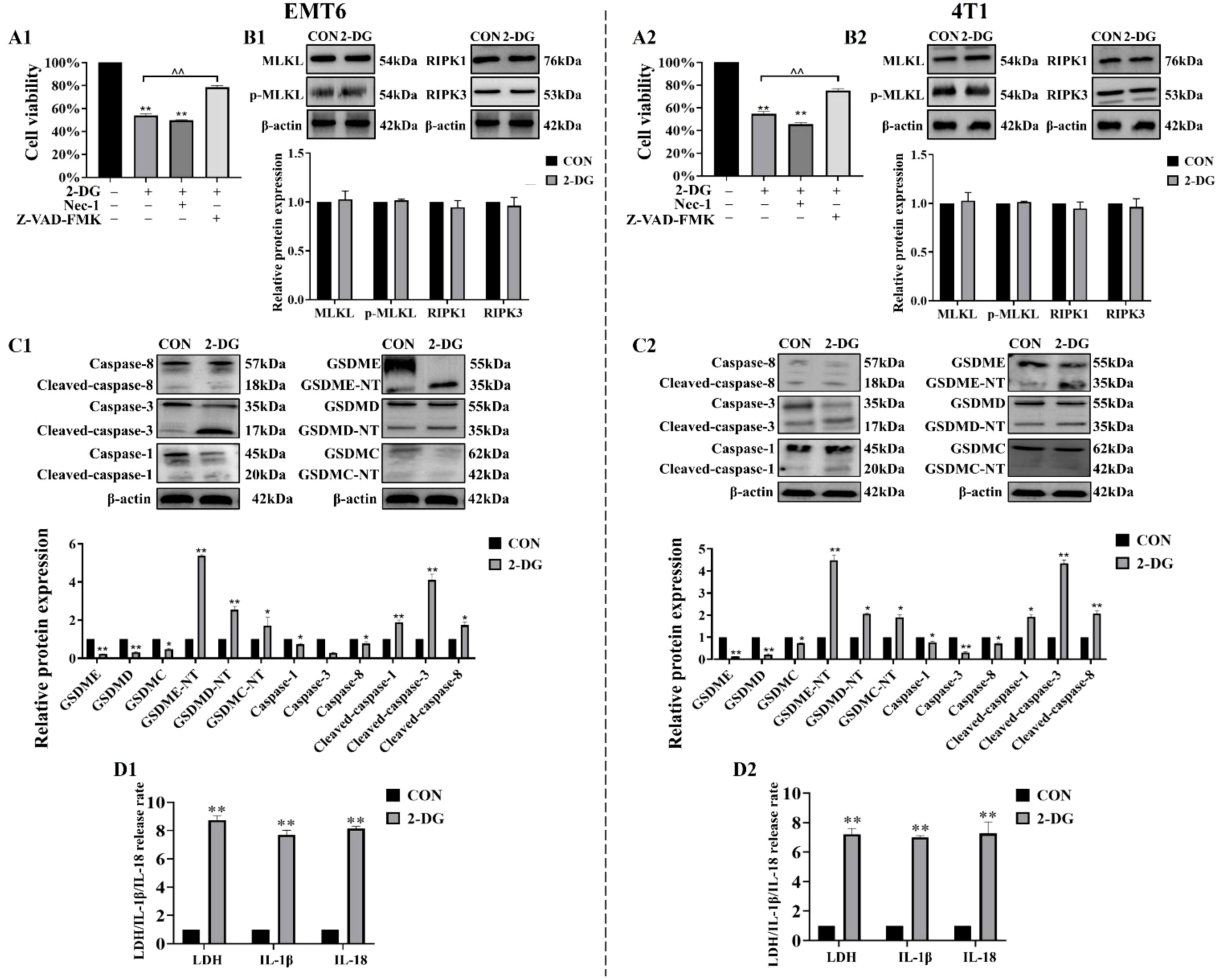
2-DG induces pyroptosis in EMT6 and 4T1 cells. **(A1, A2)** CCK-8 assays demonstrated that Z-VAD-FMK, but not Nec-1, abrogated 2-DG-induced cytotoxicity (n = 6, **P < 0.01). **(B1, B2)** Immunoblotting assays showed no significant upregulation of necroptosis markers (n = 3, *P* > 0.05). **(C1, C2)** Immunoblot analyses revealed activation of pyroptosis mediators (n = 3, **P < 0.01). **(D1, D2)** Quantification of LDH release and IL-1β/IL-18 secretion confirmed pyroptotic features (n = 3, **P < 0.01).

To elucidate form of cell death triggered by 2-DG, cells were pre-incubated with Nec-1 (necroptosis inhibitor) or Z-VAD-FMK (pan-caspase inhibitor) prior to 2-DG exposure. CCK8 assay revealed that Nec-1 failed to attenuate 2-DG-induced reduction in cell viability, whereas Z-VAD-FMK effectively mitigated 2-DG-elicited cell death. Immunoblotting confirmed that 2-DG potently upregulated activated pyroptosis effectors but did not induce expression of necroptosis-associated markers, such as phosphorylated mixed lineage kinase domain like protein and receptor-interacting protein kinase 1/3 (**Figures 3B1, B2**). These included GSDM family members (GSDMC/D/E) and executioner caspases (-1, -3, and -8), all of which were markedly activated (**Figures 3C1, C2**). Moreover, lactate dehydrogenase (LDH) release assays and IL-1β/IL-18 secretion quantification (**Figures 3D1, D2**) further confirmed pyroptosis occurrence. Taken together, these results demonstrated that 2-DG induces pyroptosis in EMT6 and 4T1 cells.

### GSDME mediates 2-DG-induced pyroptosis in EMT6 and 4T1 cells

**Figure 4:**
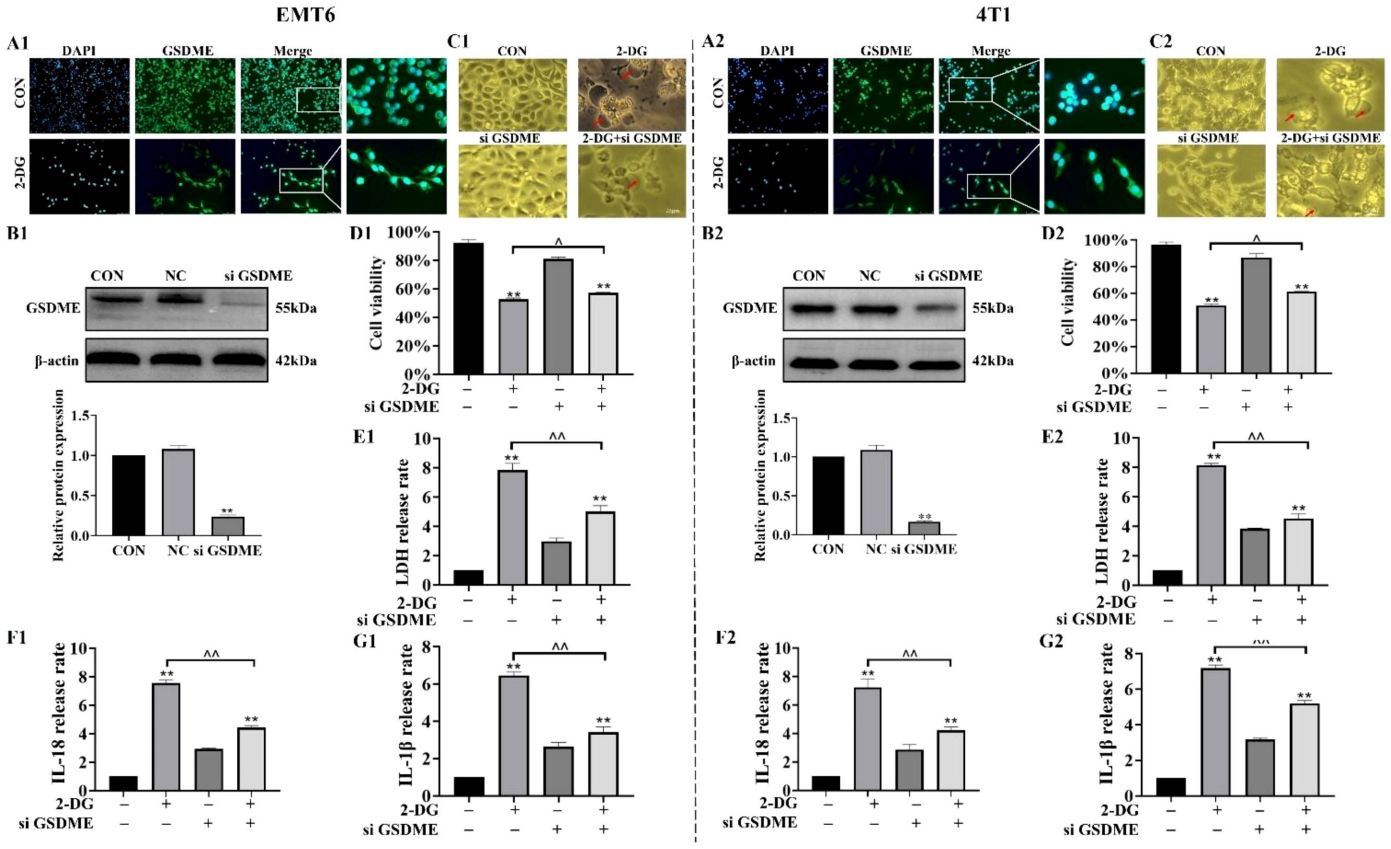
GSDME is the executor of 2-DG-induced pyroptosis. **(A1, A2)** Immunofluorescence staining (n = 3) showed diffuse GSDME localization in 2-DG-treated EMT6 and 4T1 cells. **(B1, B2)** Immunoblot assays (n = 3) confirmed effective GSDME silencing by si GSDME, **P < 0.01. **(C1, C2)** Light microscopy images (n = 3) revealed fewer pyroptotic cells in 2-DG + si GSDME groups compared to 2-DG alone. **(D1, D2-F1, F2)** Quantification assays (n = 3) showed reduced secretion of IL-1β, IL-18, and LDH in 2-DG + si GSDME groups versus 2-DG alone. Data were mean ± SD, **P < 0.01.

Immunoblotting (**Figures 3C1, C2**) revealed that 2-DG treatment reduced full-length GSDME levels while increasing generation of its cleaved, pore-forming GSDME-NT fragment in both EMT6 and 4T1 cells, thereby indicating GSDME activation. Immunofluorescence assays (**Figures 4B1, B2**) showed that 2-DG drives GSDME translocation from cytoplasm to cell membrane, which is consistent with its role in membrane pore formation during pyroptosis. To confirm role of GSDME in 2-DG-induced pyroptosis, GSDME was knocked down using GSDME-targeting small interfering RNA (siRNA) and non-targeting siRNA as controls (**Figures 4C1, C2**). Compared with 2-DG-treated wild-type cells, GSDME-deficient cells exhibited reduction in pyroptotic cells (**Figures 4D1, D2**) and significant decrease in release of LDH, IL-1β, and IL-18 (**Figures 4E1, E21, G1 G2**). Collectively, these findings demonstrated that GSDME is critical for mediating 2-DG-induced pyroptosis in murine breast cancer cells.

### Caspase-3 functions upstream of GSDME in 2-DG-induced pyroptosis

**Figure 5:**
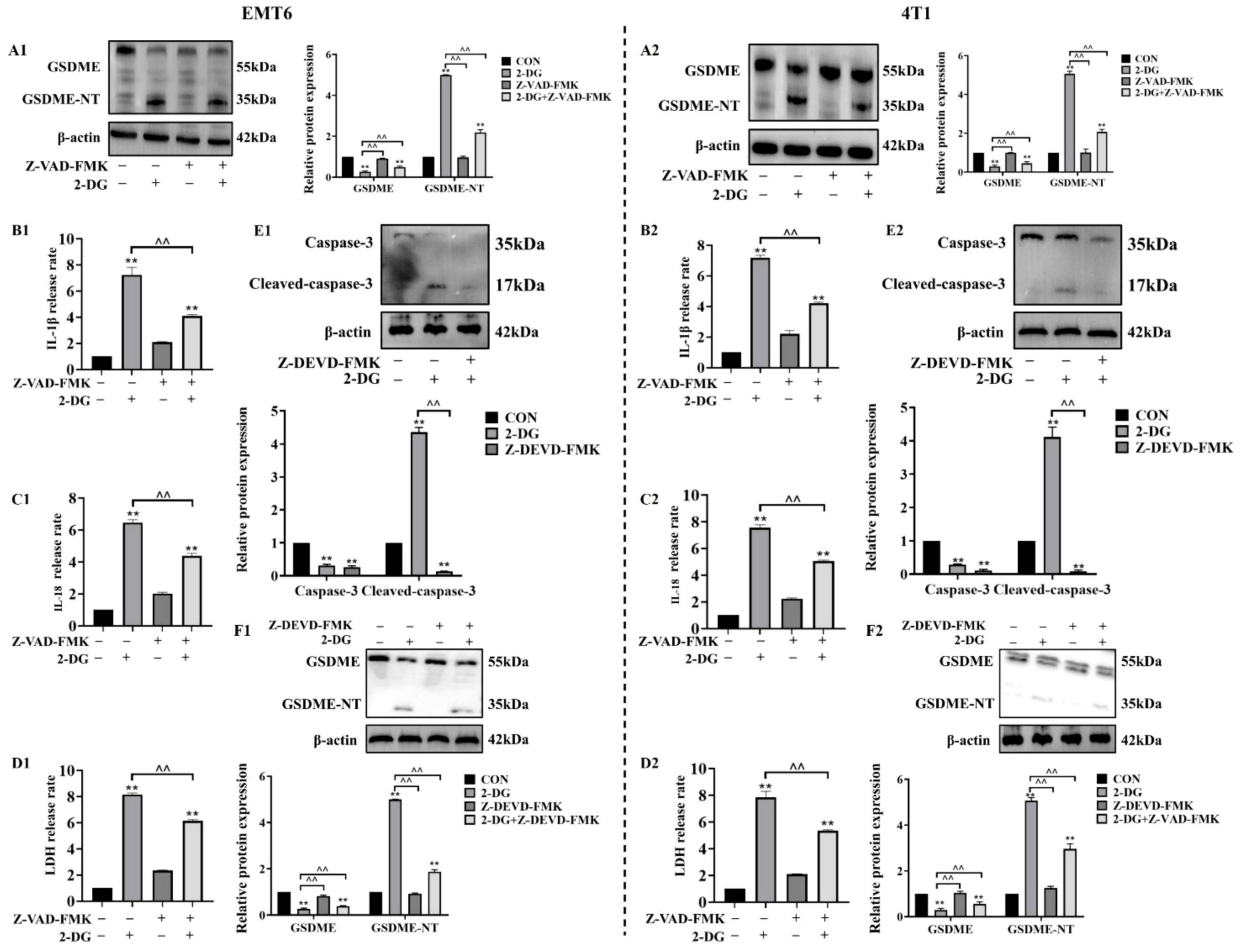
Caspase-3 mediates 2-DG-induced GSDME activation as an upstream factor. **(A1, A2)** Immunoblot analyses (n = 3) showed 2-DG induces GSDME cleavage in EMT6/4T1 cells, while Z-VAD-FMK (pan-caspase inhibitor) attenuates these changes. Quantification (normalized to β-actin): mean ± SD; **P < 0.01, ^^*P* < 0.01. **(B1, B2)** Quantification assays (n = 3) showed Z-VAD-FMK suppressed 2-DG-induced IL-1β release in both cell lines. Data were mean ± SD, **P < 0.01, ^^*P* < 0.01. **(C1, C2)** Quantification assays (n = 3) showed Z-VAD-FMK suppressed 2-DG-induced IL-18 release in both cell lines. Data were mean ± SD, **P < 0.01, ^^*P* < 0.01. **(D1, D2)** LDH release assays (n = 3) showed Z-VAD-FMK pretreatment reduced 2-DG-induced cytotoxicity in EMT6/4T1 cells. Data were mean ± SD, **P < 0.01, ^^*P* < 0.01. **(E1, E2)** Immunoblot analyses (n = 3) showed 2-DG upregulated cleaved caspase-3 (17 kDa, activated), while Z-DEVD-FMK (caspase-3 inhibitor) reduced this activation. Data were mean ± SD, **P < 0.01, ^^*P* < 0.01. **(F1, F2)** Immunoblot analyses (n = 3) showed Z-DEVD-FMK modulated 2-DG-mediated expression of full-length GSDME (55 kDa) and GSDME-NT (35 kDa). Data were mean ± SD, **P < 0.01, ^^*P* < 0.01.

To investigate whether caspase family members mediate 2-DG-induced and GSDME-dependent pyroptosis in EMT6 and 4T1 cells, cells were pretreated with pan-caspase inhibitor Z-VAD-FMK prior to 2-DG exposure. Thereafter, analyses of GSDME cleavage and release of LDH, IL-18, and IL-1β was conducted. These assessments revealed that Z-VAD-FMK effectively attenuated 2-DG-induced GSDME cleavage and reduced secretion of pyroptotic markers. Building on previous findings that chemotherapeutics activate GSDME through caspase-3, this study investigated functional role of caspase-3 in 2-DG-induced responses in EMT6 and 4T1 cells using Z-DEVD-FMK. Following pretreatment with Z-DEVD-FMK, cells were exposed to 2-DG, and protein levels of active caspase-3 and cleaved GSDME were measured. Z-DEVD-FMK considerably attenuated 2-DG-induced increases in activated forms of both caspase-3 and GSDME. Collectively, these findings confirm that 2-DG-induced pyroptosis via GSDME pathway is mediated by caspase family members, with caspase-3 functioning as critical upstream activator of GSDME (**Figure 5**).

### 2-DG activates the cAMP/PKA pathway to suppress HK2

**Figure 6:**
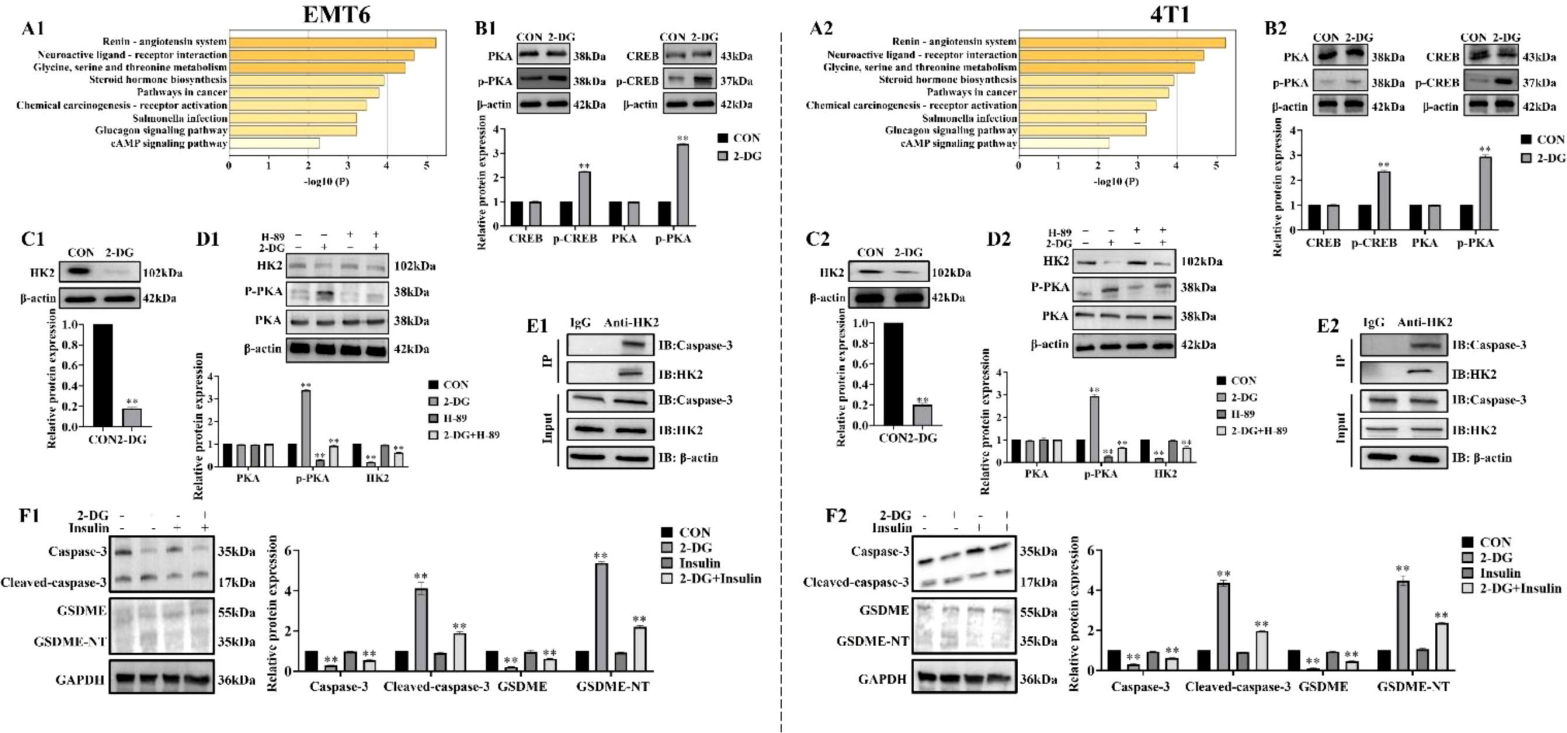
2-DG inhibits HK2 and activates the cAMP/PKA pathway to cause pyroptosis in EMT6 and 4T1 cells. **(A)** Top enriched pathways, such as the cAMP signaling system, were found by comparing genes linked to breast cancer (GeneCards) and 2-DG targets (SwissTargetPrediction), which were displayed as a bar plot of -log10(P-values). **(B)** Western blot analyses (n = 3) show that 2-DG upregulated phosphorylated CREB (p-CREB) and PKA (p-PKA), consistent with pathway activation. Quantification (normalized to β-actin) was presented as mean ± SD, **P < 0.01. **(C)** Western blot analyses (n = 3) demonstrating 2-DG downregulated HK2. Quantification (normalized to β-actin) was presented as mean ± SD, ** *P* < 0.01. **(D)** Western blot (n = 3) showing H-89 attenuated 2-DG-induced HK2 downregulation and PKA phosphorylation changes. Quantification (β-actin-normalized, mean ± SD, **P < 0.01). **(E)** Co-immunoprecipitation (Co-IP) tests (n = 3) verify that HK2 and Caspase-3 interact physically. Protein expression was confirmed by input controls, whereas IgG acted as a negative control. Pyroptosis signals may be modulated by this relationship. **(F)** Western blot experiments (n = 3) showing how 2-DG and Insulin affect the amounts of Caspase-3, Cleaved-caspase-3, GSDME, and GSDME-NT protein expression. An effective loading control was β-actin. Quantification of relative protein expression is shown in the bar graph. **P < 0.01. Insulin was used at a concentration of 100 nM to treat EMT6 and 4T1 cells for 48 h, aiming to activate HK2 expression.

2-DG targets were first identified using Swiss Target Prediction and breast cancer-associated genes were retrieved using Gene Cards. Integration of these datasets for Kyoto Encyclopedia of Genes and Genomes pathway analysis revealed that cAMP signalling pathway was co-enriched with top pathways, including renin-angiotensin system and neuroactive ligand-receptor interactions. This finding collectively suggests that 2-DG may activate cAMP/PKA cascades through metabolic perturbation. Notably, concurrent enrichment of cancer-related pathways supports the idea that cAMP/PKA activation likely drives tumor metabolic stress reprogramming, proliferation, and invasion (**Figures 6A1, A2**). To validate functional role of cAMP/PKA signalling in 2-DG-mediated tumor suppression, ability of 2-DG to activate this cascade was assessed by measuring phosphorylation of cAMP-responsive element-binding protein (p-CREB) and PKA (p-PKA). Western blot analysis (**Figures 6B1, B2**) showed that 2-DG treatment substantially increased phosphorylation of these targets, thereby confirming pathway activation. Given that 2-DG targets HK2, the study hypothesized that this interaction is mediated via cAMP/PKA axis. To test this hypothesis, cells were pretreated with PKA inhibitor H-89 prior to 2-DG exposure, and HK2 expression was subsequently evaluated. Western blot analyses (**Figures 6C1, C2–D1, D2**) demonstrated that H-89 mitigated 2-DG-induced HK2 downregulation, thereby confirming a PKA-dependent mechanism underlying 2-DG-mediated HK2 inhibition. Co-immunoprecipitation experiments (**Figures 6E1, E2**) verified that HK2 interacts with caspase-3 in an interaction that potentially regulates caspase-3 activation during pyroptosis. Moreover, western blot analyses (**Figures 6F1, F2**) showed that 2-DG treatment increased cleaved-caspase-3 and GSDME-NT, while reducing caspase-3 and full-length GSDME levels, and this effect was alleviated by Insulin. This indicated that activation of HK2 inhibits 2-DG-induced pyroptosis via caspase-3/GSDME pathway. Collectively, these findings demonstrated that 2-DG activates the cAMP/PKA pathway to suppress HK2 expression, thereby triggering caspase-3/GSDME-mediated pyroptosis in mouse breast cancer cells.

### Antitumor effects of 2-DG *in vivo*

**Figure 7:**
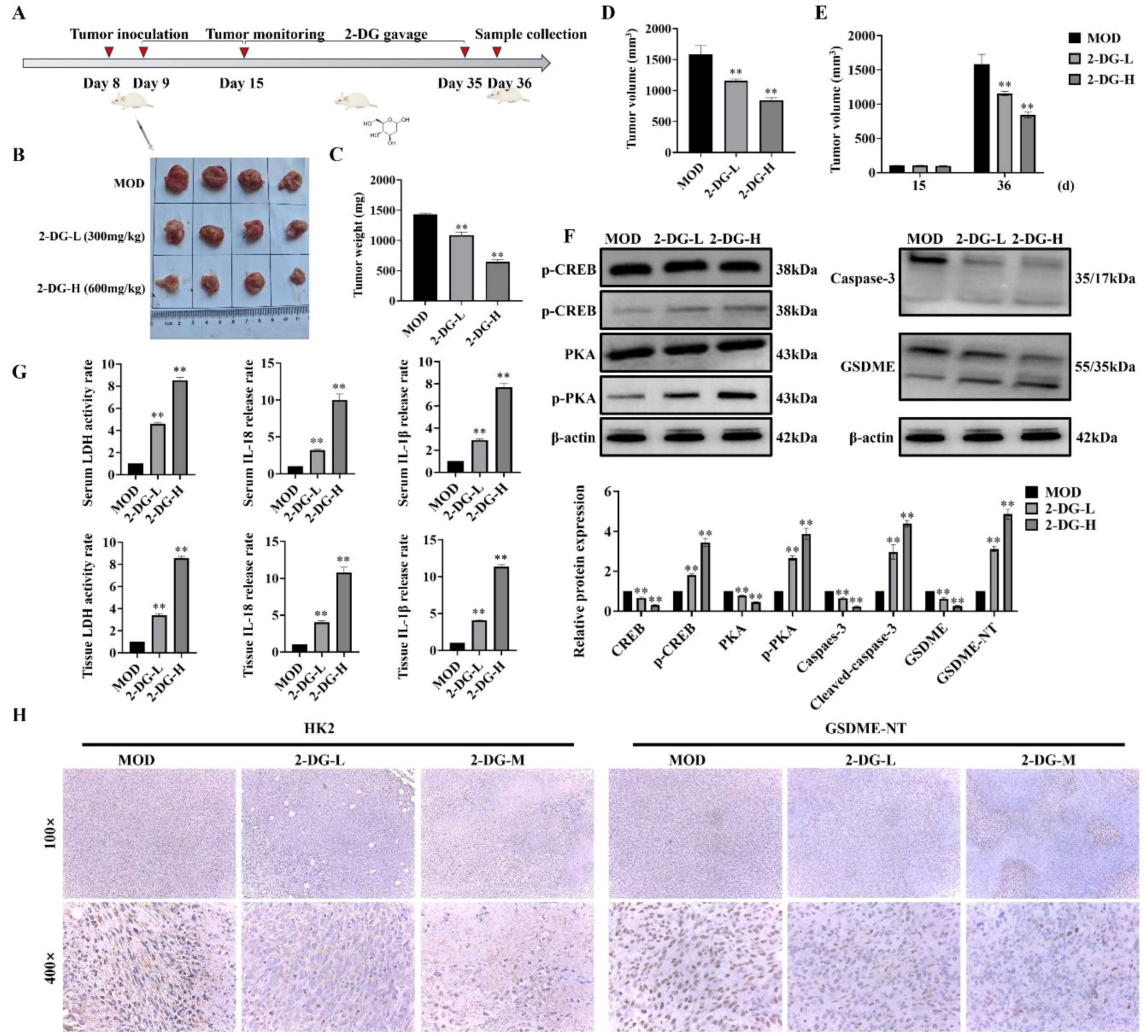
Antitumor effects of 2-DG *in vivo*. **(A)** Schematic timeline of the *in vivo* experiment: Murine breast cancer 4T1 cells were subcutaneously inoculated on Day 8; tumors were monitored regularly, followed by scheduled 2-DG gavage (2-DG-L: 300 mg/kg, 2-DG-H: 600 mg/kg, once daily); samples were collected on Day 36 (n = 4). **(B)** Representative tumor photographs of each group (MOD: model control; 2-DG-L: low-dose 2-DG; 2-DG-H: high-dose 2-DG). **(C)** Tumor weight statistics. Data: mean ± SD (n = 4, *P < 0.05, **P < 0.01). **(D)** Tumor volume statistics (calculated as V = 1/2 × length × width²). Data: mean ± SD (n = 4, significance markers as in **(C)**). **(E)** Tumor volume on Day 15 and Day 36 (calculation method as in D). Data: mean ± SD (n = 4, significance markers as in **(C)**). **(F)** Western blot analyses of cAMP/PKA pathway-related proteins (p-CREB, CREB, PKA, p-PKA) and pyroptosis-related proteins (Caspase-3, GSDME) in tumor tissues (β-actin as internal control) (n = 4). Data: mean ± SD, significance markers as in **(C)**). **(G)** LDH activity, IL-18, and IL-1β levels in serum and tumor samples (n = 4). Data: mean ± SD, significance markers as in **(C)**). **(H)** Immunohistochemical staining of HK2 and GSDME in tumor tissues (magnifications: 100× and 400×) (n = 4).

To investigate *in vivo* antitumor activity of 2-DG, a murine breast tumor model was established by subcutaneously injecting 4T1 murine breast cancer cells (**Figure 7A**). To evaluate antitumor efficacy of 2-DG, tumor weight and volume were measured after euthanasia, and tumor volume changes from day 15 to 36 were compared (**Figures 7B–E**). Both tumor weight and volume in 2-DG-treated groups were substantially reduced in a dose-dependent manner compared with that in model control, thereby indicating antitumor activity of 2-DG *in vivo*. Furthermore, ability of 2-DG to activate cAMP/PKA pathway in tumor tissues was explored by assessing expression of cAMP- and PKA-related proteins. Accordingly, 2-DG substantially upregulated protein levels of these pathway components, thereby confirming its activating effect. Concurrently, to determine if 2-DG induces pyroptosis *in vivo*, expression of caspase-3, GSDME, IL-18, and IL-1β was analysed in tumor tissues. 2-DG treatment pronouncedly activated these key pyroptosis-related factors, with more pronounced effects observed in high- (2-DG-H) and low-dose (2-DG-L) groups (**Figure 7F**). Additionally, release of LDH, IL-18, and IL-1β were detected in both serum and tumor samples (**Figure 7G**). This revealed that 2-DG markedly promoted secretion of all three markers. Finally, immunohistochemical analysis (**Figure 7H**) showed that HK2 protein was primarily localized in cytoplasm of tumor cells, and its expression was substantially downregulated following 2-DG treatment. In contrast, GSDME was distributed in both cytoplasm and cell membrane, with enhanced staining intensity and typical pyroptotic morphological features observed in 2-DG-treated groups. Together with statistical analysis of necrotic tumor areas, these findings indicated that 2-DG inhibited tumor growth by activating cAMP/PKA pathway, thereby suppressing HK2 expression and promoting GSDME-mediated pyroptosis.

### Safety assessment of 2-DG

**Figure 8:**
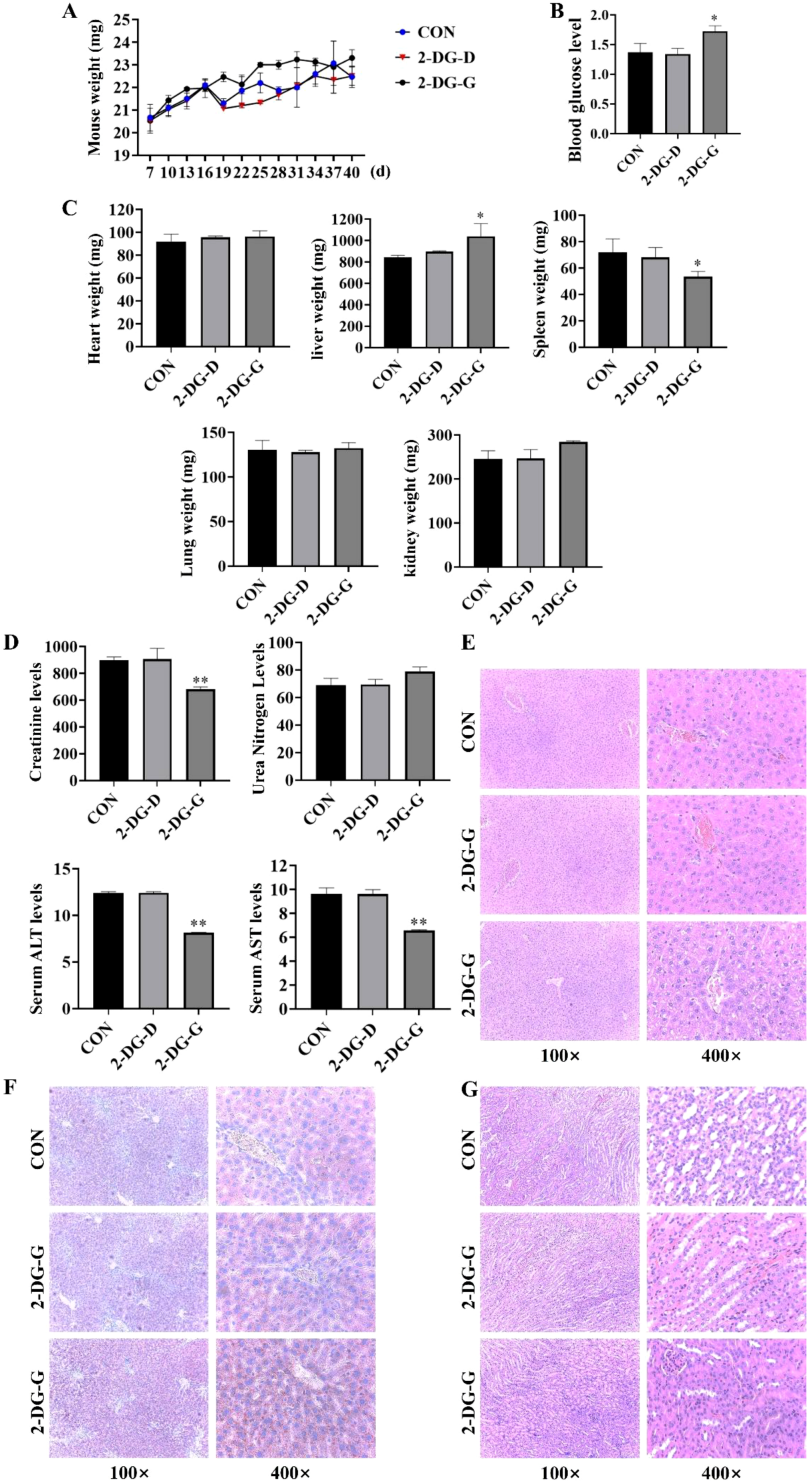
Safety evaluation of 2-DG in mice. **(A)** Body weight changes were monitored during treatment. Data: mean ± SD (n = 4), *P* > 0.05. **(B)** Blood glucose levels were measured. Data: mean ± SD (n = 4), *P* > 0.05. **(C)** Organ weight quantification of major organs (heart, liver, spleen, lung, kidney) showed no major alterations in most organs. Data: mean ± SD (n = 4), *P < 0.05. **(D)** Biochemical assays for serum ALT and AST creatinine urea levels. Data: mean ± SD (n = 4), **P < 0.01. **(E)** HE staining showed intact hepatic lobular architecture in the 2-DG-G group, but mild hepatocyte disarray was observed relative to CON (n = 4). **(F)** Oil Red O staining confirmed lipid droplet accumulation in 2-DG-G-treated livers (scattered pink droplets at 400× magnification), indicating disrupted lipid metabolism (n = 4). **(G)** HE staining showed mild pathological changes in kidneys of 2-DG-G-treated mice, including glomerular irregularities and tubular epithelial cell swelling (n = 4).

To evaluate safety profile of 2-DG, mice were respectively gavaged with 600 mg/kg 2-DG (2-DG-D) and 1000 mg/kg 2-DG (2-DG-G), and their body weights were monitored throughout treatment period (**Figure 8A**). 2-DG-D group showed no difference from control group, while 2-DG-G group exhibited marginally elevated blood glucose levels (**Figure 8B**). Following sacrifice, major organs were subjected to quantitative analyses (**Figure 8C**). Among these, only liver displayed notable alterations, appearing paler and exhibiting significantly increased weight in 2-DG-G-treated mice. Biochemical analyses (**Figure 8D**) revealed paradoxical changes in liver enzyme levels in 2-DG-G group: serum alanine aminotransferase (ALT) and aspartate aminotransferase (AST) levels were reduced. In kidneys, 2-DG-G treatment significantly decreased serum creatinine levels without affecting urea nitrogen levels. Histological assessments (**Figure 8E**) revealed that while hepatic lobular architecture remained intact in 2-DG-G-exposed livers, hepatocytes showed mild disarray and subtle intercellular space changes, with no evidence of necrosis or inflammation. Oil Red O staining confirmed lipid accumulation in 2-DG-G-treated livers, as indicated by scattered lipid droplets observed at 400× magnification, thus suggesting perturbed lipid metabolism (**Figure 8F**). HE staining of kidneys showed mild histological alterations, such as glomerular irregularities, tubular epithelial swelling, and minimal interstitial inflammation (**Figure 8G**). Collectively, these data indicate that 2-DG-D does not induce toxicity in mice, whereas 2-DG-G induces mild, subclinical perturbations in both liver and kidneys, without causing overt structural damage or severe functional impairment.

## Discussions

Breast tumors disrupt normal breast homeostasis and cause lethality through metastasis, thereby underscoring the need for targeted therapies (17). As a glucose analogue, 2-DG exerts its anticancer activity by disrupting tumor energy metabolism and triggering metabolic stress-mediated cell death (18). Furthermore, pyroptosis is an inflammatory programmed cell death modality that mediates antitumor effects through GSDM-dependent membrane rupture and immunogenic content release (19). In this study, cell viability assessment, pyroptotic effector profiling (expression, cleavage, and localization), and cAMP/PKA manipulation showed that 2-DG activated the cAMP/PKA axis to repress HK2, thereby initiating caspase-3/GSDME-dependent pyroptosis. This places 2-DG at intersection of metabolic targeting and inflammatory cell death, thereby providing preclinical groundwork to pursue pyroptosis-focused combination strategies for tackling treatment resistance in breast cancer.

The compound 2-DG is best known as a glycolysis inhibitor that exerts anticancer effects in diverse malignancies through context-specific mechanisms (20). In cancer-induced bone pain, nociception is alleviated by restricting microglial polarization toward the proinflammatory M1 phenotype and dampening neuroinflammation associated with tumor bone infiltration (21). In acute myeloblastic leukaemia ML-1 cells, 2-DG disrupts glycolytic flux via multi-targeted interference. This impairs hexokinase activity, reduces glucose uptake, and diminishes adenosine triphosphate production, thereby reshaping metabolic profiles and constraining cell expansion (22). This study extends this mechanistic landscape by defining a novel pathway in murine breast cancer cells, in which 2-DG elicits caspase-3/GSDME-dependent pyroptosis. This observation links 2-DG-driven metabolic disruption to inflammatory cell death, which is an outcome that differs from its well-characterized functions of triggering apoptosis or inducing metabolic stress. Together with prior observations, the study results underscore the multi-pronged therapeutic potential of 2-DG in oncology. This newly identified pyroptotic axis broadens the scope of 2-DG-based therapeutic strategies, thus creating a rationale for pairing metabolic targeting with immunotherapeutic interventions.

Traditional pyroptosis activation, such as the direct stimulation of the caspase-3/GSDME axis by chemotherapeutics, focuses on targeting apoptotic or inflammatory signalling (23). Examples include triptolide, which acts via mitochondrial pathways in head and neck cancer; osthole, piceatannol, and neobavaisoflavone, which engage in reactive oxygen species (ROS)-dependent cascades in liver cancer; and hypericin or triclabendazole, which induce pyroptosis through ROS buildup in breast cancer (24–27). These mechanisms tap into stress or inflammatory pathways, with minimal ties to metabolic processes. This study introduces a distinct metabolic-inflammatory crosstalk model, which is the first to link metabolic disruption of 2-DG to pyroptosis. Rather than directly activating caspases or triggering ROS, 2-DG operates through the cAMP/PKA/HK2 axis, which is a hub that converts metabolic stress into inflammatory cell death. This broadens pyroptosis regulation beyond direct signal activation. HK2 acts as a dual mediator; as a rate-limiting glycolytic enzyme, it sustains tumor metabolism, as a caspase-3 interactor, it controls the initiation of pyroptosis. Its inhibition via cAMP/PKA signalling connects metabolic stress from blocked glycolysis and disrupted energy balance to inflammatory death via caspase-3/GSDME activation. This addresses gaps in pyroptosis networks that overlook metabolic checkpoints in the shift to pyroptosis. Therefore, identifying HK2 as a node that integrates metabolic and inflammatory signals redefines pyroptosis triggers. Furthermore, this highlights metabolic perturbation as a viable strategy for activating inflammatory cell death and expanding regulatory mechanisms in the field.

Notably, the cAMP/PKA pathway exhibits a striking duality in cancer biology, with its role being highly context-dependent and shaped by factors, such as tumor type, cellular microenvironment, and intensity/duration of activation (28). The pathway can also function as a driver of pro-survival and growth-promoting programs in certain contexts, such as sustaining proliferation, enhancing therapeutic resistance, and facilitating metastatic dissemination (29). Study findings reveal a distinct context-specific role of this axis in 2-DG-induced pyroptosis. In contrast to these well-documented pro-tumorigenic functions, this study demonstrated that cAMP/PKA activation did not support tumor persistence. Instead, this activation triggers a switch toward pro-death signalling by inhibiting HK2, thereby initiating GSDME-dependent pyroptosis in EMT6 and 4T1 murine breast cancer cells. This highlights how the same pathway can exert antitumor effects under specific circumstances.

Consistent with reports of the antitumor activity of 2-DG via metabolic disruption and immune modulation, the *in vitro* findings were validated in a murine 4T1 breast cancer xenograft model, in which 2-DG reduced tumor volume and weight in a dose-dependent manner, similar to observations in hepatocellular carcinoma models (30). This was accompanied by upregulated cAMP/PKA components (p-PKA, p-CREB), pyroptosis markers (cleaved caspase-3, GSDME-N, IL-1β/IL-18), and HK2 downregulation, thereby confirming *in vivo* activation of the cAMP/PKA/HK2/GSDME pyroptotic pathway. These align with prior studies, in which cAMP/PKA signalling triggered inflammatory cell death via modulating caspase activation and GSDM cleavage, while elevated IL-1β/IL-18 mirrored pyroptosis-induced proinflammatory cytokine release in other tumors (31). Safety assessments revealed that high 2-DG doses induced mild hepatic lipid buildup and renal tubular alterations, which was consistent with its established metabolic effects (32). Notably, no severe organ toxicity was observed, which agrees with previous reports of transient disruptions, such as hyperglycemia, in rodent studies (33). This favorable profile, together with pyroptosis-driven immunogenicity via proinflammatory cytokines, supports the use of 2-DG as a metabolism-targeting agent for highly glycolytic breast cancer. Furthermore, the study findings rationalize the combination of 2-DG with immunotherapies to boost cytotoxicity and immune activation. This advance understanding of the mechanisms of 2-DG and provides a preclinical foundation for clinical investigation.

## Conclusions

This study revealed a novel mechanism underlying the antitumor effects of 2-DG in breast cancer. Specifically, activation of the cAMP/PKA pathway downregulates HK2, thereby triggering caspase-3/GSDME-dependent pyroptosis. Furthermore, *in vitro* validation revealed that this axis elucidates a unique metabolic-inflammatory crosstalk distinct from previously known 2-DG pathways. Precisely, the axis bridges glucose deprivation to immunogenic cell death via HK2. Given its capacity to induce pyroptosis and its favorable safety profile, 2-DG is a promising candidate for overcoming breast cancer resistance, with potential applications in combination immunotherapies. Although further validation in human models and clarification of cAMP/PKA-HK2 regulatory details are needed, this study deepens the understanding of metabolic checkpoints, expands the mechanistic scope of 2-DG, and provides preclinical groundwork for HK2-targeted pyroptosis-based strategies.

### Study limitations

Although the study findings are derived primarily from murine breast cancer cell lines (EMT6 and 4T1), their relevance to human breast cancer subtypes, particularly those with distinct genetic or metabolic profiles, requires further validation. Although *in vivo* xenograft models confirmed antitumor activity of 2-DG, long-term effects of 2-DG on tumor recurrence or metastasis, which are critical for clinical application, were not assessed. Finally, the mild metabolic perturbations observed in this study require more detailed analyses of systemic metabolic homeostasis to fully evaluate the safety of 2-DG in extended therapeutic interventions.

## Data Availability

The raw data supporting the conclusions of this article will be made available by the authors, without undue reservation.

## Glossary

2-DG: 2-Deoxy-D-glucose
BC: Breast cancer
cAMP: Cyclic adenosine monophosphate
PKA: Protein kinase A
HK2: Hexokinase 2
GSDME: Gasdermin E
GSDMC: Gasdermin C
GSDMD: Gasdermin D
IL-1β: Interleukin-1β
IL-18: Interleukin-18
LDH: Lactate dehydrogenase
CREB: cAMP response element-binding protein
siRNA: Small interfering RNA
NC: Negative control
WB: Western blot
IHC: Immunohistochemical
HE: Hematoxylin-eosin
CCK-8: Cell Counting Kit-8
Co-IP: Co-immunoprecipitation
DMSO: Dimethyl sulfoxide
PBS: Phosphate-buffered saline
FBS: Fetal bovine serum
ALT: Alanine transaminase
AST: Aspartate transaminase
BUN: Blood urea nitrogen
SPF: Specific pathogen-free
TNBC: Triple-negative breast cancer
DAMPs: Damage-associated molecular patterns
CTLs: Cytotoxic T lymphocytes
NK: Natural killer
PDE4D: Phosphodiesterase 4D
EP2: Prostaglandin E2 receptor 2
EMT: Epithelial-mesenchymal transition
PVDF: Polyvinylidene fluoride
BCA: Bicinchoninic acid
SDS-PAGE: Sodium dodecyl sulfate-polyacrylamide gel electrophoresis
TBST: Tris-buffered saline with Tween-20
ECL: Enhanced chemiluminescence
ANOVA: Analysis of variance
RPMI-1640: Roswell Park Memorial Institute 1640 medium
EDTA: Ethylene diamine tetraacetic acid
FITC: Fluorescein isothiocyanate
DAPI: 4’,6-Diamidino-2-phenylindole
OD: Optical density
IC_50_: Half-maximal inhibitory concentration
