# Supplementary figures and images for: Administration of 2-deoxy-D-glucose induces pyroptosis in murine breast cancer cells via cAMP/PKA/HK2 to impair tumor survival
Source: Front Immunol. 2025 Dec 3;16:1724476. doi: 10.3389/fimmu.2025.1724476 (PMC12708318; doi:10.3389/fimmu.2025.1724476)

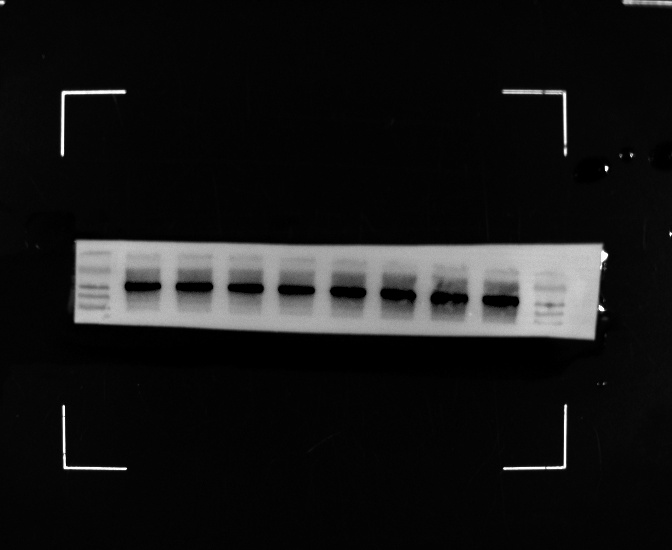

Supplement: Supplementary file 1 [file DataSheet1.zip › 4T1/actin.jpg]

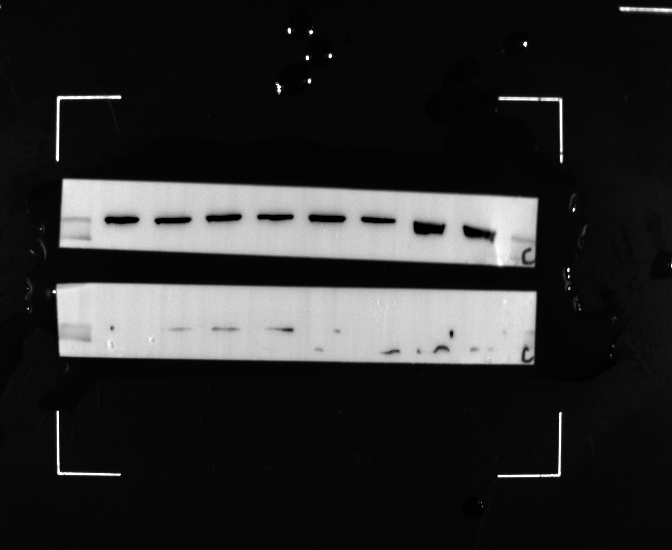

Supplement: Supplementary file 1 [file DataSheet1.zip › 4T1/CAS-8.jpg]

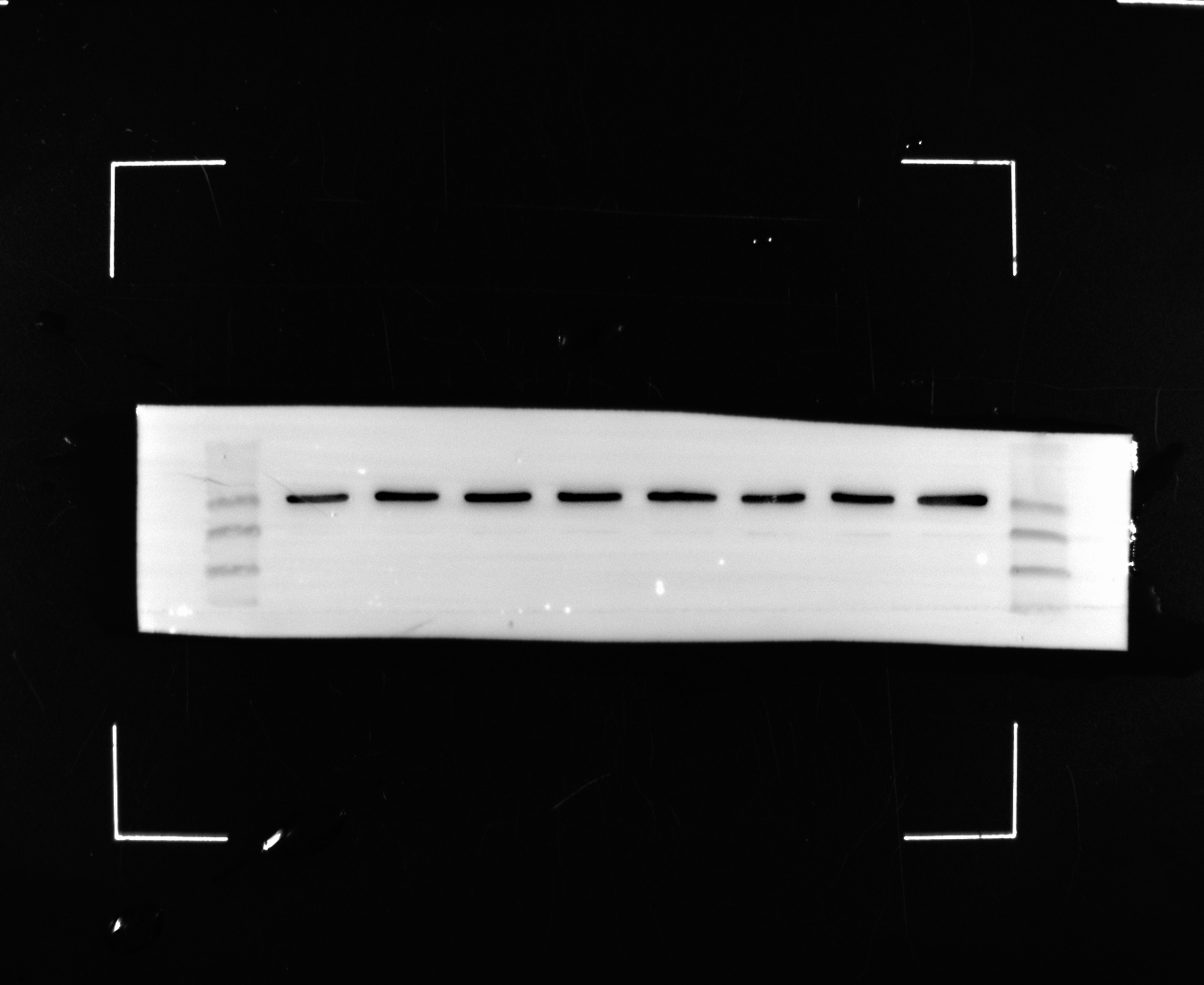

Supplement: Supplementary file 1 [file DataSheet1.zip › 4T1/MLKL.jpg]

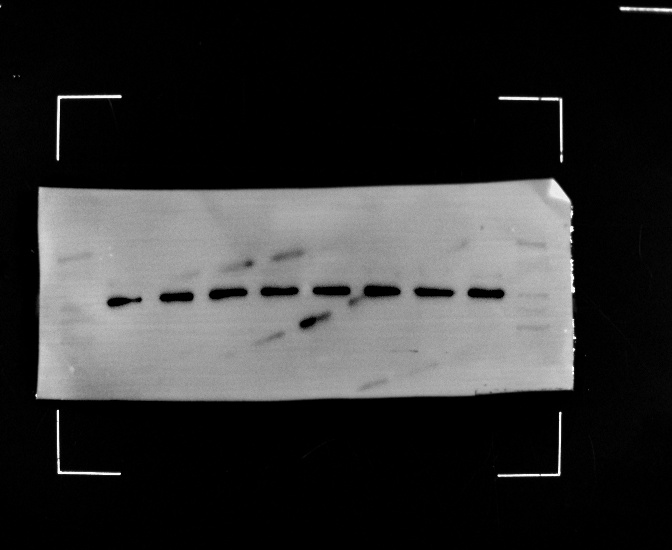

Supplement: Supplementary file 1 [file DataSheet1.zip › 4T1/RIPK1.jpg]

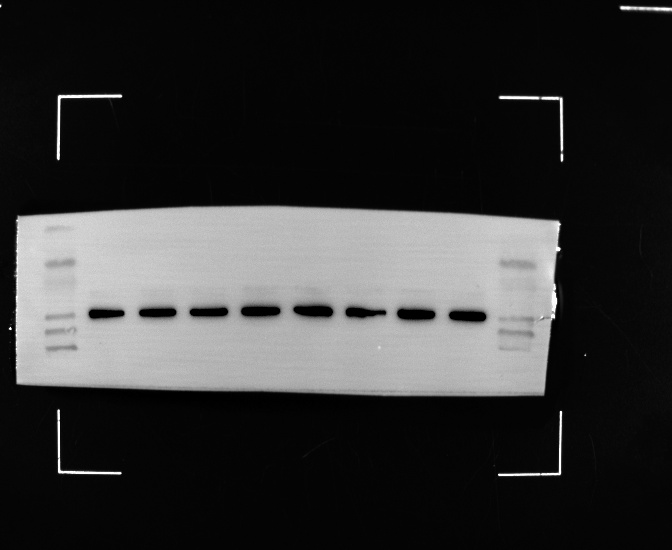

Supplement: Supplementary file 1 [file DataSheet1.zip › 4T1/RIPK3.jpg]

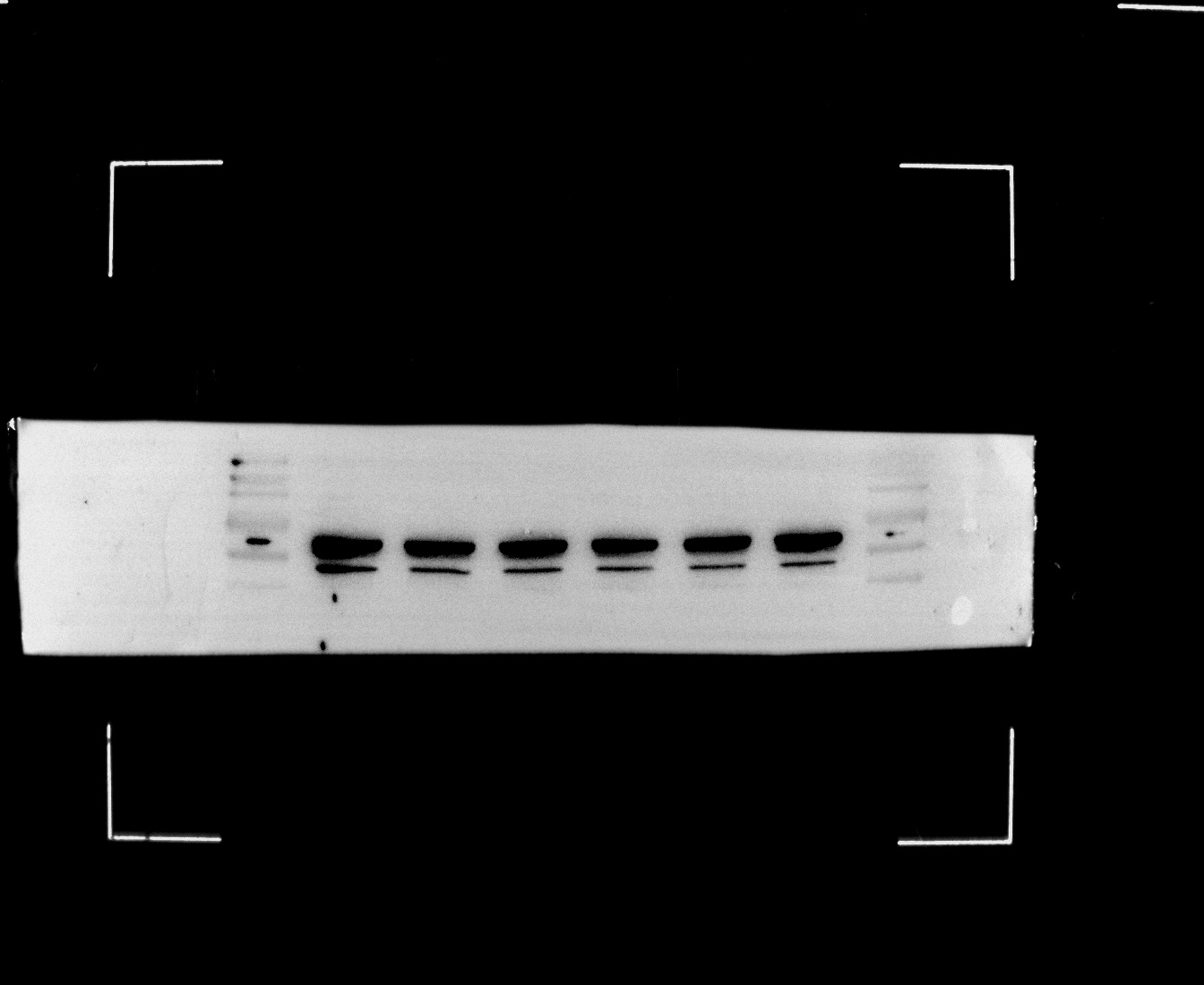

Supplement: Supplementary file 2 [file DataSheet2.zip › actin-4T1.jpg]

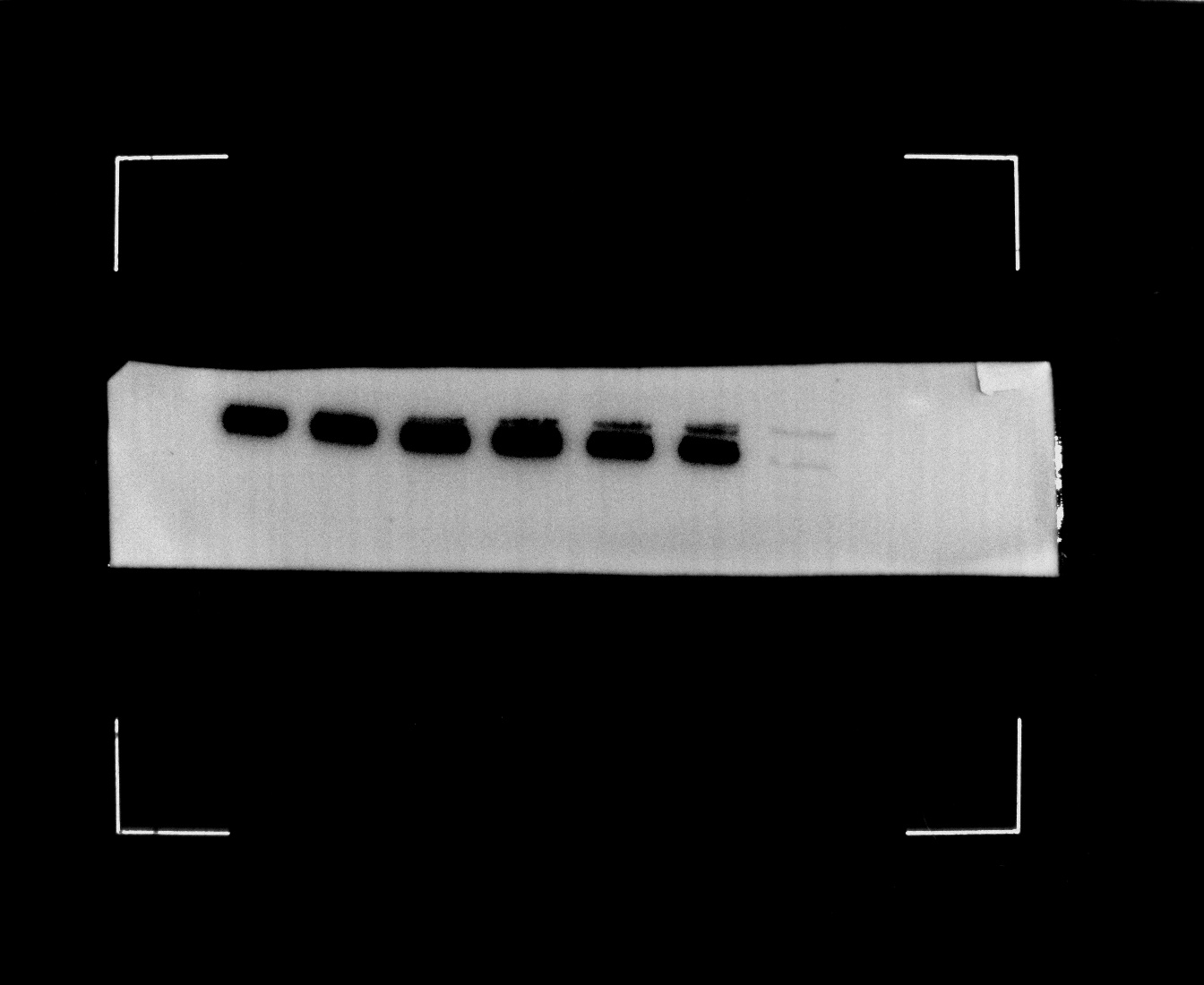

Supplement: Supplementary file 2 [file DataSheet2.zip › actin-EMT6.jpg]

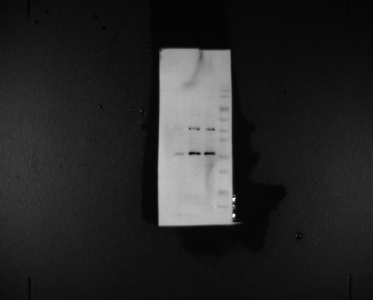

Supplement: Supplementary file 2 [file DataSheet2.zip › GSDME-4T1-1.jpg]

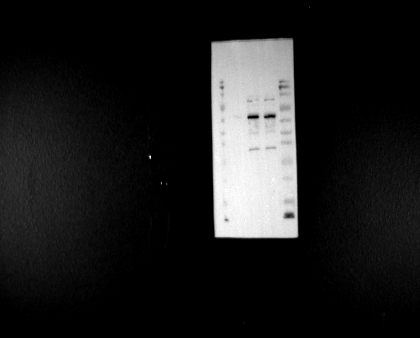

Supplement: Supplementary file 2 [file DataSheet2.zip › GSDME-4T1-2.jpg]

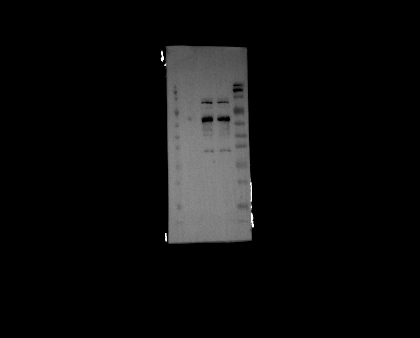

Supplement: Supplementary file 2 [file DataSheet2.zip › GSDME-4T1-3.jpg]

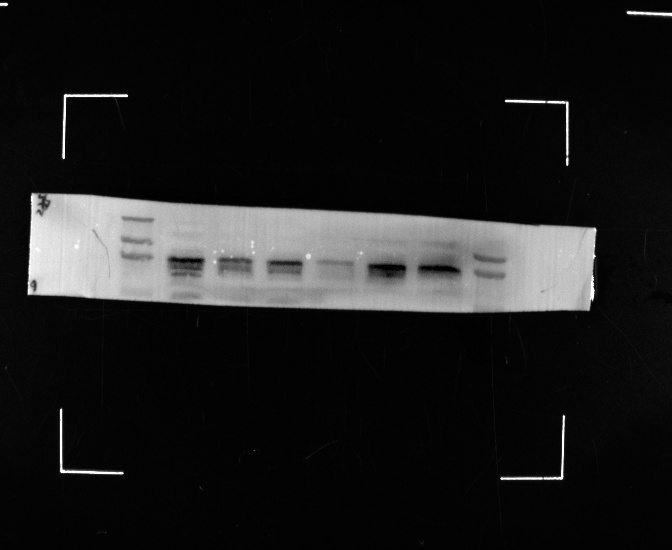

Supplement: Supplementary file 2 [file DataSheet2.zip › sicE-EMT6(2).jpg]

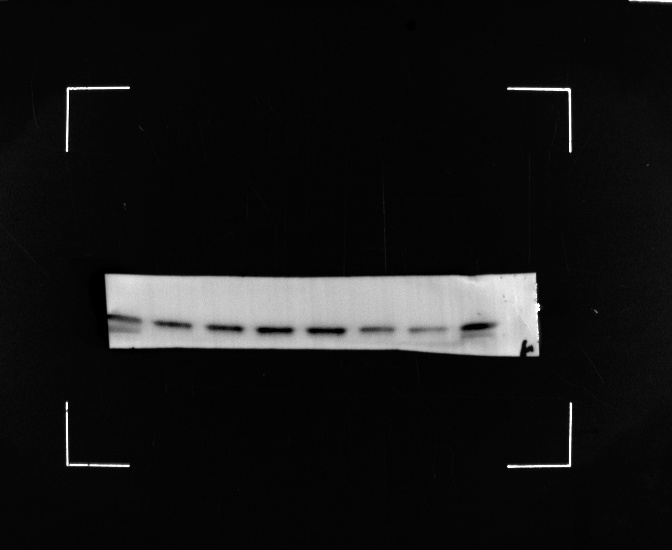

Supplement: Supplementary file 2 [file DataSheet2.zip › sicE-EMT6.jpg]

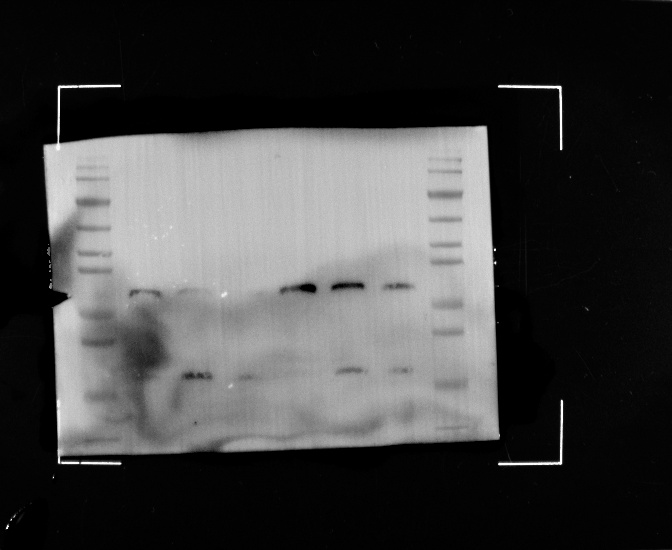

Supplement: Supplementary file 3 [file DataSheet3.zip › CAS-3-4T1-1.jpg]

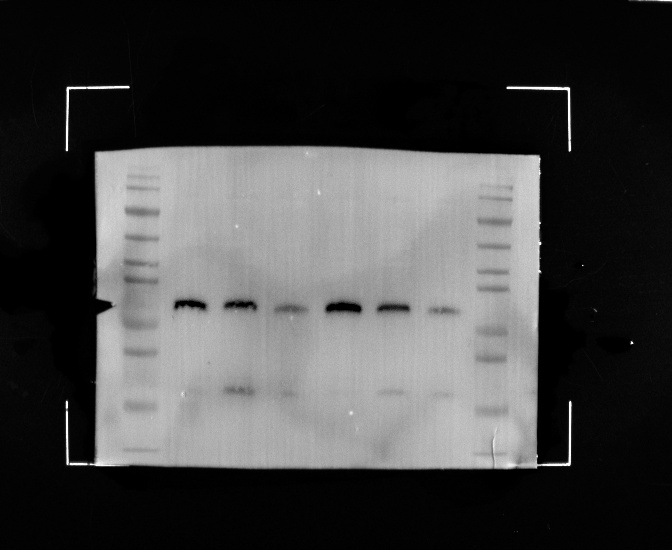

Supplement: Supplementary file 3 [file DataSheet3.zip › CAS-3-EMT6-1.jpg]

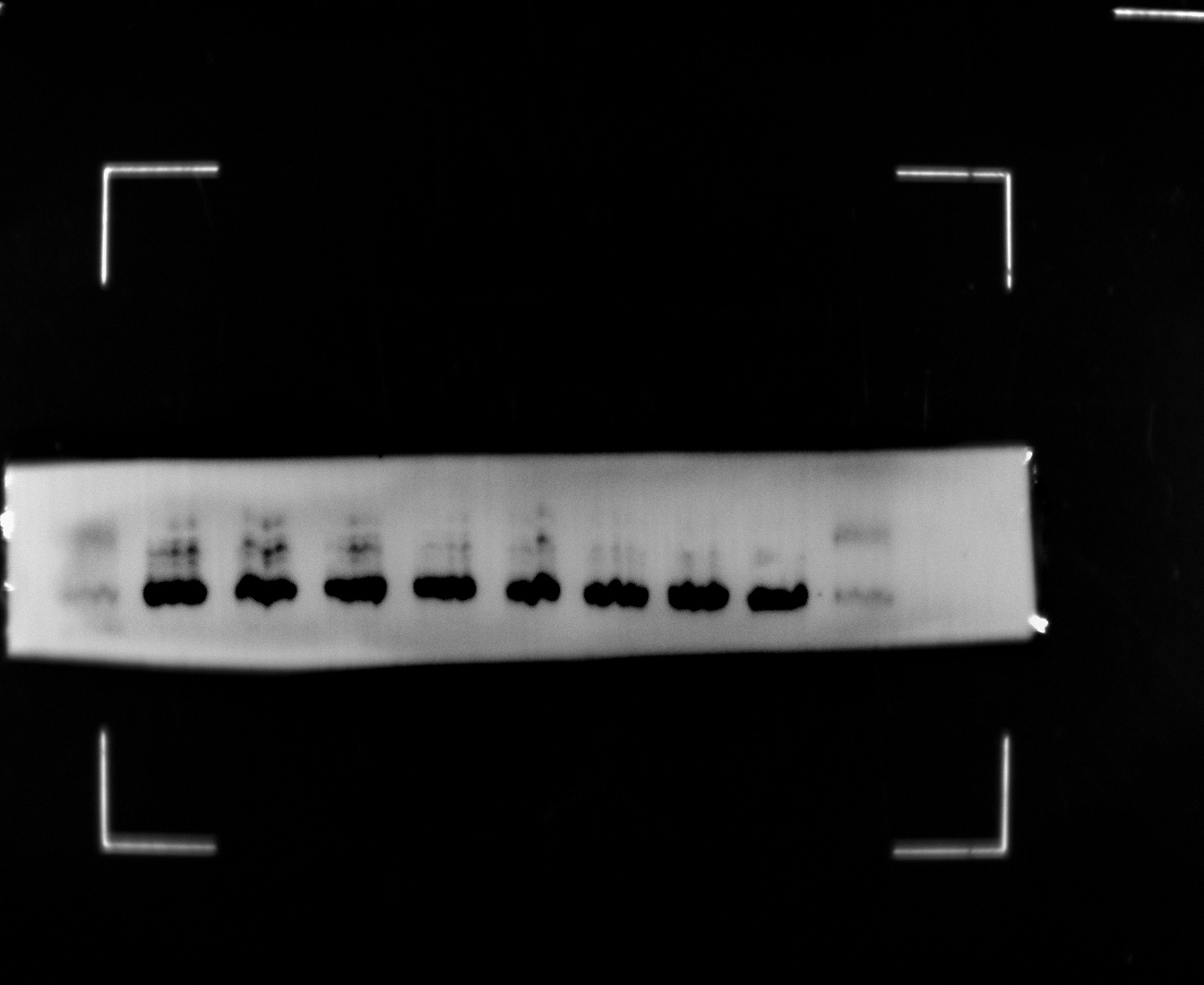

Supplement: Supplementary file 3 [file DataSheet3.zip › GAPDH-4T1.jpg]

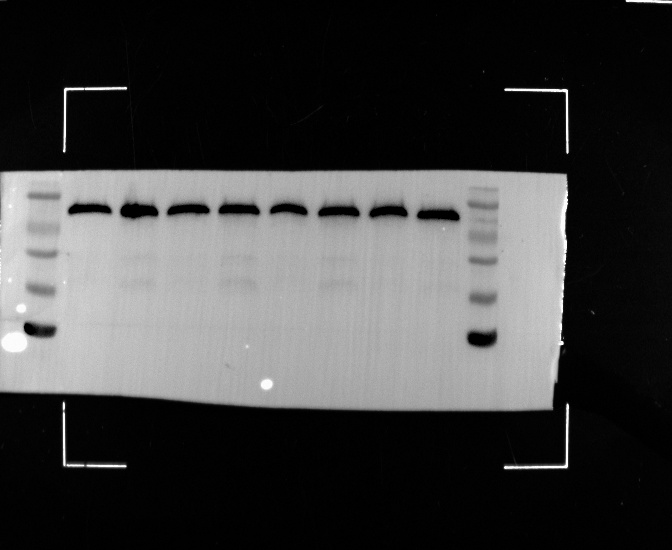

Supplement: Supplementary file 3 [file DataSheet3.zip › GAPDH-EMT6.jpg]

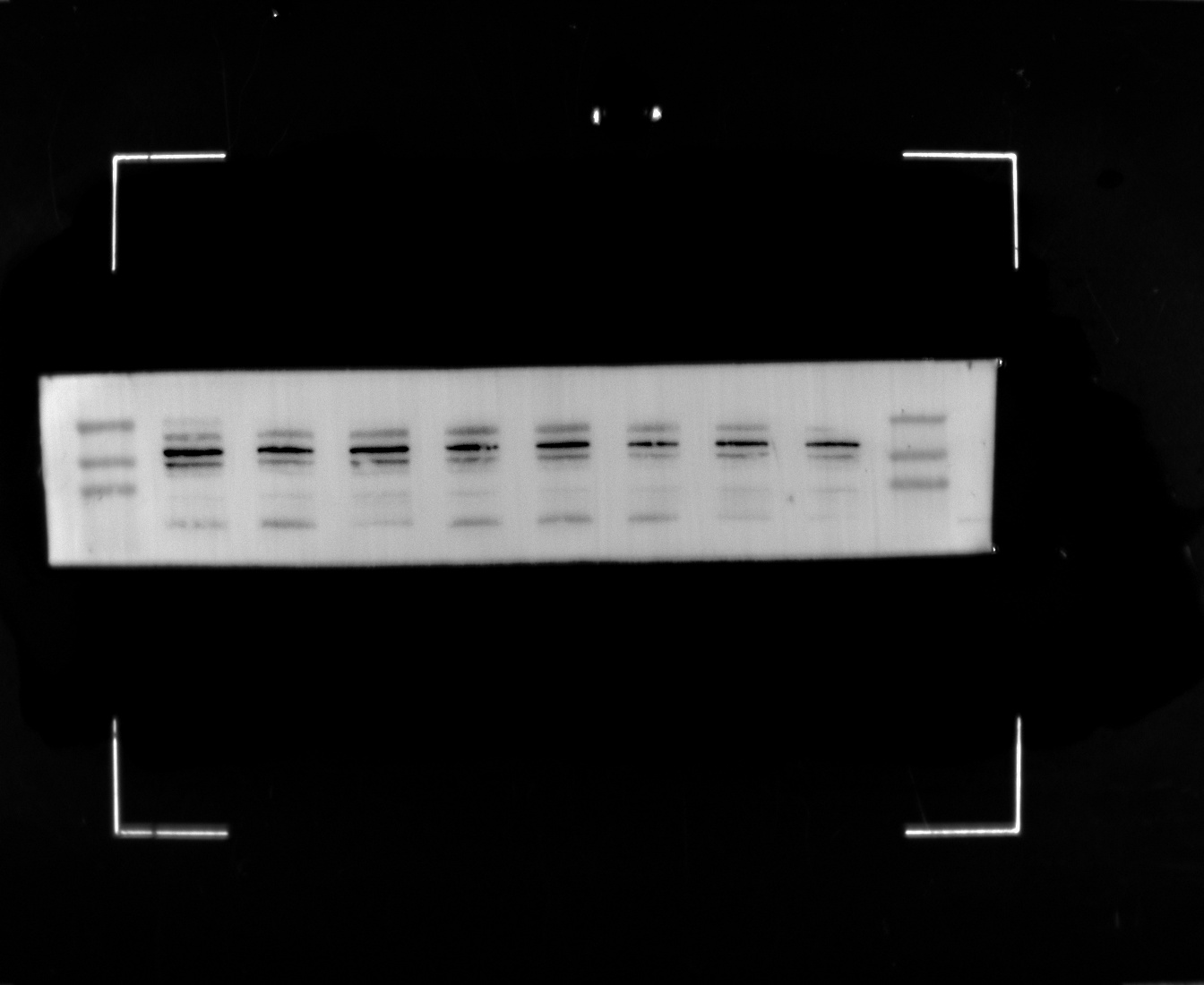

Supplement: Supplementary file 3 [file DataSheet3.zip › GSDME-4T1-1.jpg]

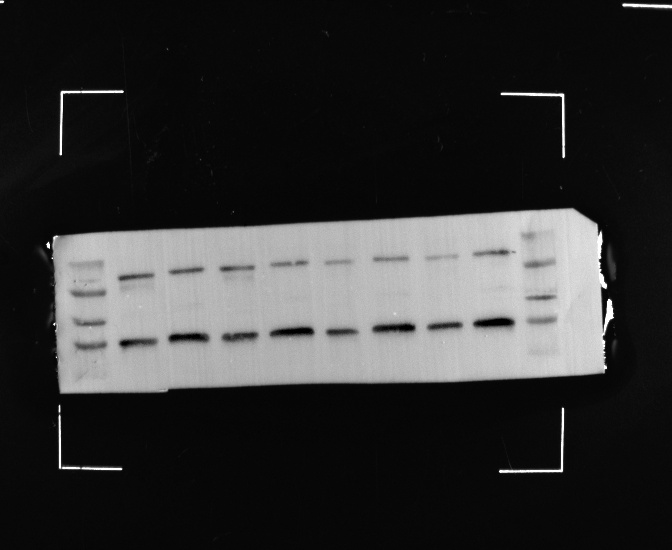

Supplement: Supplementary file 3 [file DataSheet3.zip › GSDME-4T1-2.jpg]

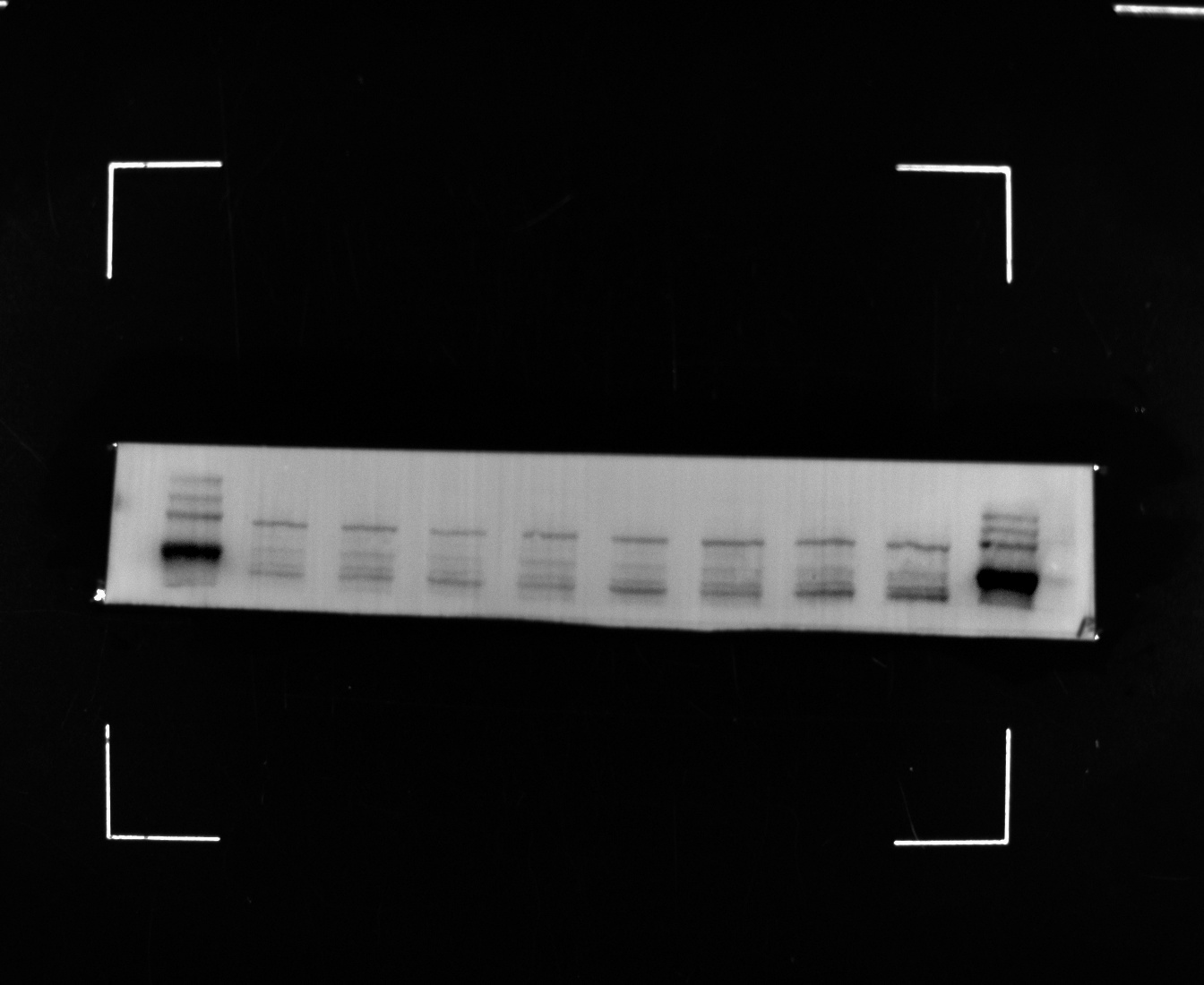

Supplement: Supplementary file 3 [file DataSheet3.zip › GSDME-4T1-3.jpg]

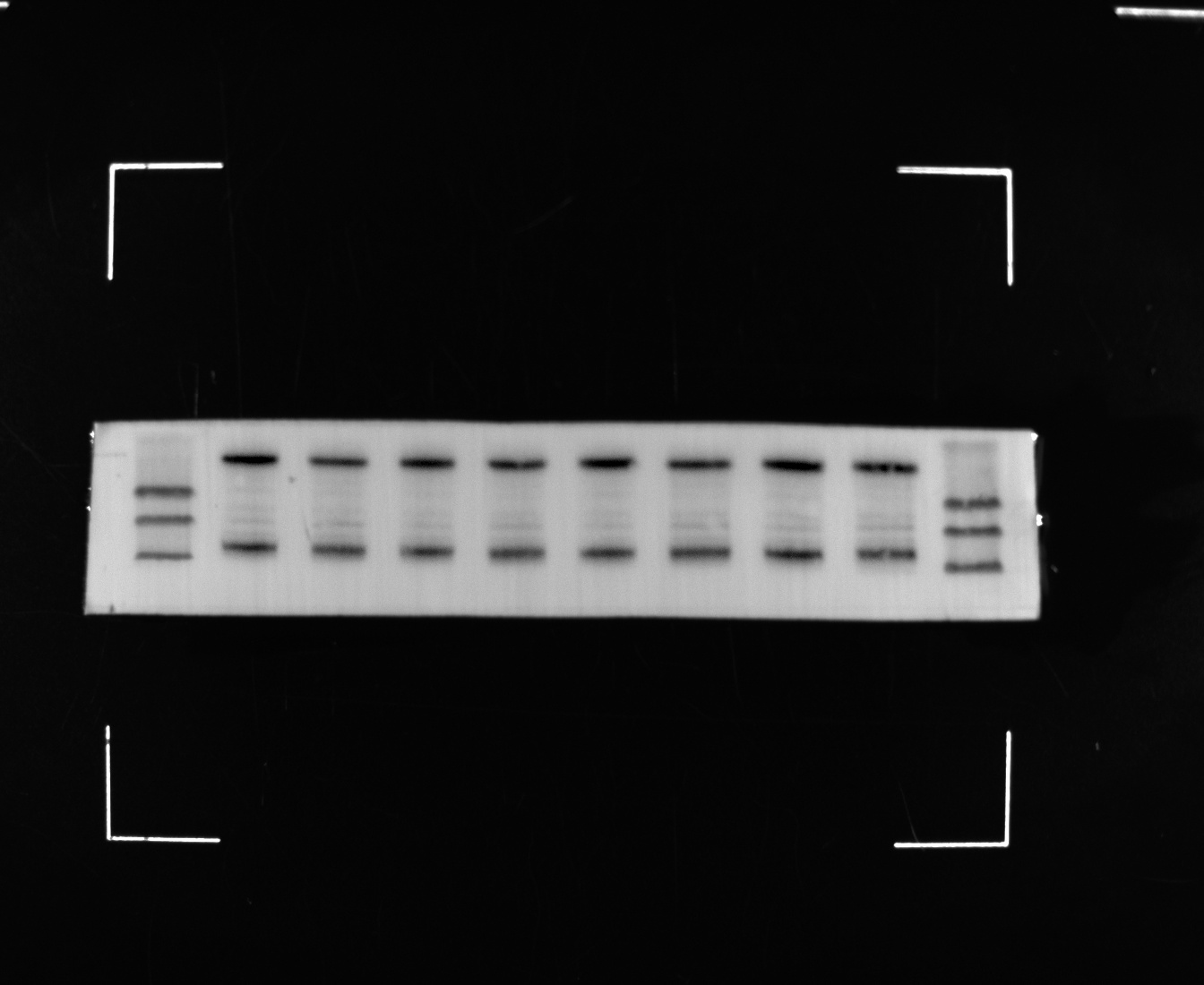

Supplement: Supplementary file 3 [file DataSheet3.zip › GSDME-EMT6-1.jpg]

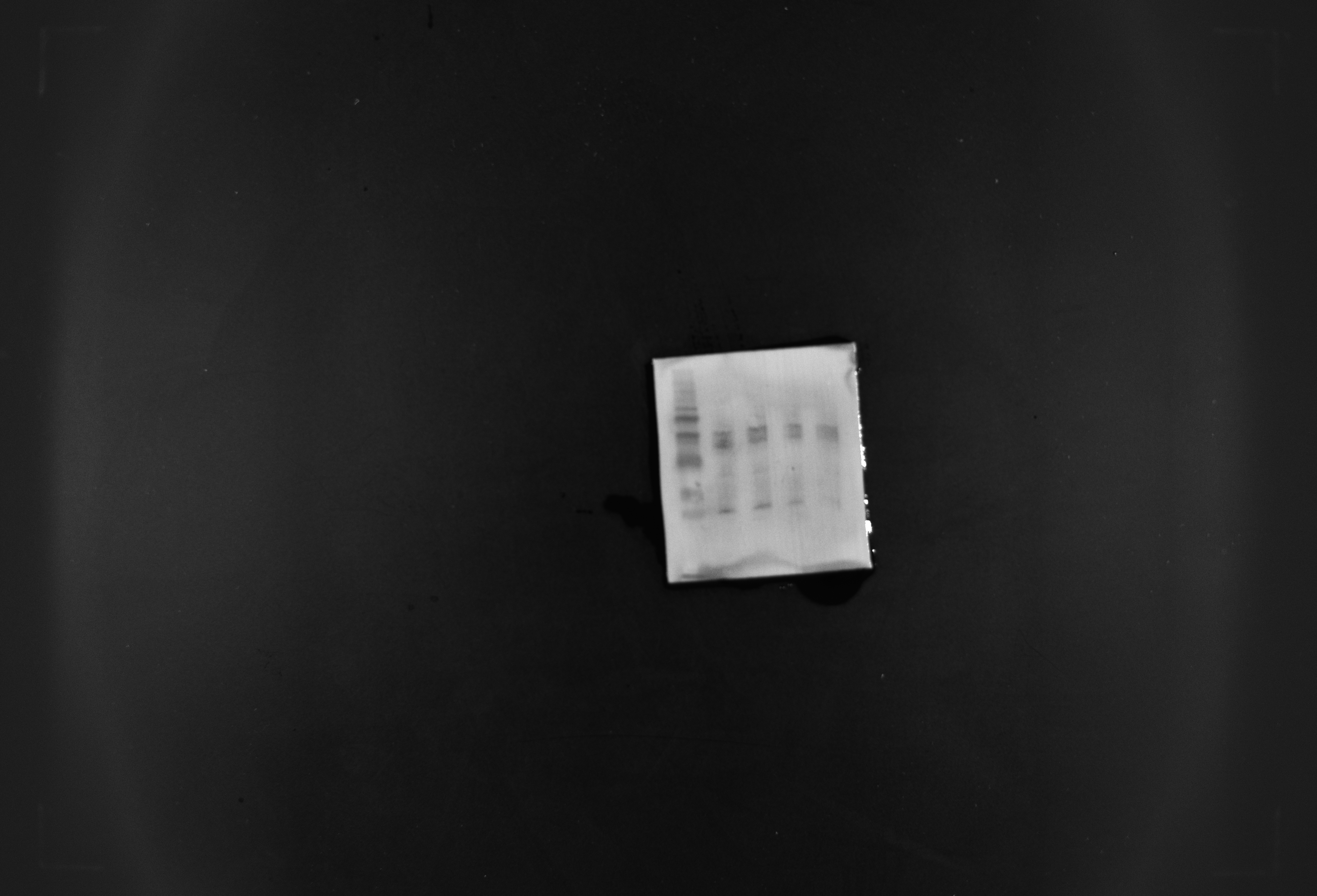

Supplement: Supplementary file 3 [file DataSheet3.zip › GSDME-EMT6-2.png]

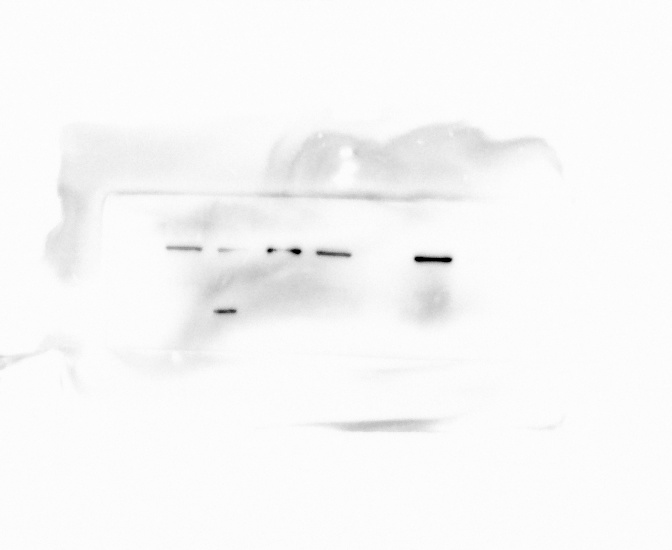

Supplement: Supplementary file 4 [file DataSheet4.zip › CAS-3-4T1-2.jpg]

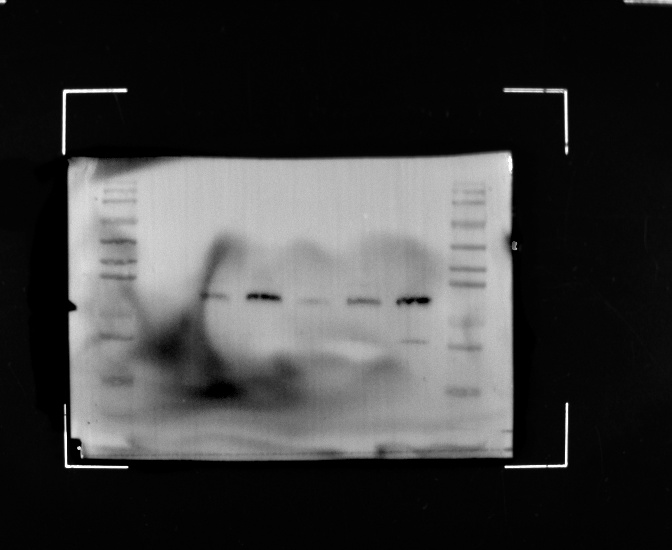

Supplement: Supplementary file 4 [file DataSheet4.zip › CAS-3-4T1-3.jpg]

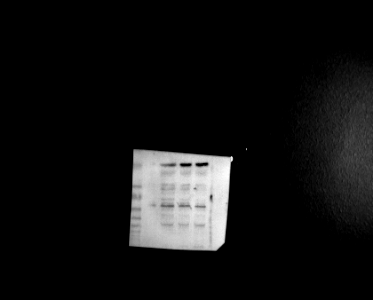

Supplement: Supplementary file 4 [file DataSheet4.zip › CAS-3-4T1.tif]

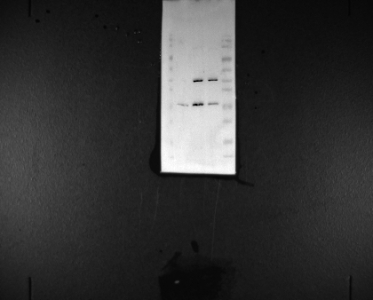

Supplement: Supplementary file 4 [file DataSheet4.zip › CAS-3-EMT6-2.jpg]

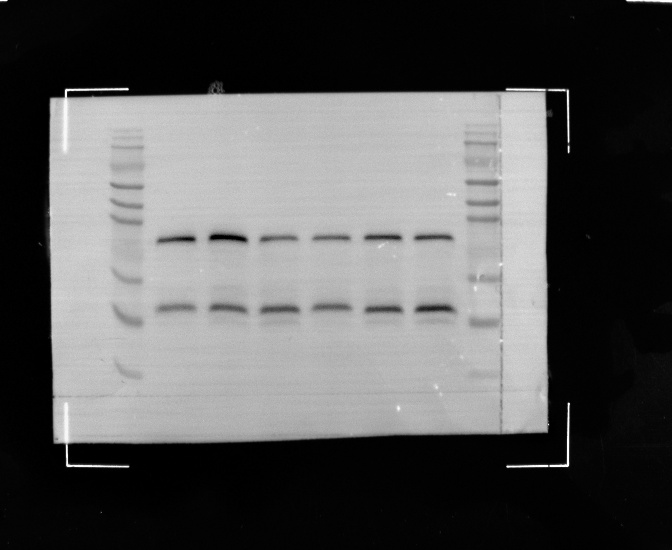

Supplement: Supplementary file 4 [file DataSheet4.zip › CAS-3-EMT6-3.jpg]

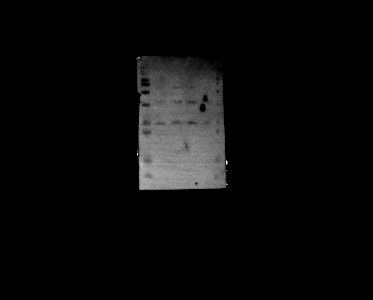

Supplement: Supplementary file 4 [file DataSheet4.zip › CAS-3-EMT6.jpg]

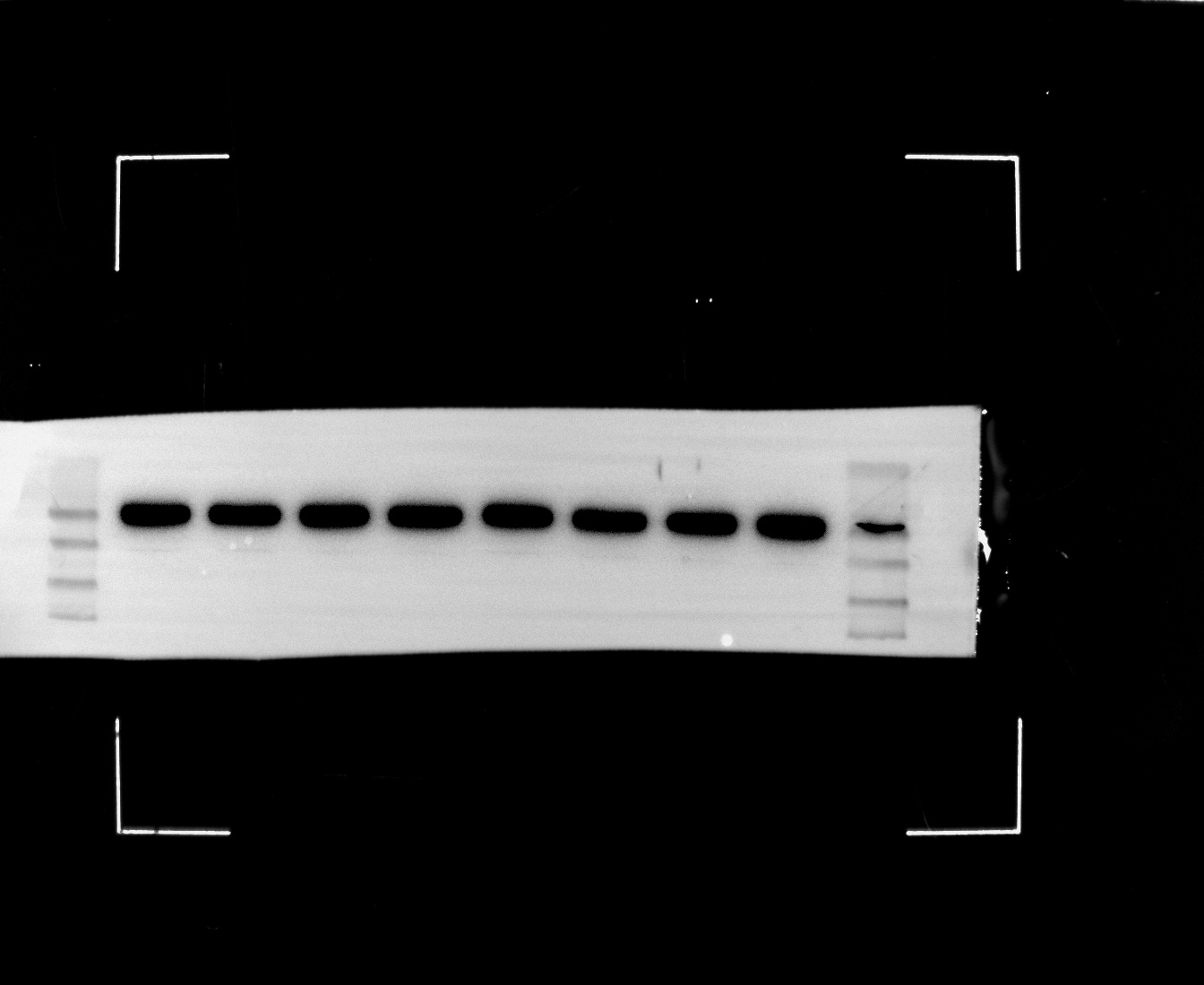

Supplement: Supplementary file 4 [file DataSheet4.zip › GAPDH-4.jpg]

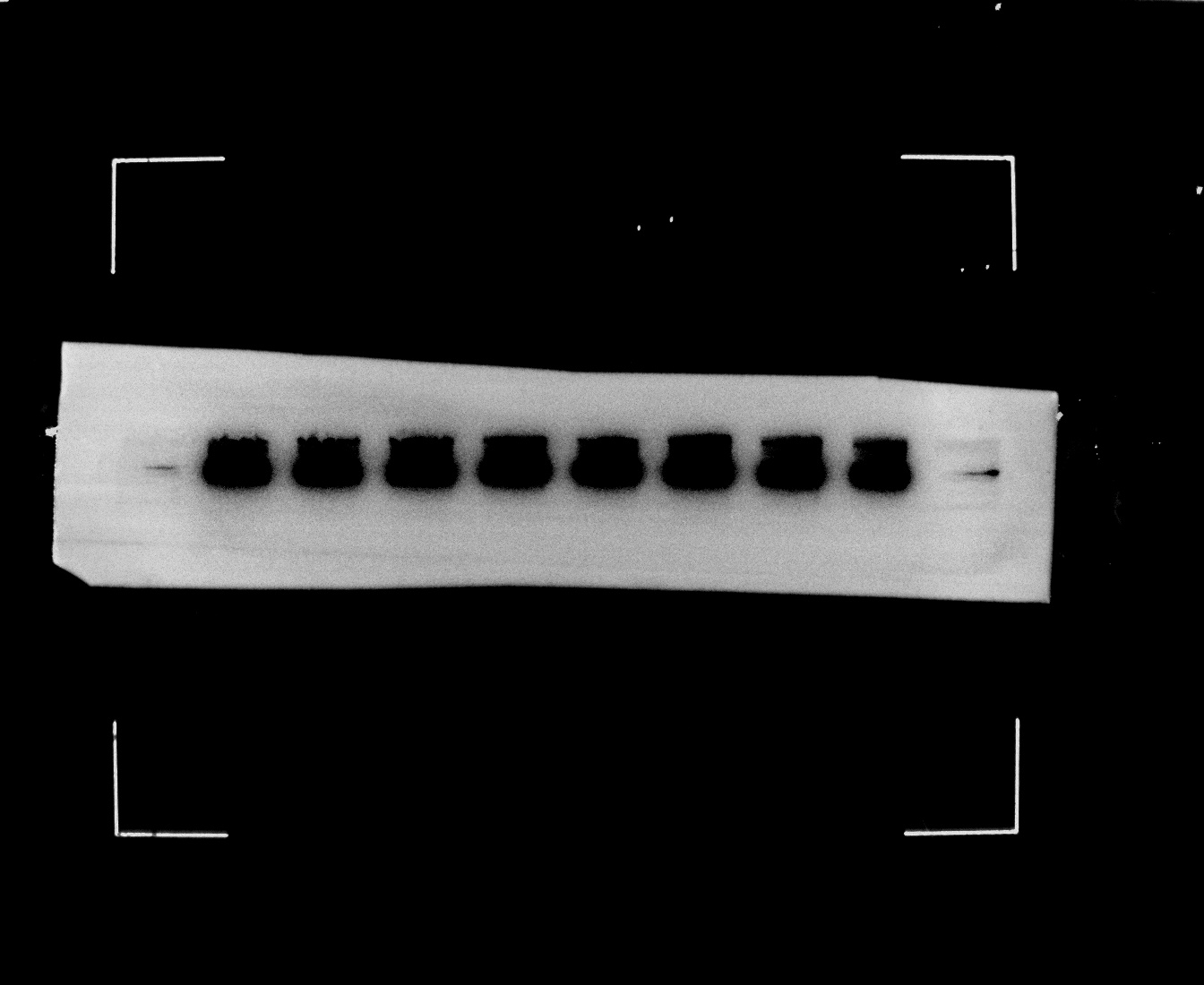

Supplement: Supplementary file 4 [file DataSheet4.zip › GAPDH-6.jpg]

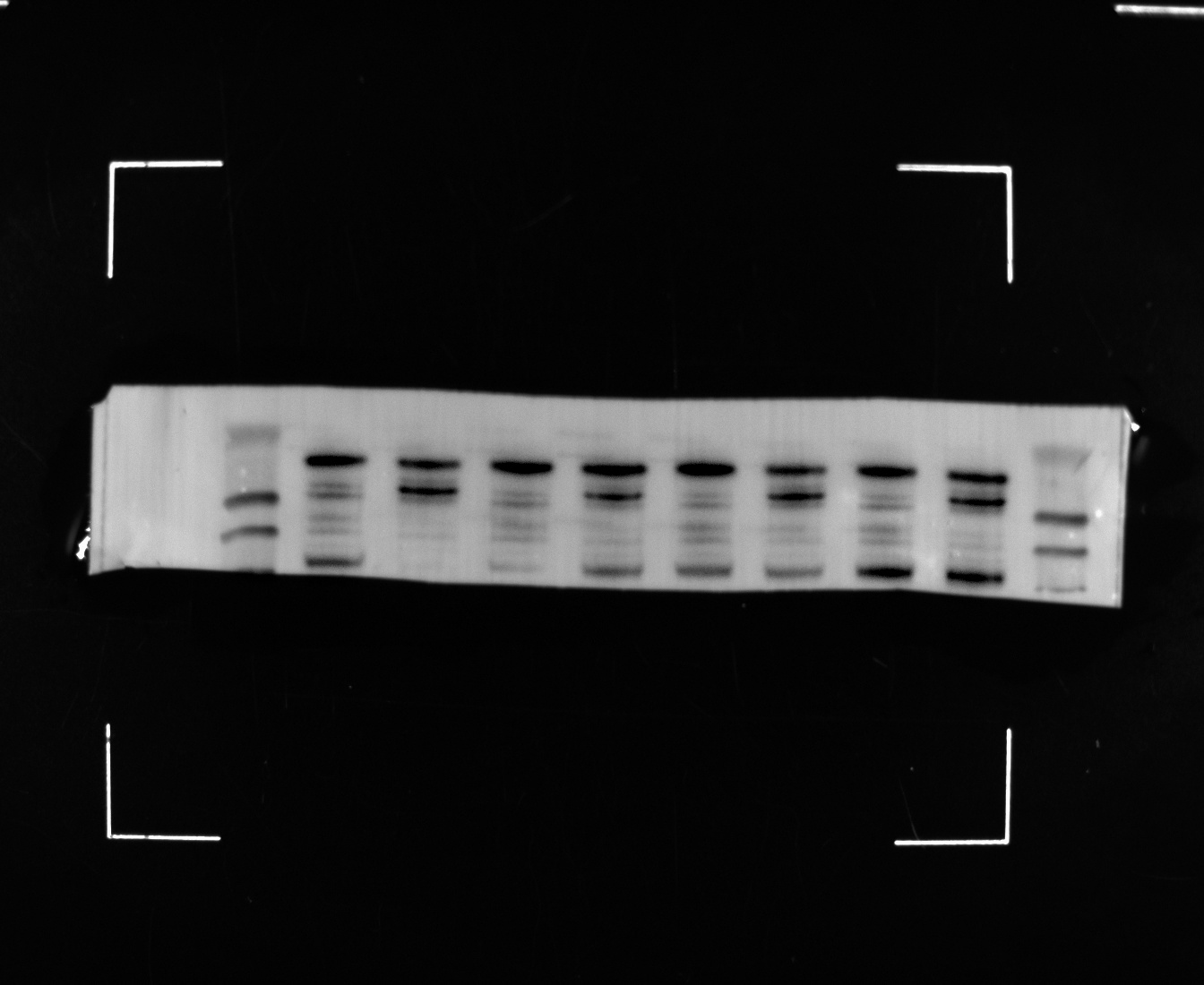

Supplement: Supplementary file 4 [file DataSheet4.zip › GSDME-4T1-1.jpg]

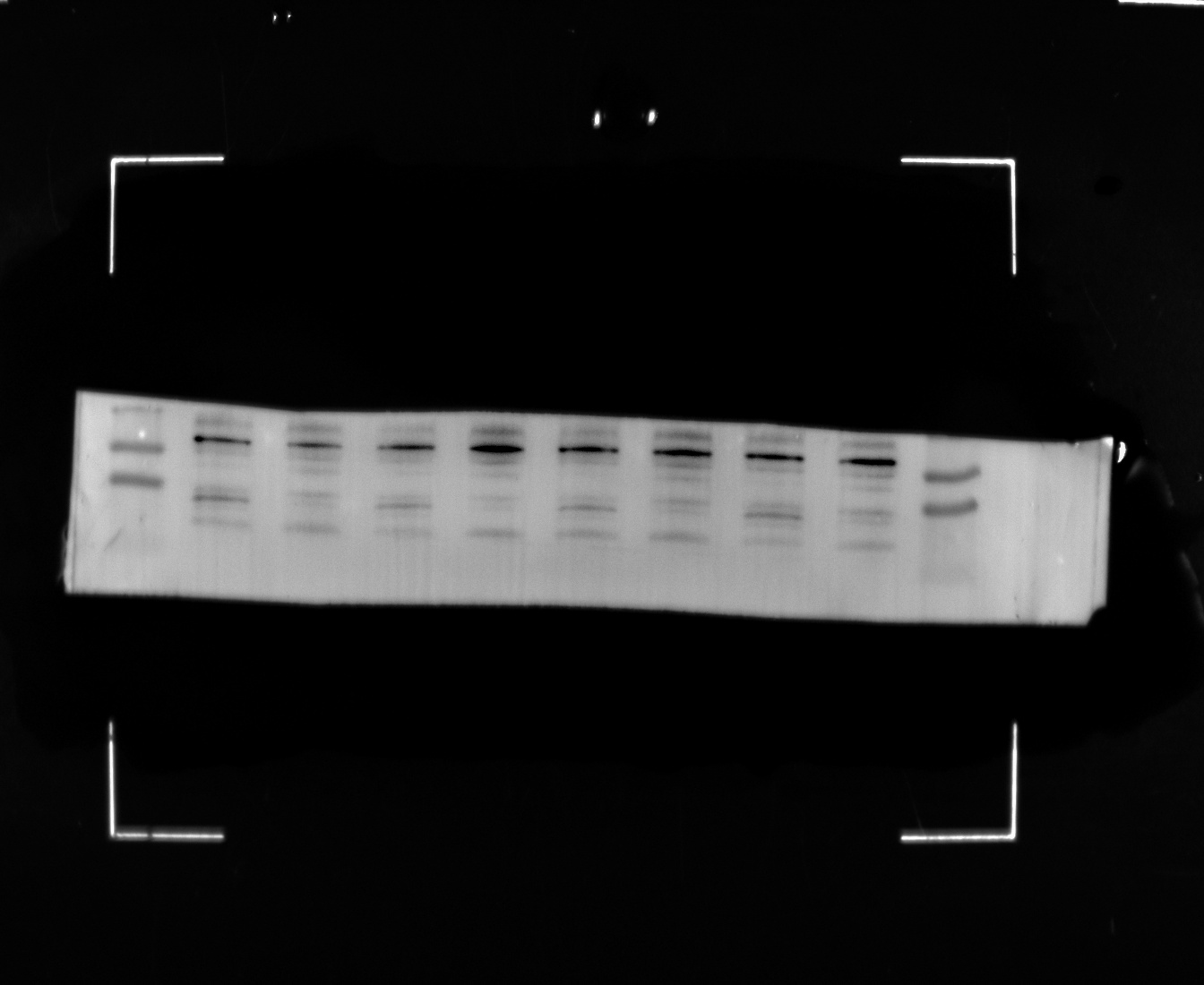

Supplement: Supplementary file 4 [file DataSheet4.zip › GSDME-EMT6-2.jpg]

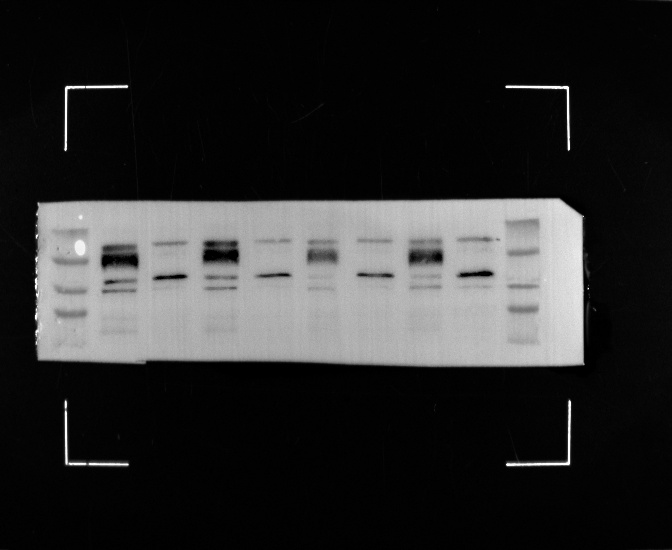

Supplement: Supplementary file 4 [file DataSheet4.zip › GSDME-EMT6.jpg]

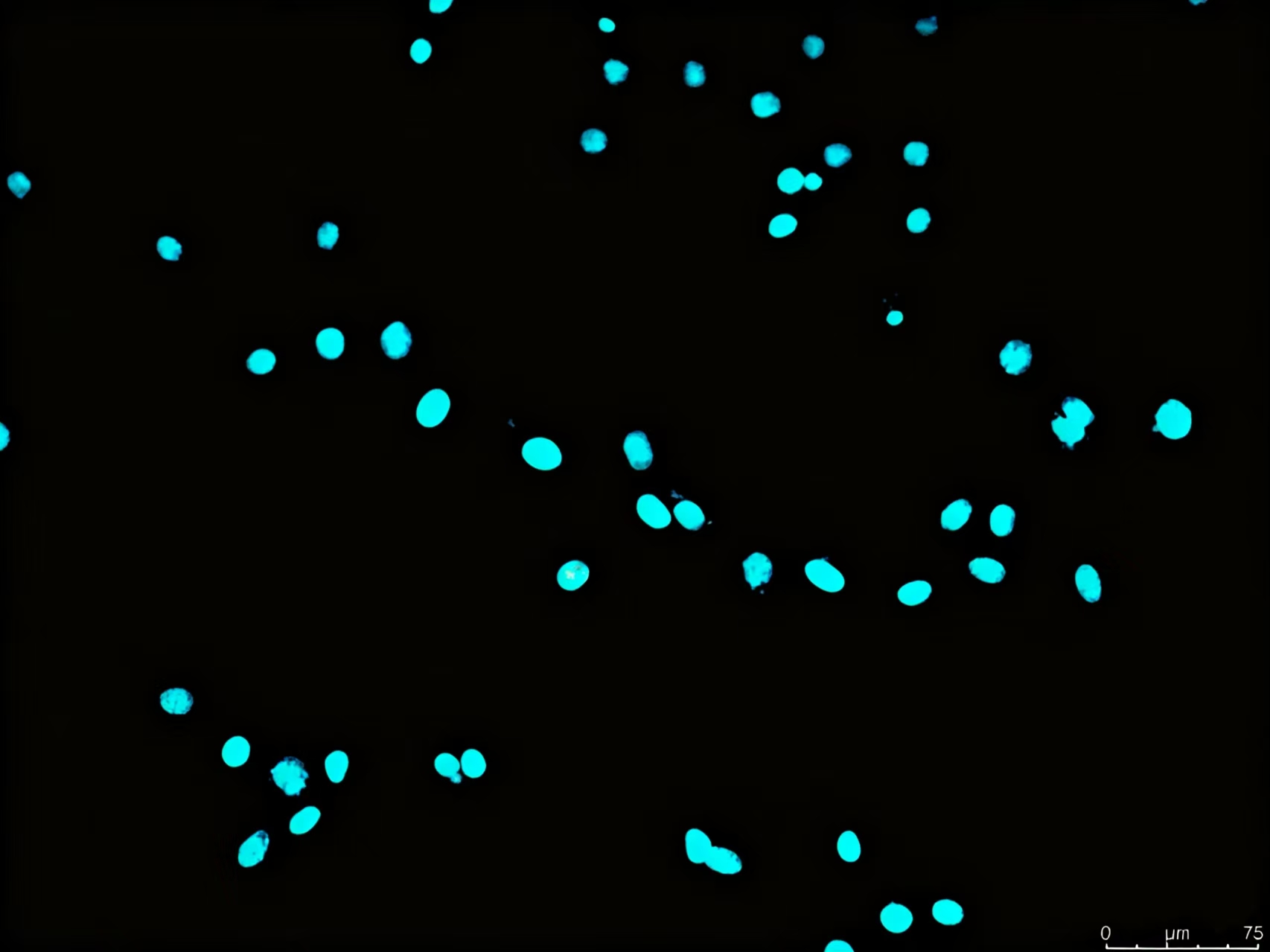

Supplement: Supplementary file 5 [file DataSheet5.zip › IF/1.jpg]

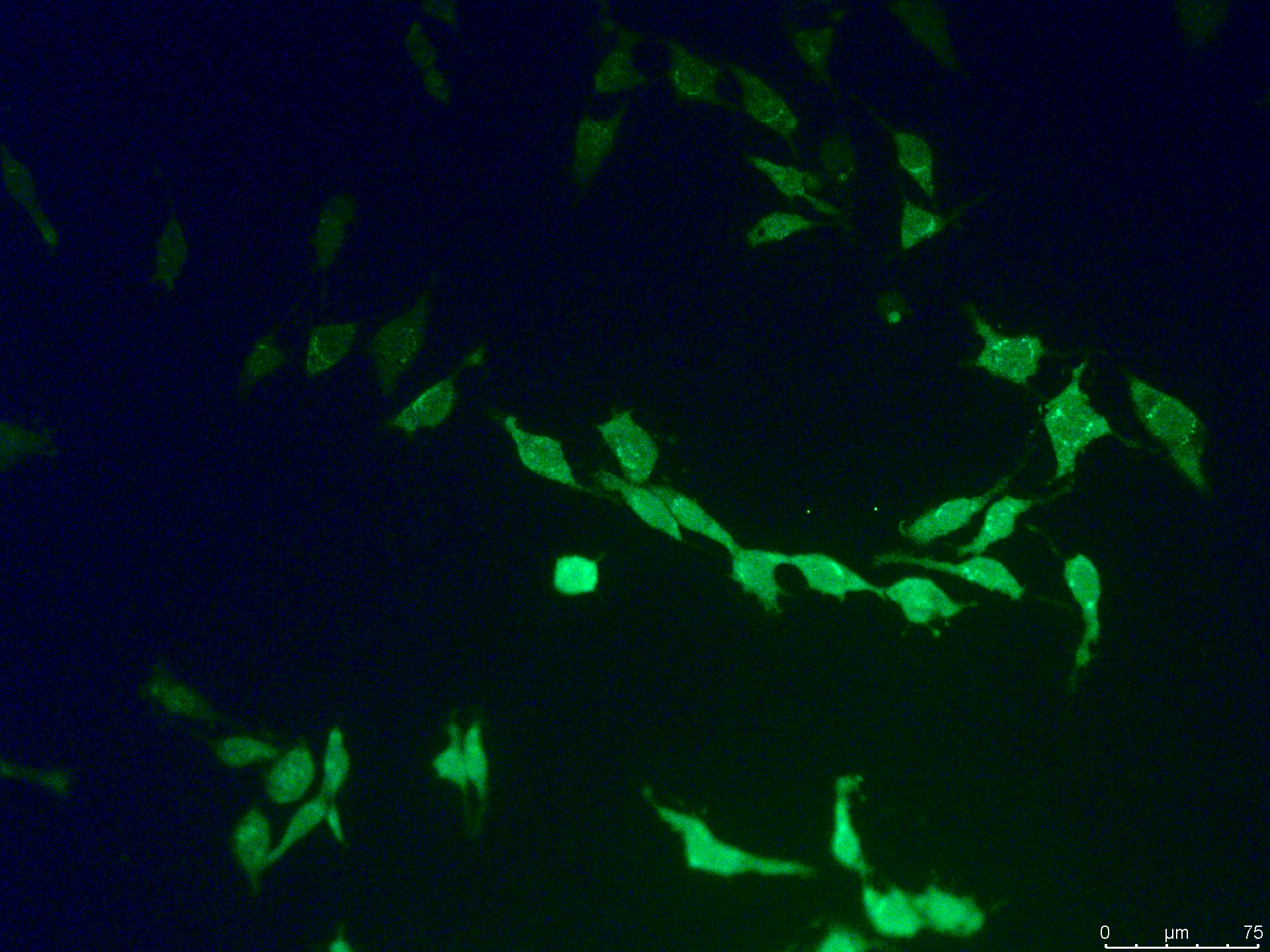

Supplement: Supplementary file 5 [file DataSheet5.zip › IF/2.jpg]

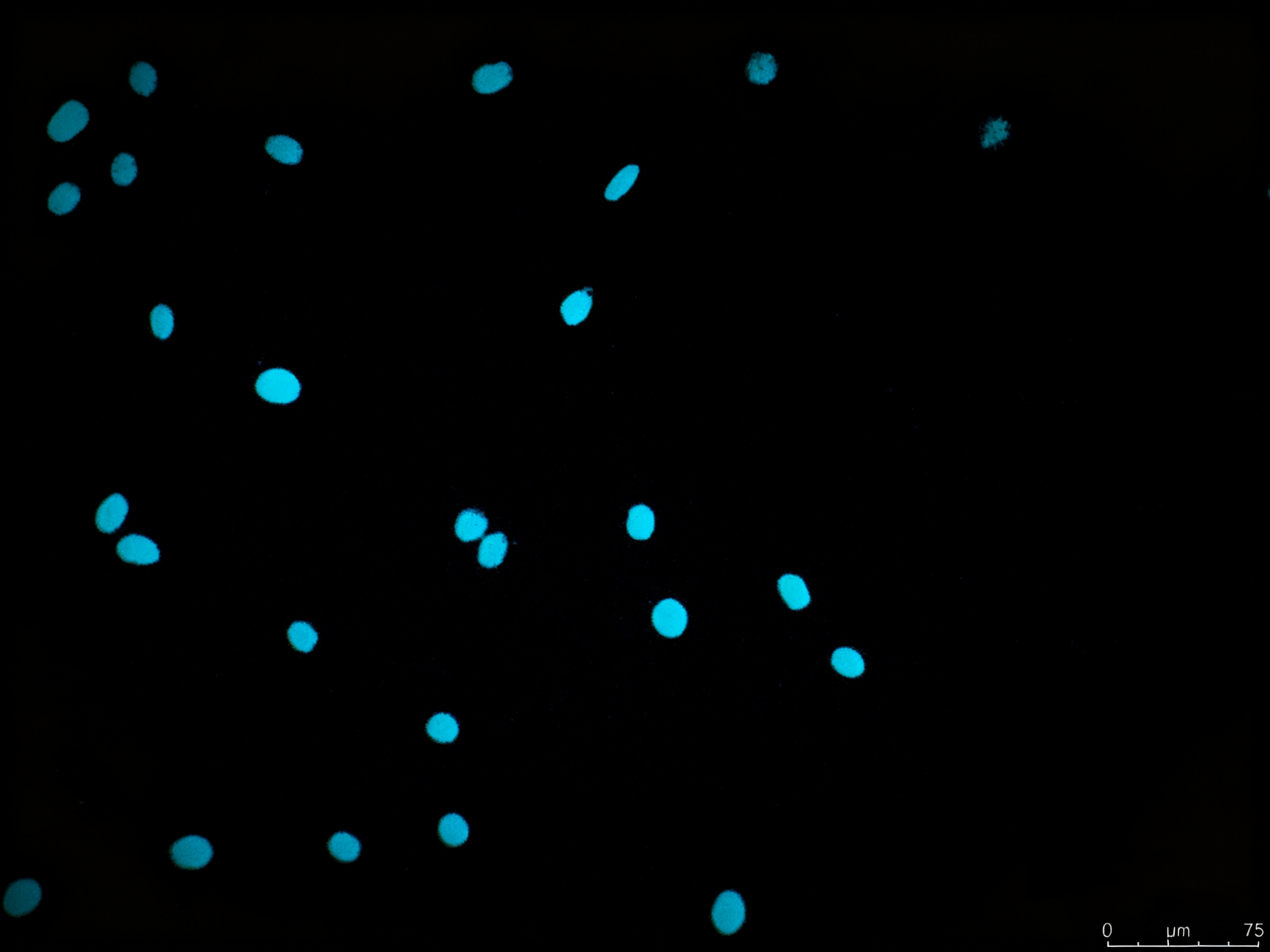

Supplement: Supplementary file 5 [file DataSheet5.zip › IF/3.jpg]

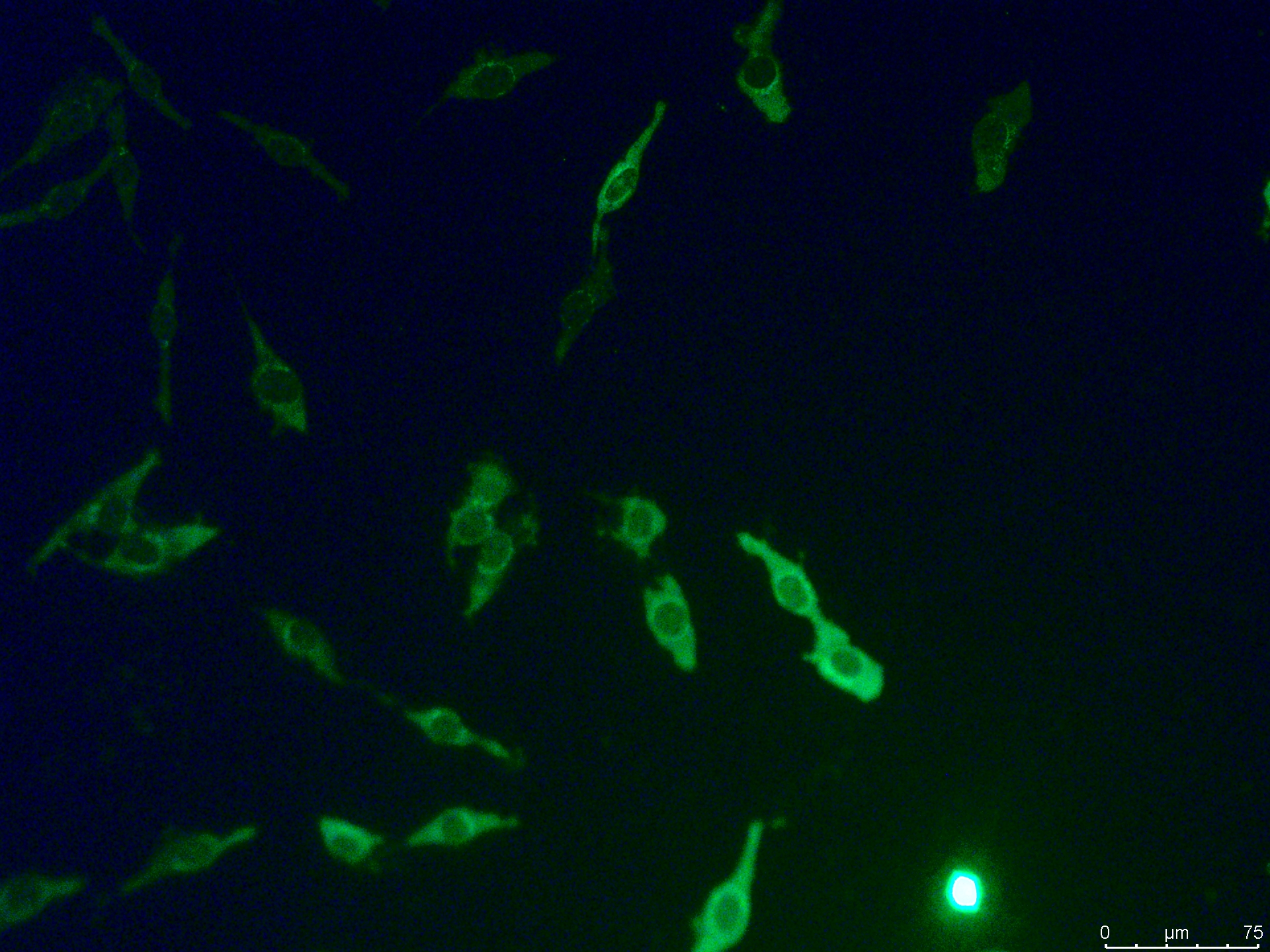

Supplement: Supplementary file 5 [file DataSheet5.zip › IF/4.jpg]

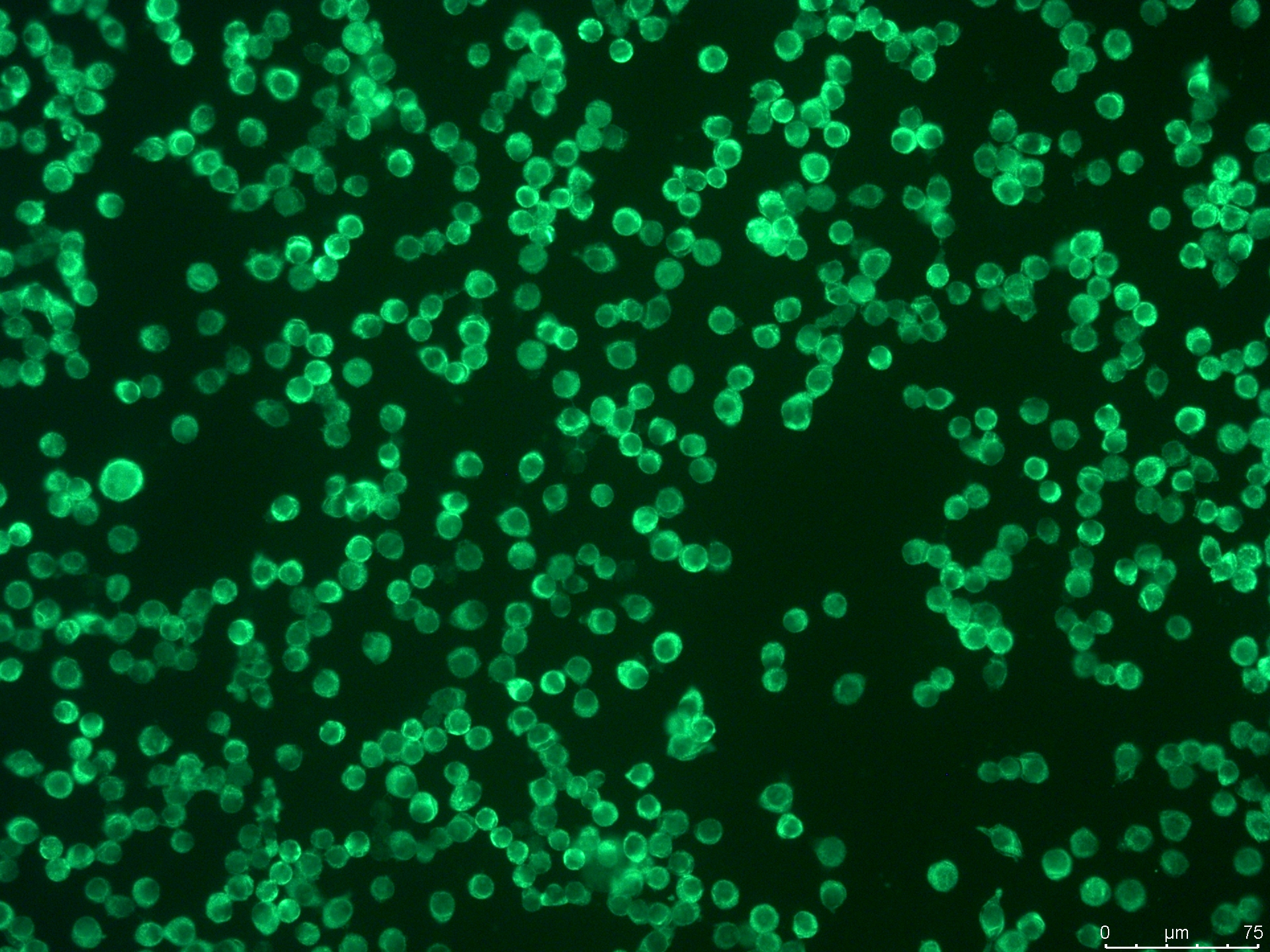

Supplement: Supplementary file 5 [file DataSheet5.zip › IF/5.jpg]

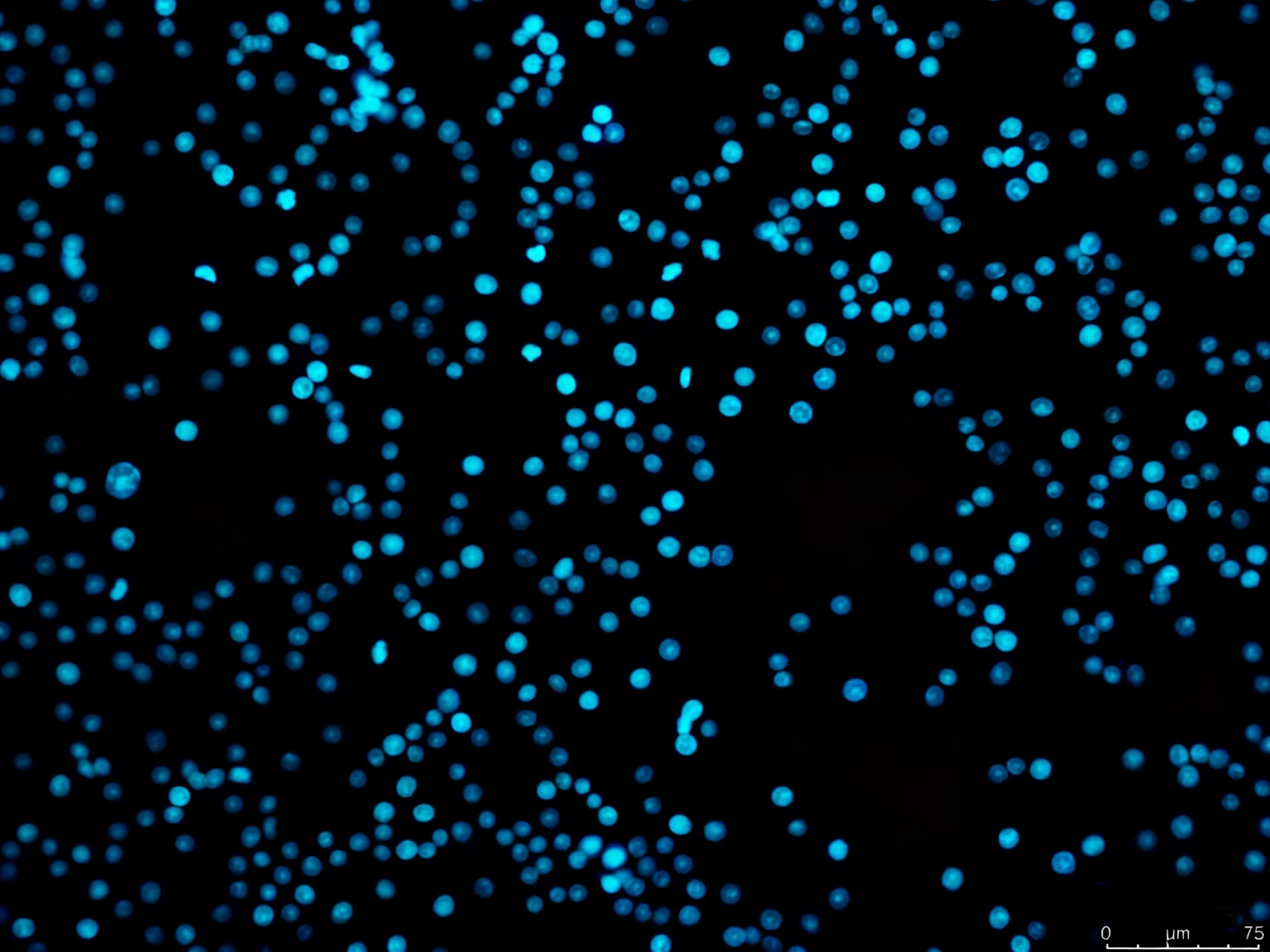

Supplement: Supplementary file 5 [file DataSheet5.zip › IF/6.jpg]

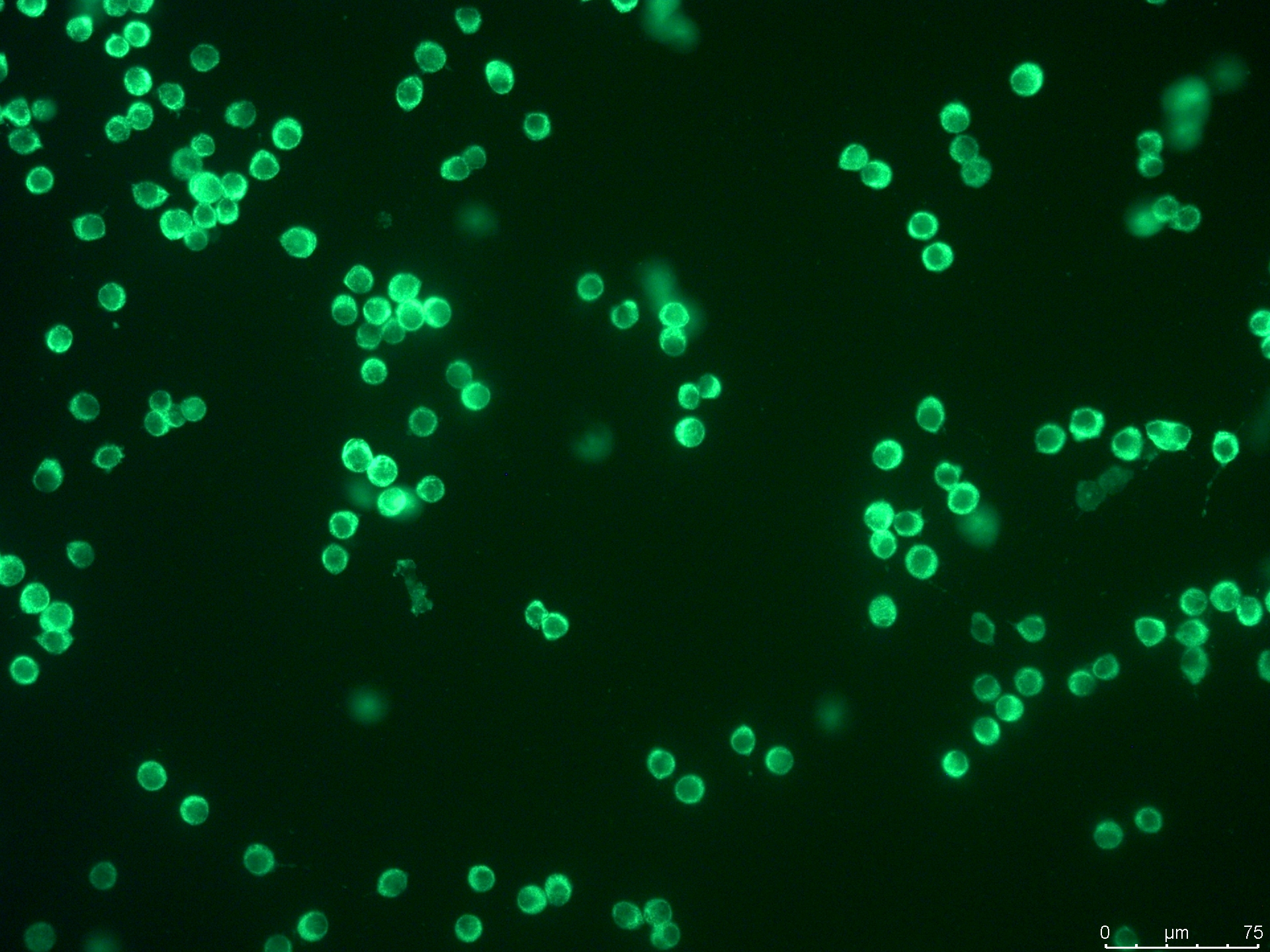

Supplement: Supplementary file 5 [file DataSheet5.zip › IF/7.jpg]

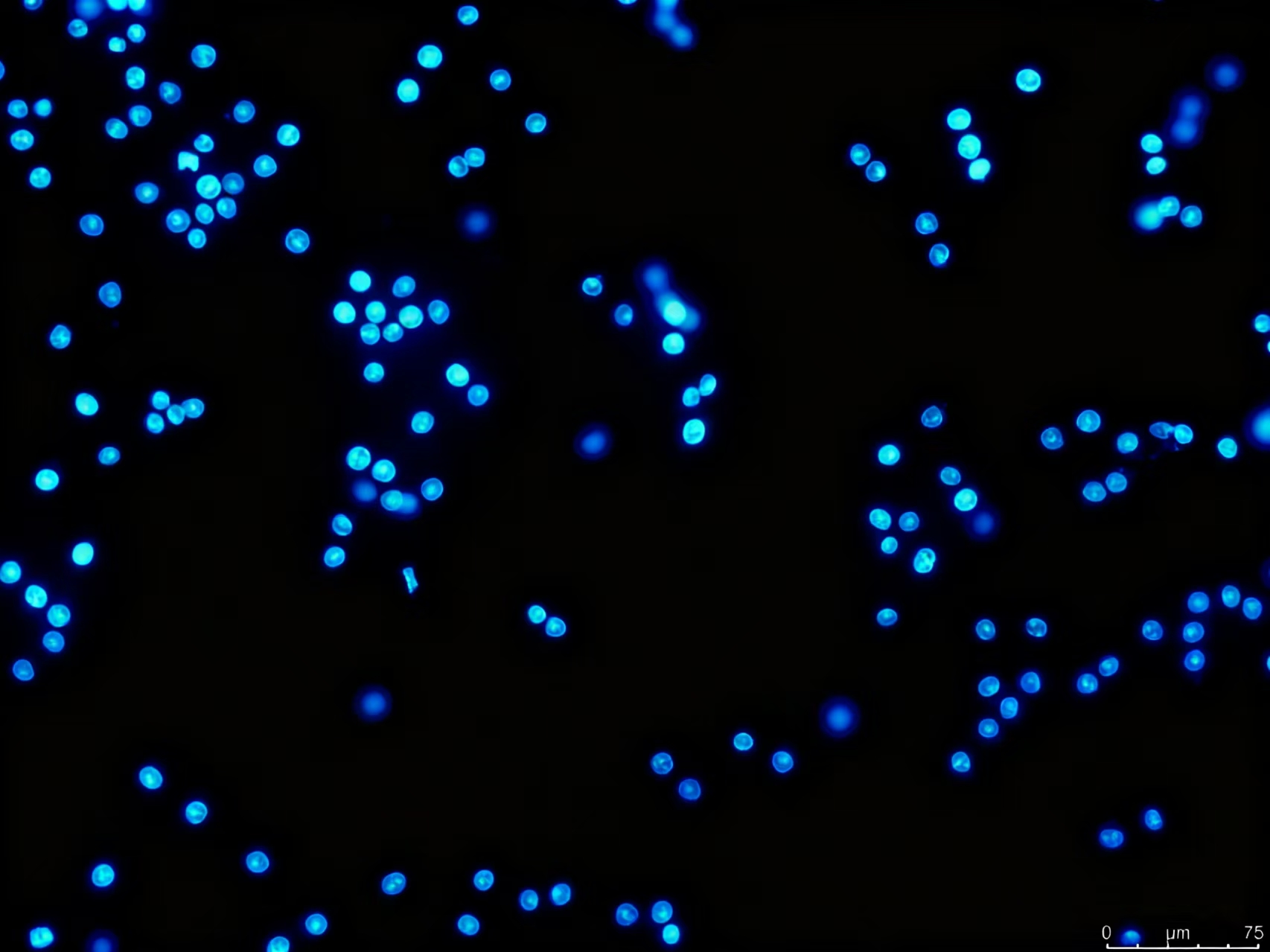

Supplement: Supplementary file 5 [file DataSheet5.zip › IF/Project001_Series025(1).jpg]

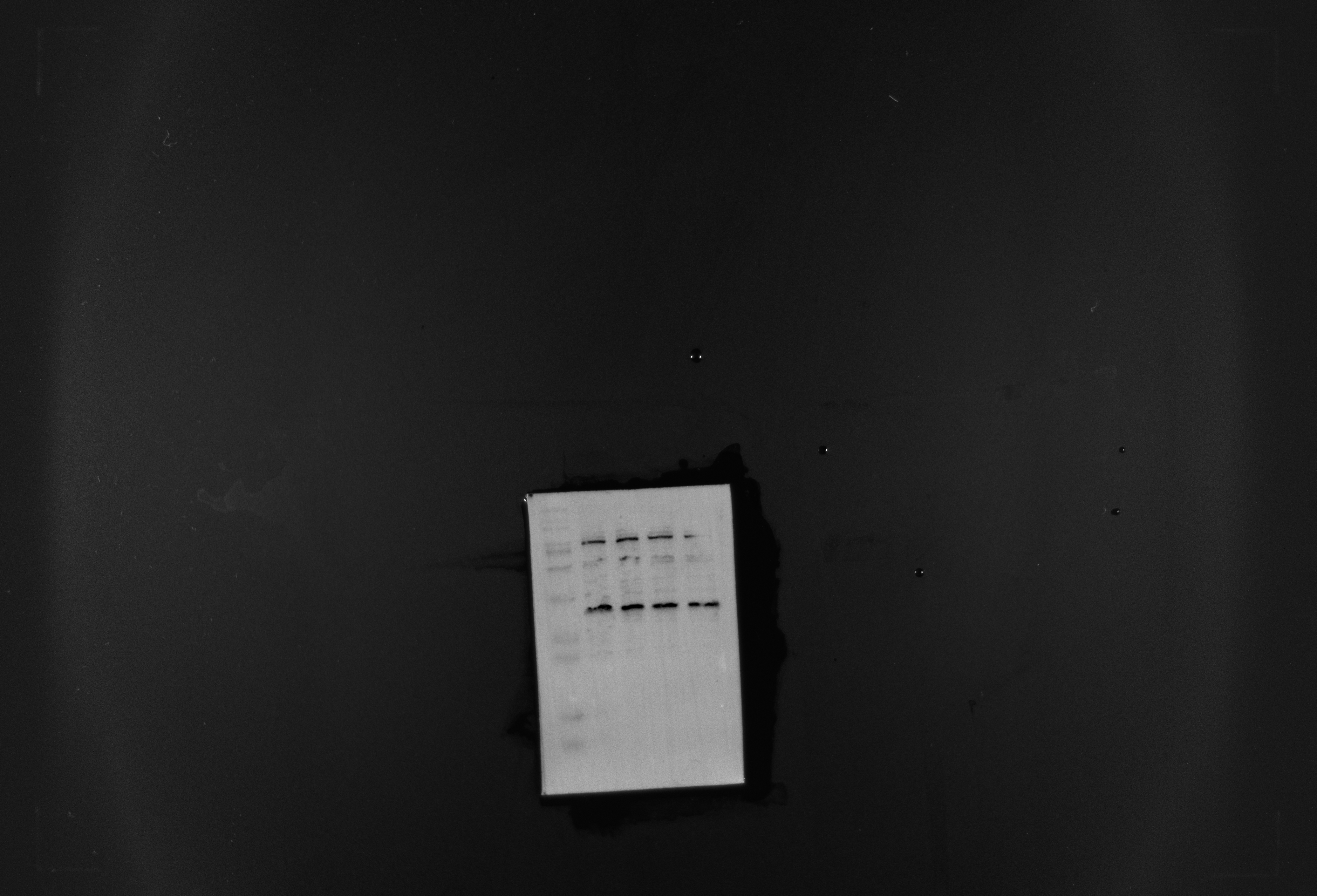

Supplement: Supplementary file 6 [file Image1.tif]

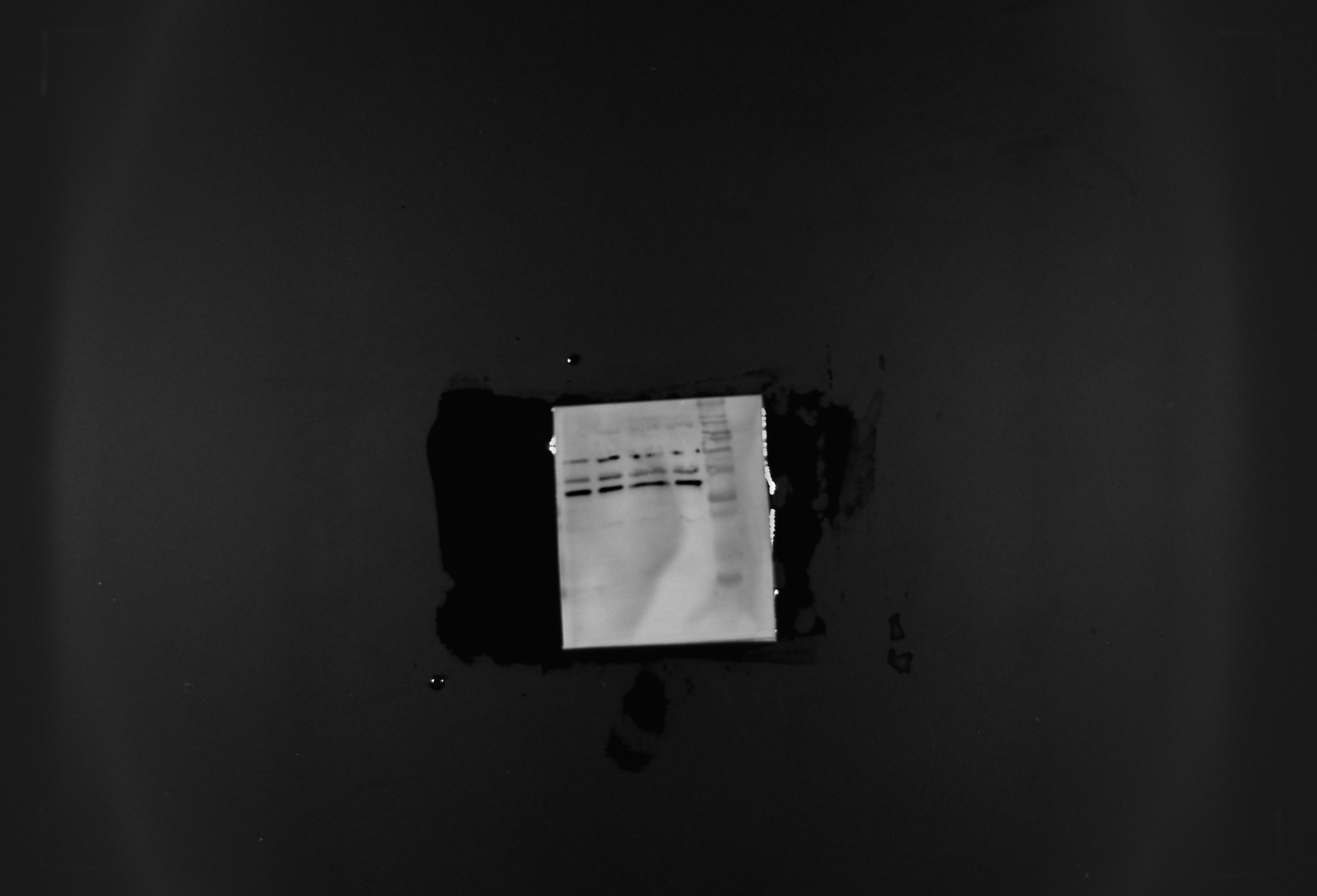

Supplement: Supplementary file 7 [file Image2.tif]

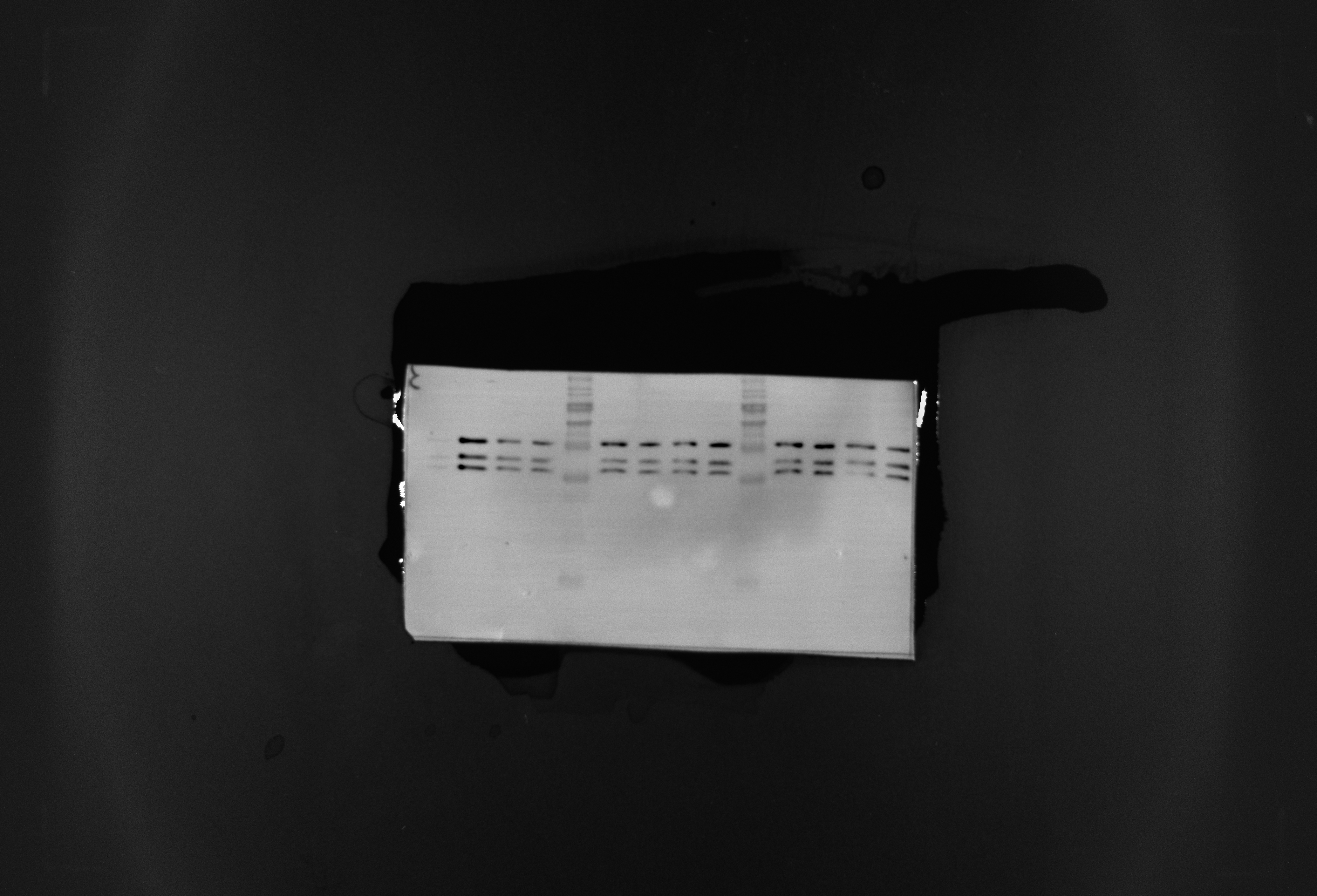

Supplement: Supplementary file 8 [file Image3.tif]

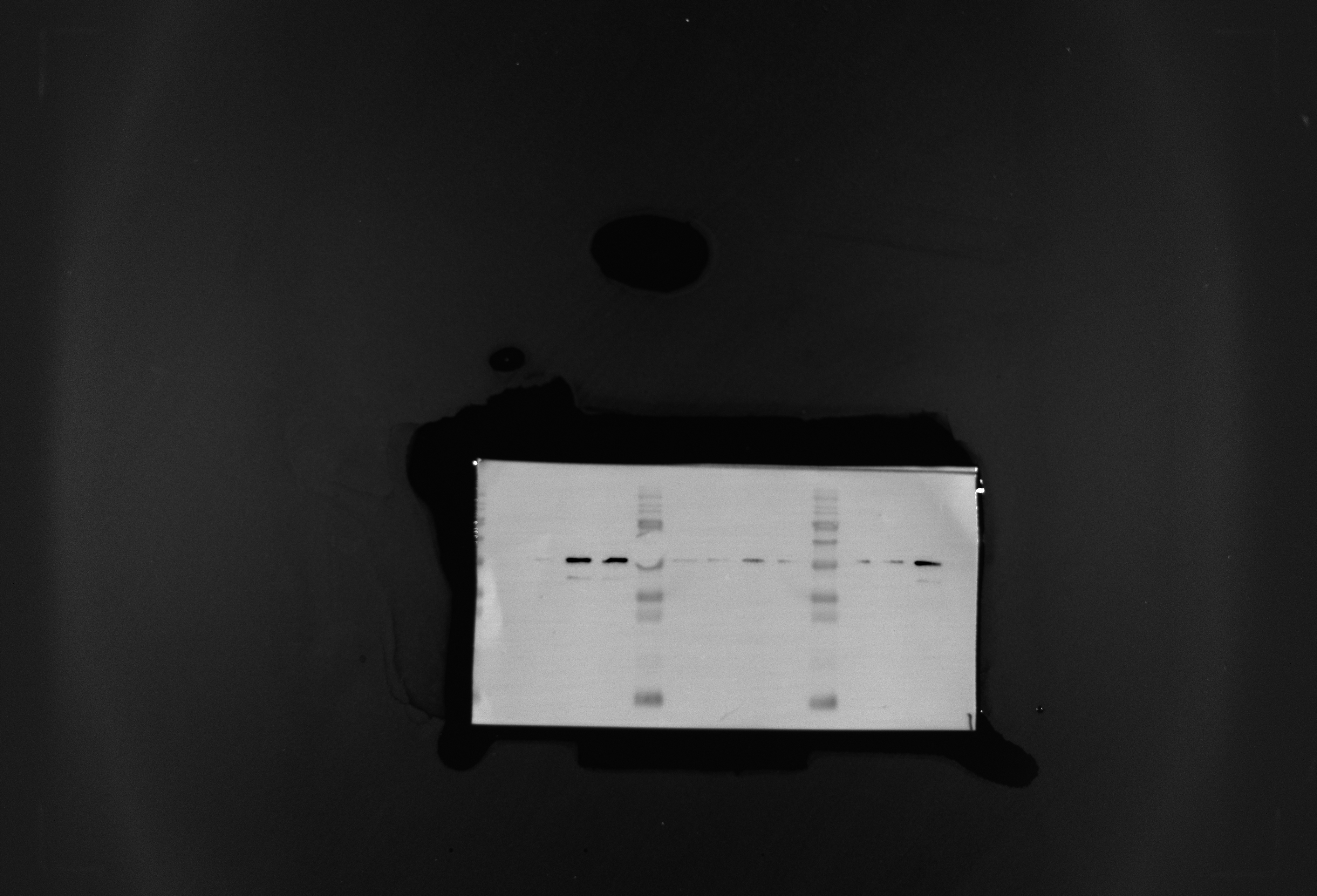

Supplement: Supplementary file 9 [file Image4.tif]

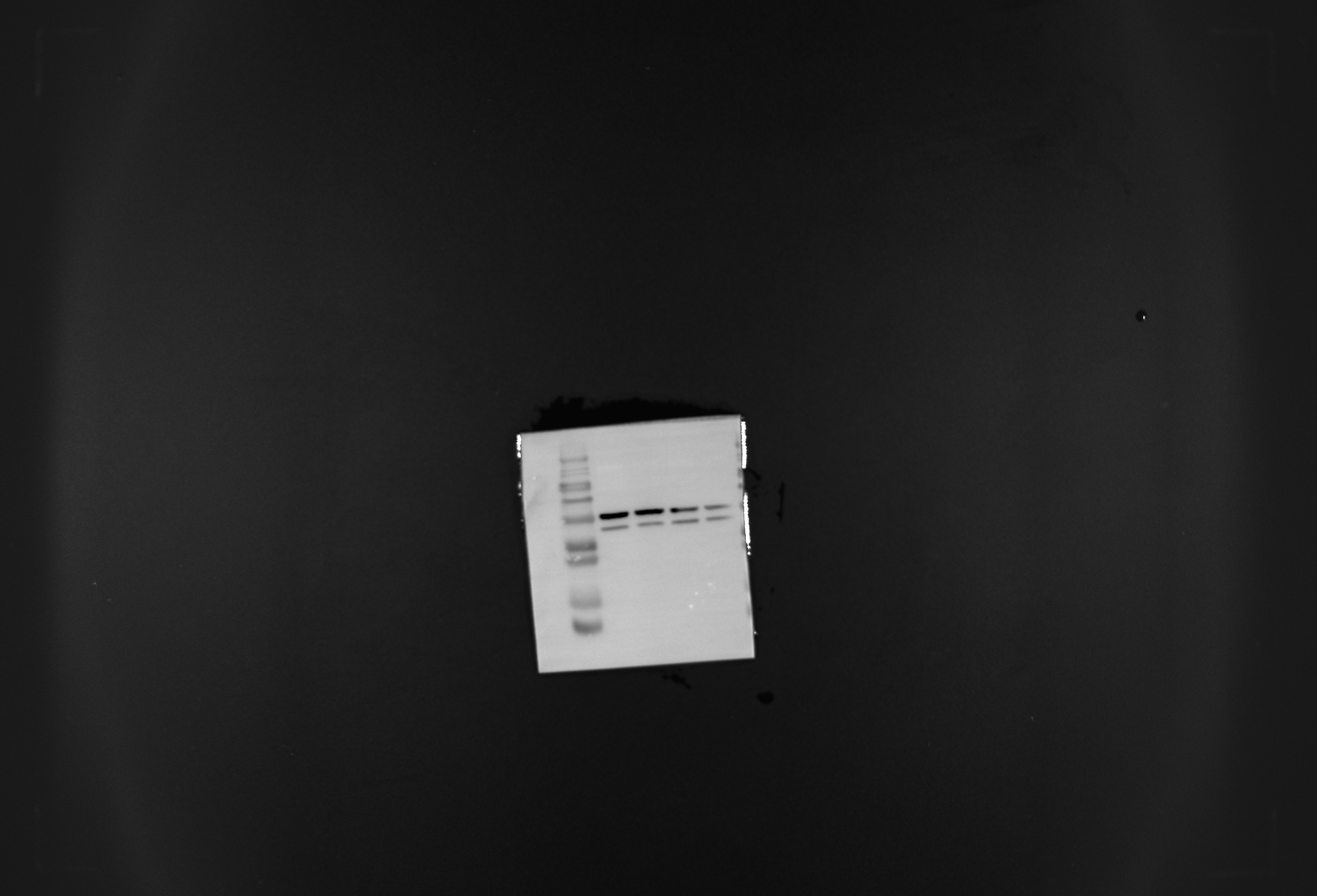

Supplement: Supplementary file 10 [file Image5.tif]

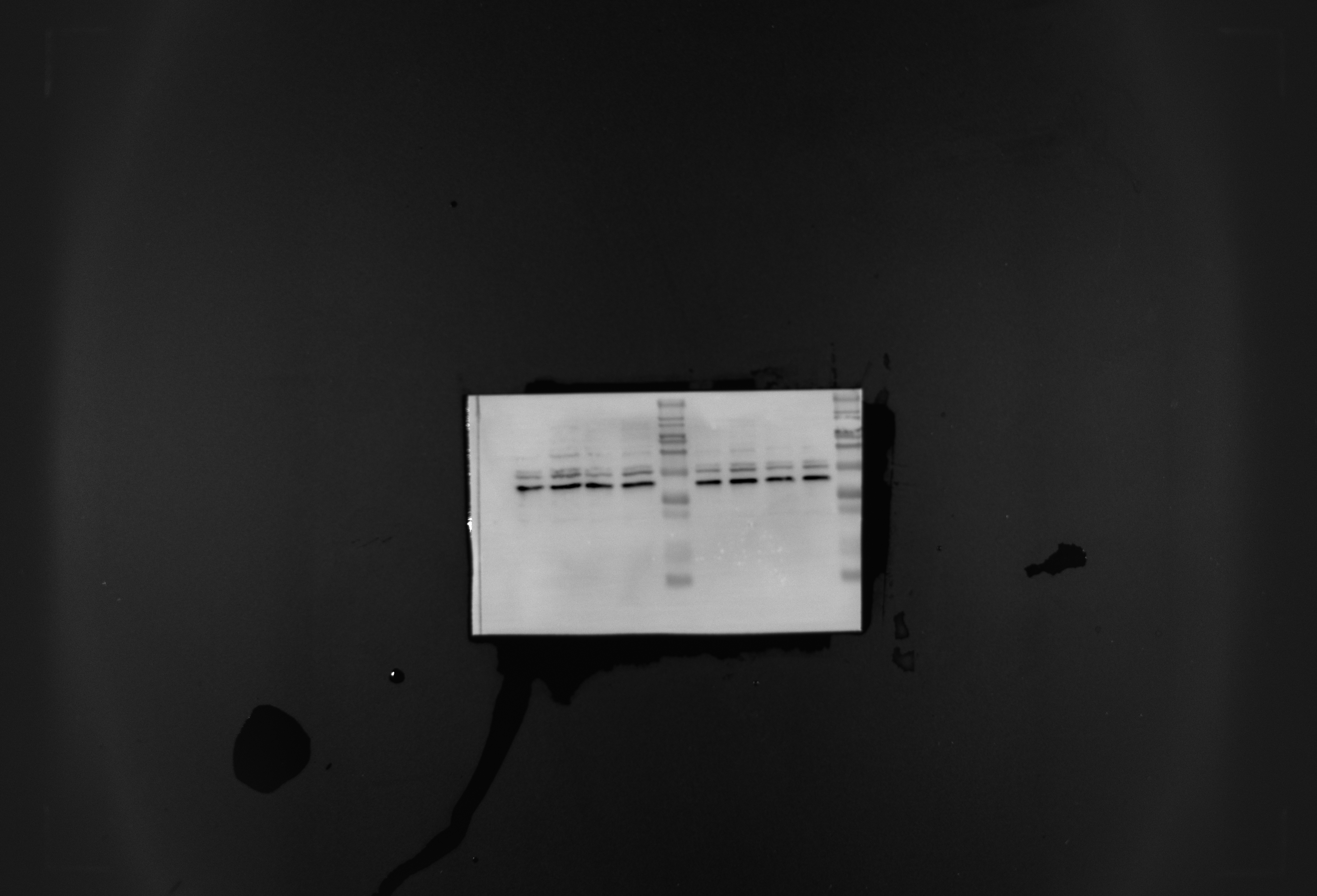

Supplement: Supplementary file 11 [file Image6.tif]

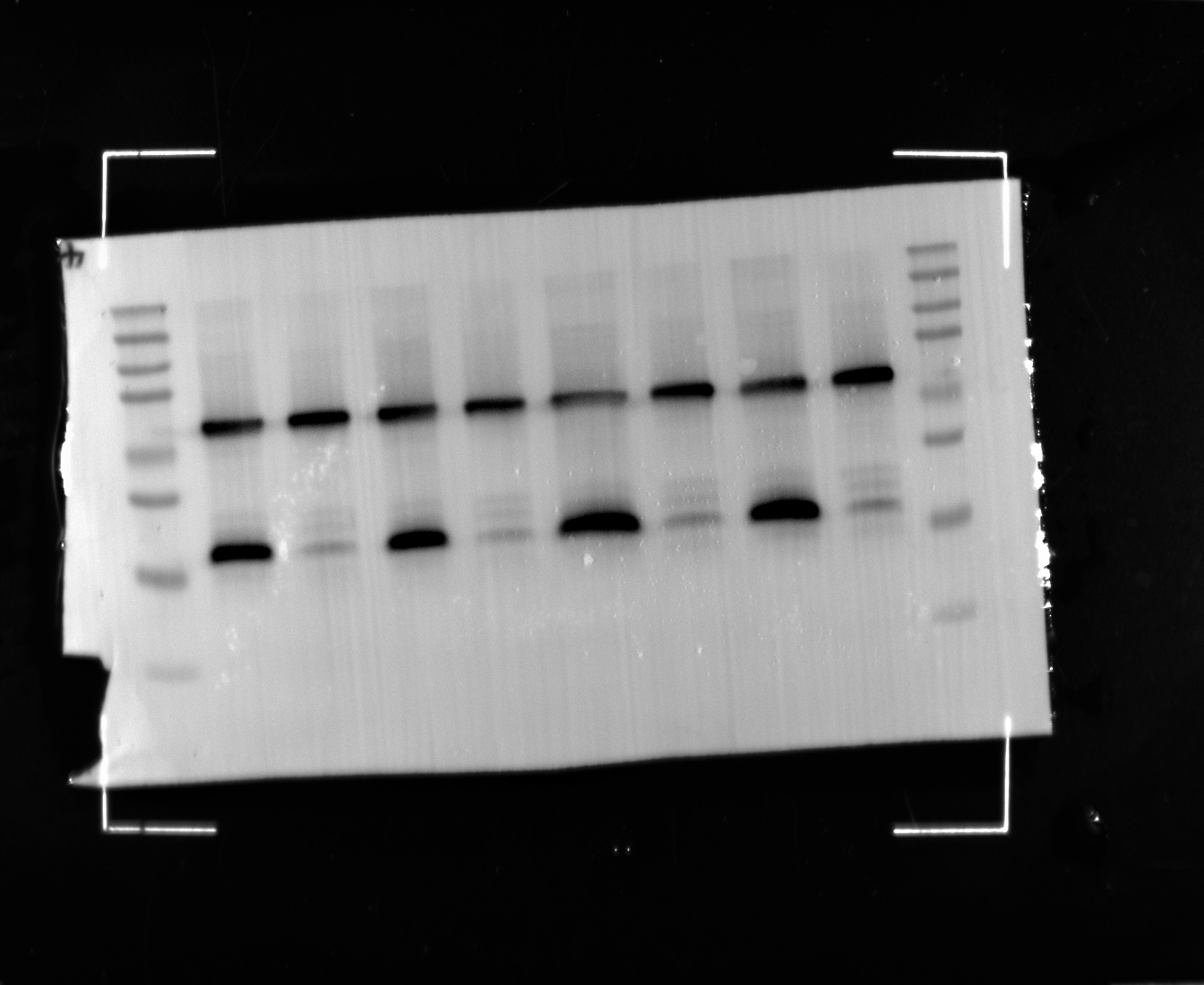

Supplement: Supplementary file 12 [file Image7.jpeg]

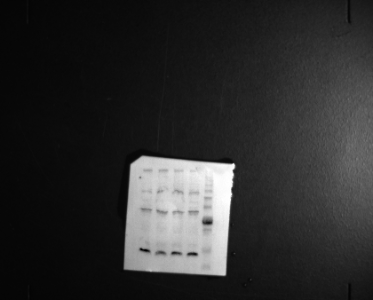

Supplement: Supplementary file 13 [file Image8.tif]

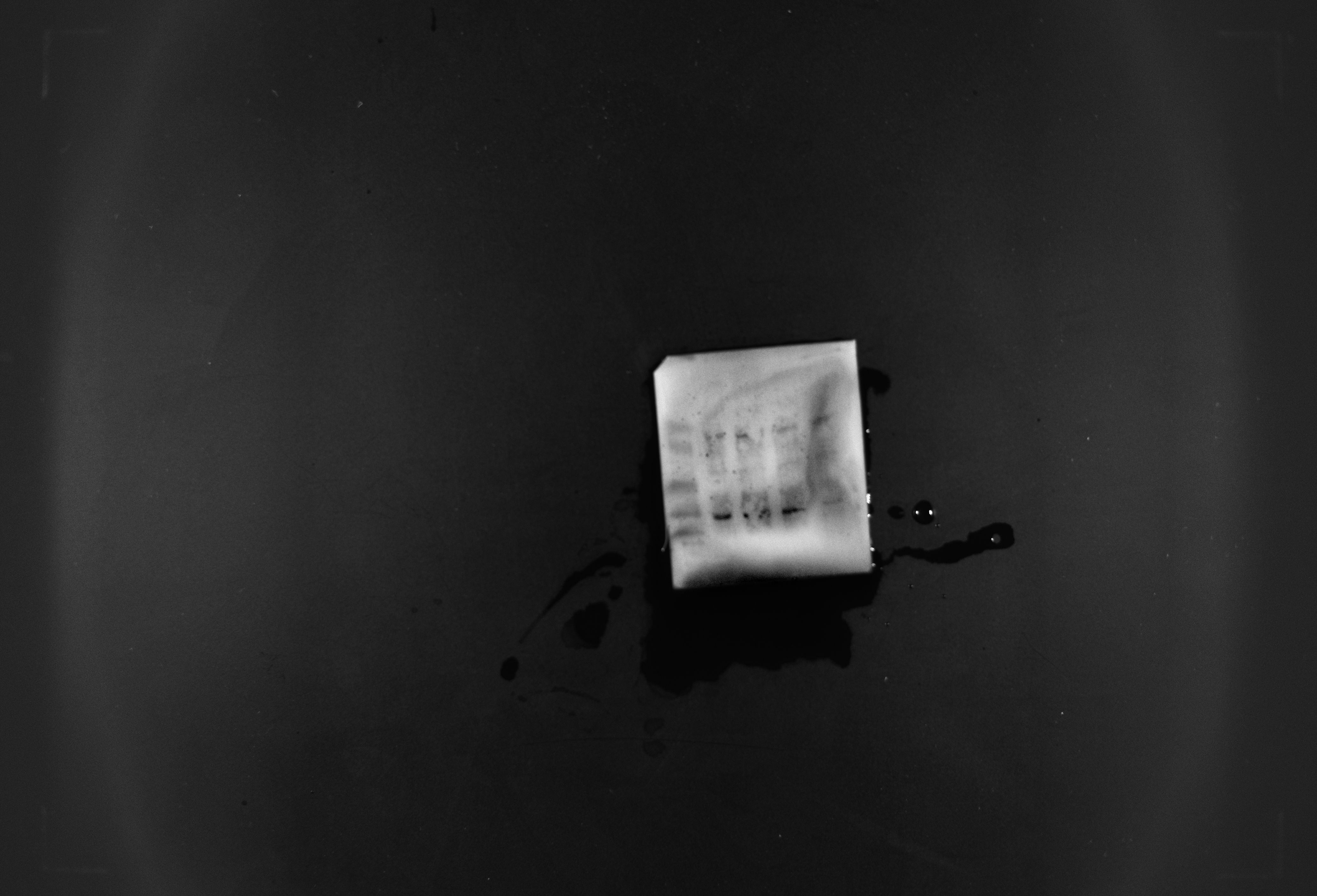

Supplement: Supplementary file 14 [file Image9.png]

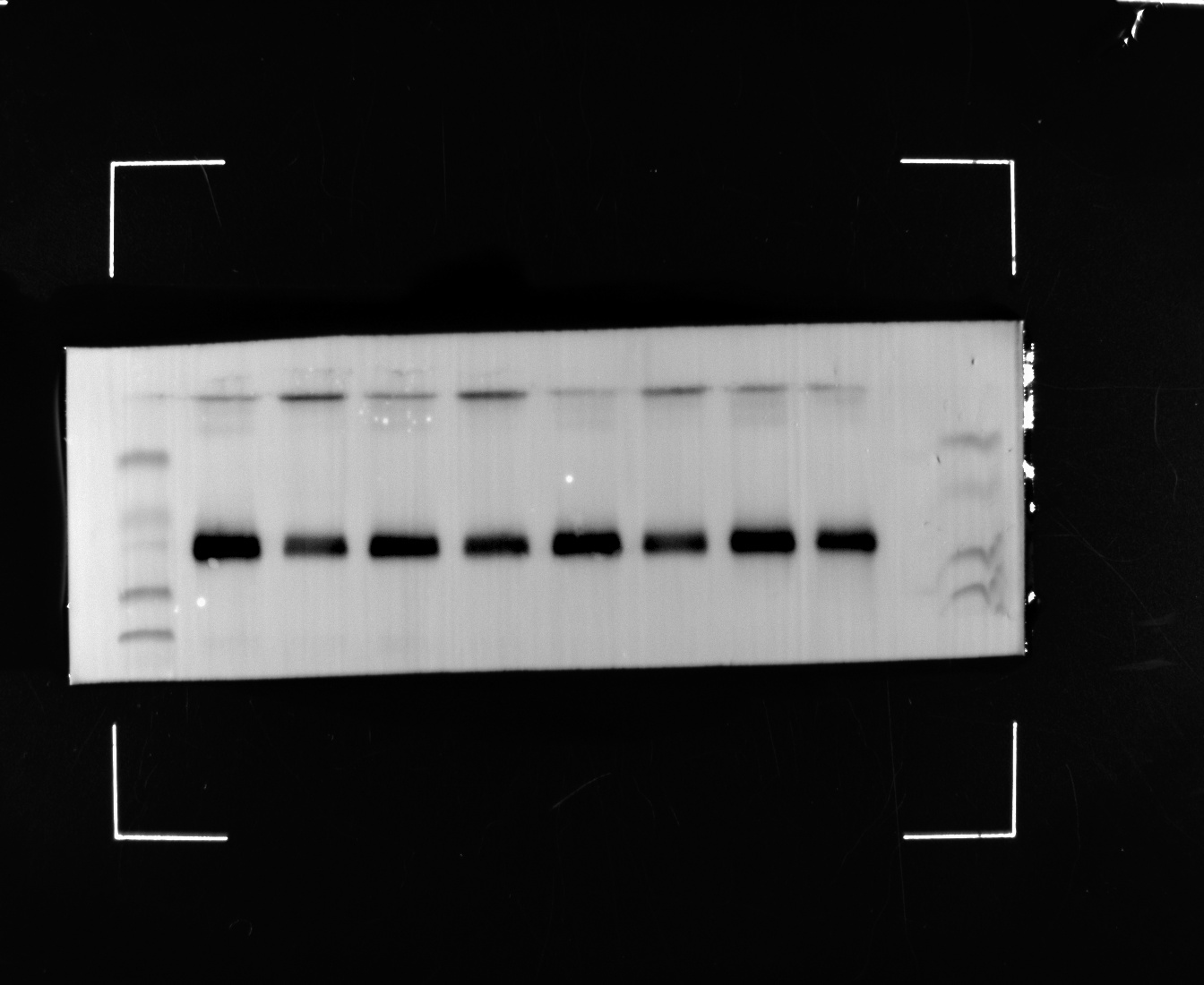

Supplement: Supplementary file 15 [file Image11.jpeg]

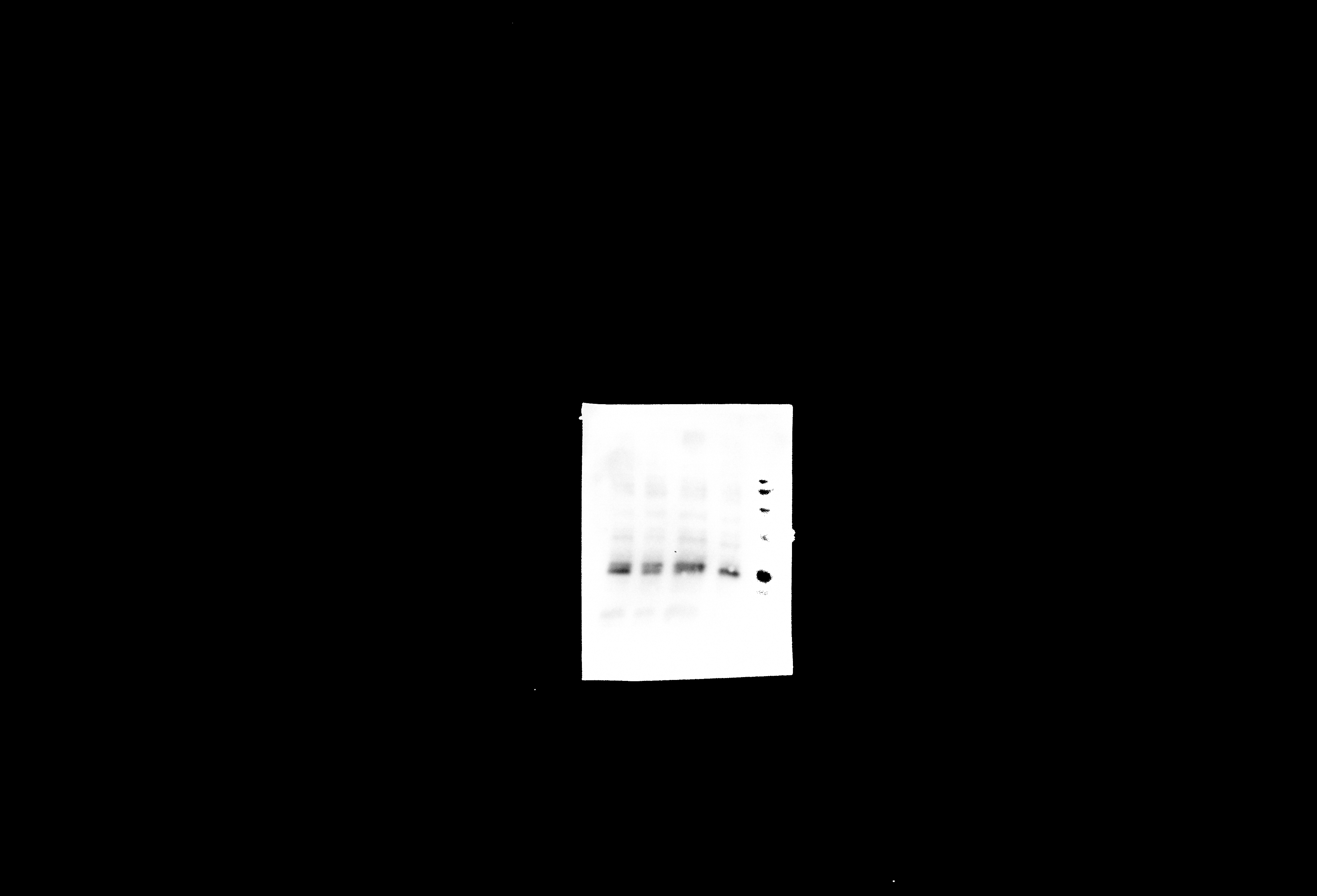

Supplement: Supplementary file 16 [file Image12.png]

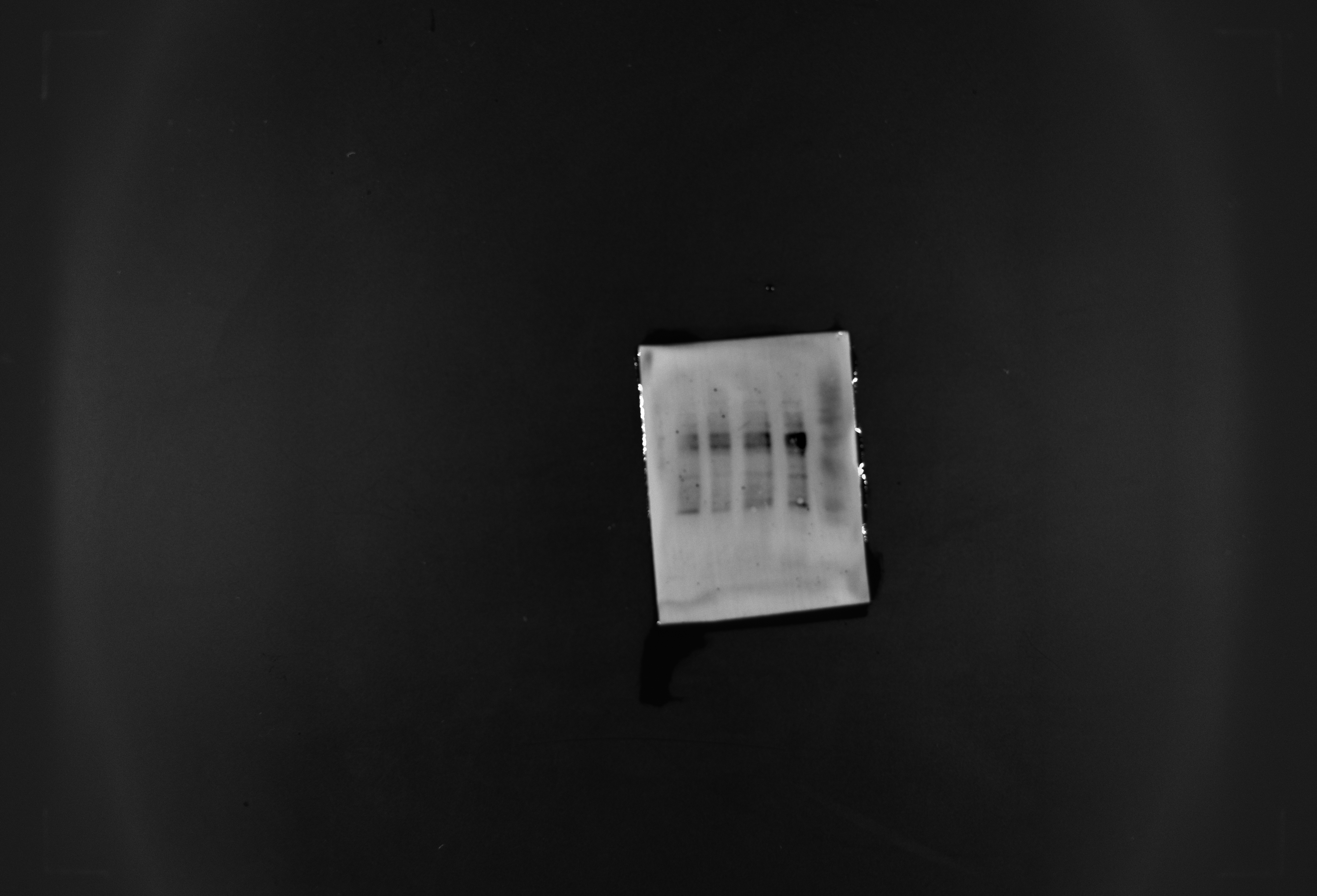

Supplement: Supplementary file 17 [file Image13.png]

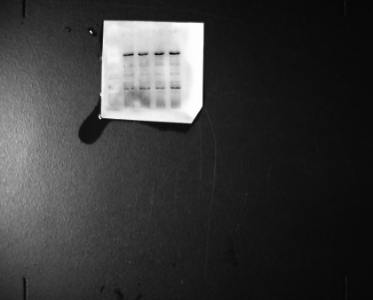

Supplement: Supplementary file 18 [file Image14.tif]
