# Supplementary material for: Administration of 2-deoxy-D-glucose induces pyroptosis in murine breast cancer cells via cAMP/PKA/HK2 to impair tumor survival
Source: Front Immunol. 2025 Dec 3;16:1724476. doi: 10.3389/fimmu.2025.1724476 (PMC12708318; doi:10.3389/fimmu.2025.1724476)
Supplement: Supplementary file 19 [file Table1.docx]

Figure 3. 2-DG induces pyroptosis in EMT6 and 4T1 cells.

Caspase-1（EMT6）：From left to right, the groups are: control group (CON), 2-Deoxy-D-glucose（2-DG）.


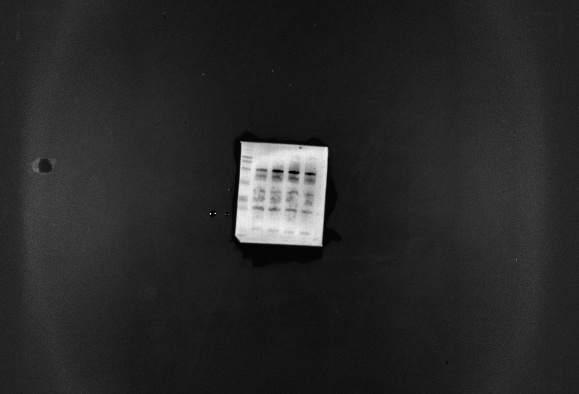

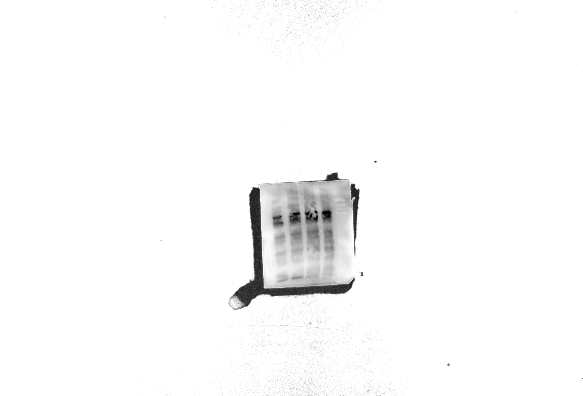


Caspase-3（EMT6）：From left to right, the groups are: control group (CON), 2-Deoxy-D-glucose（2-DG）.


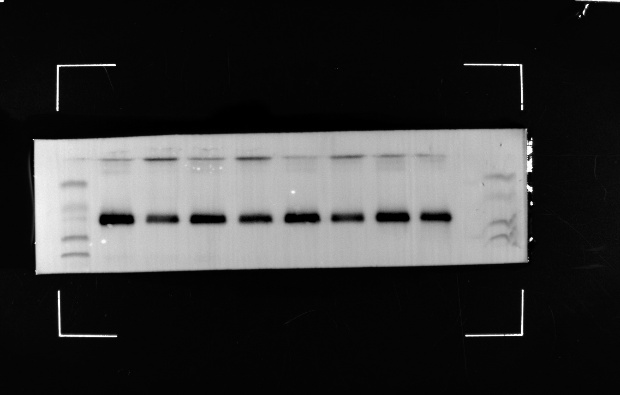


Caspase-8（EMT6）From left to right, the groups are: control group (CON), 2-Deoxy-D-glucose（2-DG）.


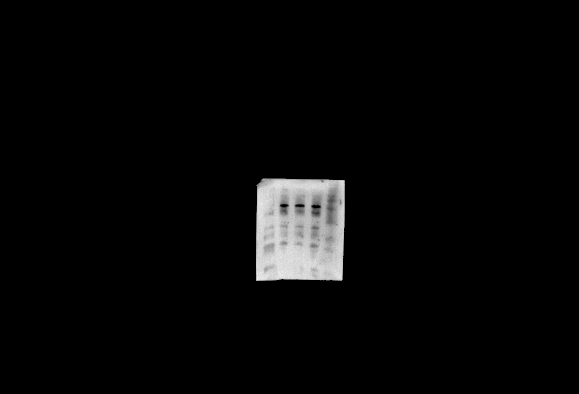

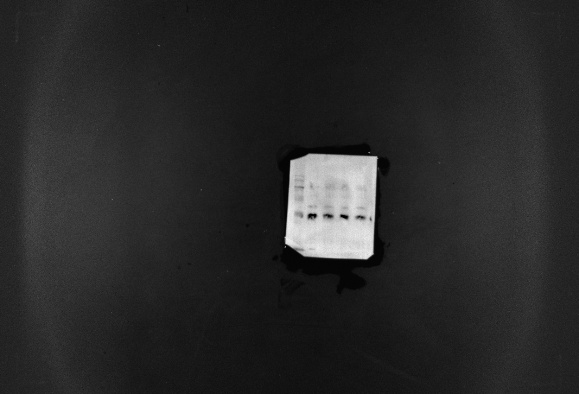


GSDMD（EMT6）：From left to right, the groups are: control group (CON), 2-Deoxy-D-glucose（2-DG）.


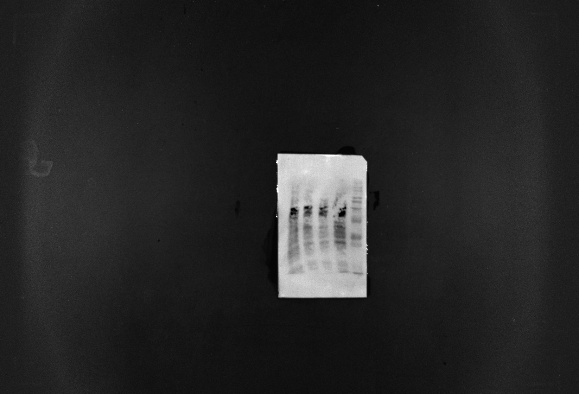

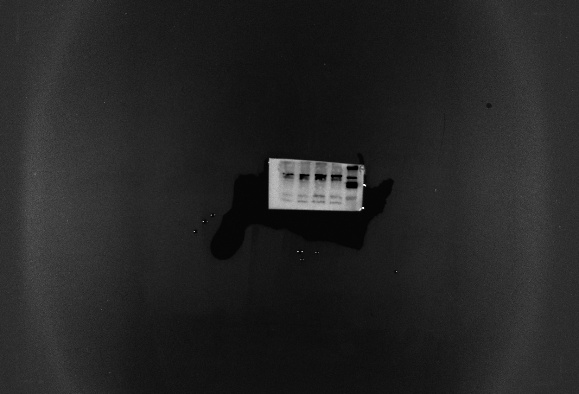


GSDME（EMT6）：From left to right, the groups are: control group (CON), 2-Deoxy-D-glucose（2-DG）.


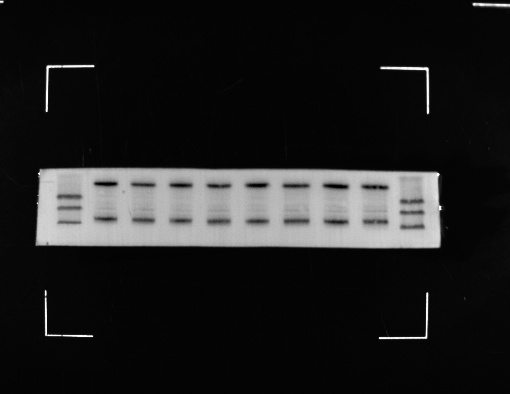

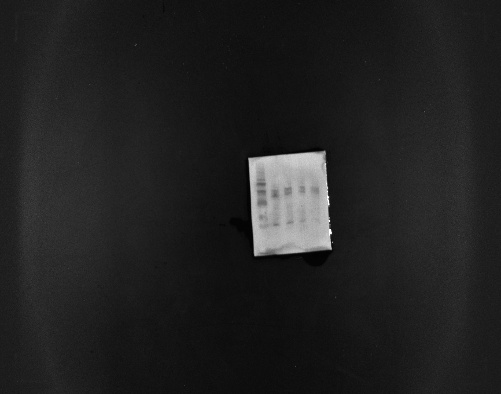


GSDMC（EMT6）：From left to right, the groups are: control group (CON), 2-Deoxy-D-glucose（2-DG）.


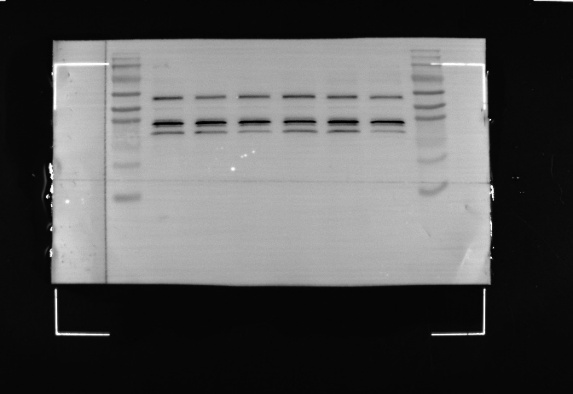


Caspase-1（4T1）：From left to right, the groups are: control group (CON), 2-Deoxy-D-glucose（2-DG）.


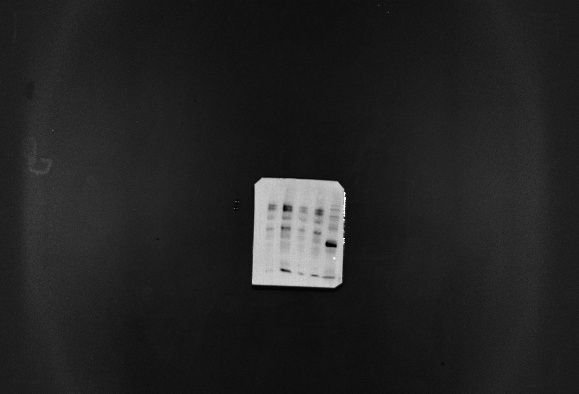

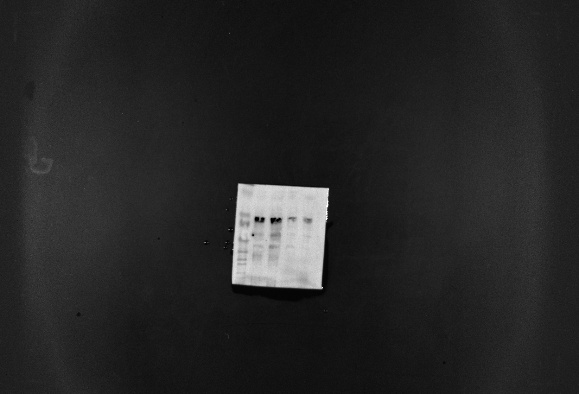


Caspase-8（4T1）: From left to right, the groups are: control group (CON), 2-Deoxy-D-glucose（2-DG）.


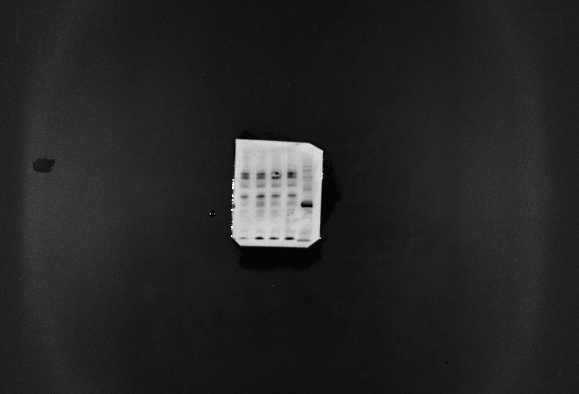

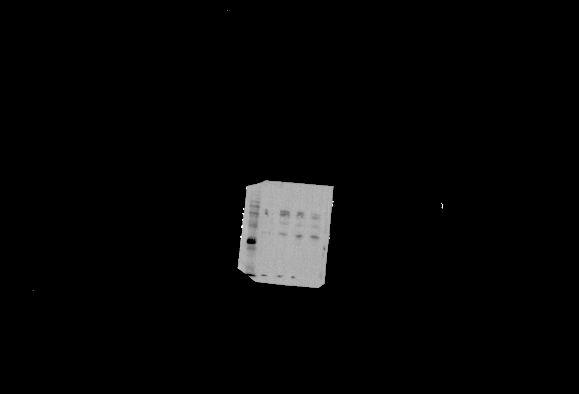


Caspase-3（4T1）：From left to right, the groups are: control group (CON), 2-Deoxy-D-glucose（2-DG）.


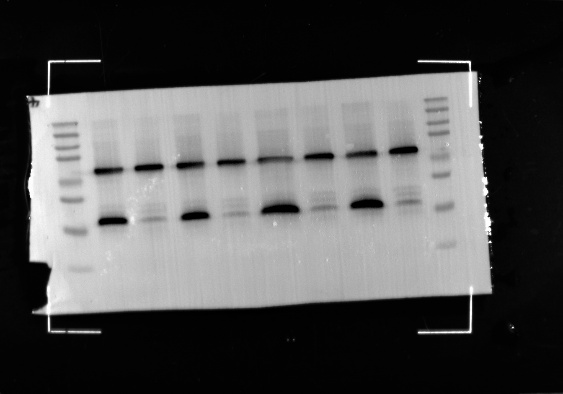


GSDMD（4T1）：From left to right, the groups are: control group (CON), 2-Deoxy-D-glucose（2-DG）.


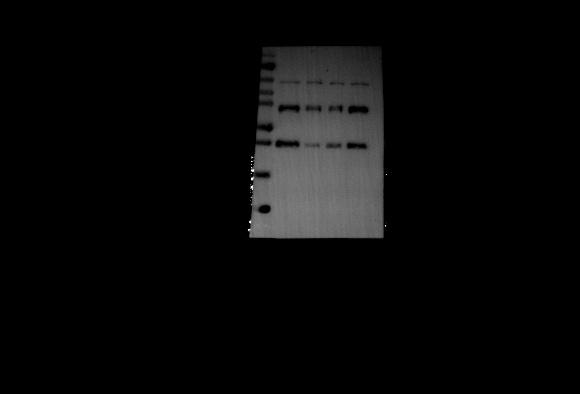

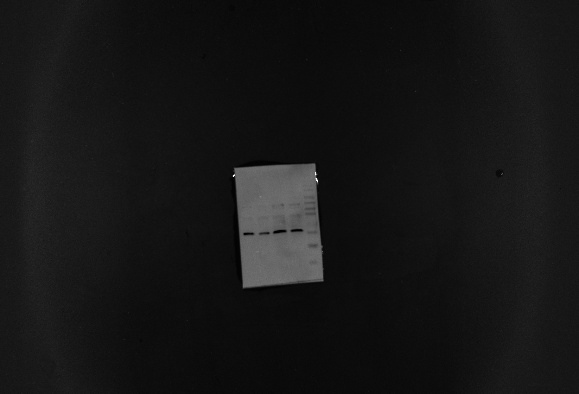


GSDMC（4T1）：From left to right, the groups are: control group (CON), 2-Deoxy-D-glucose（2-DG）.


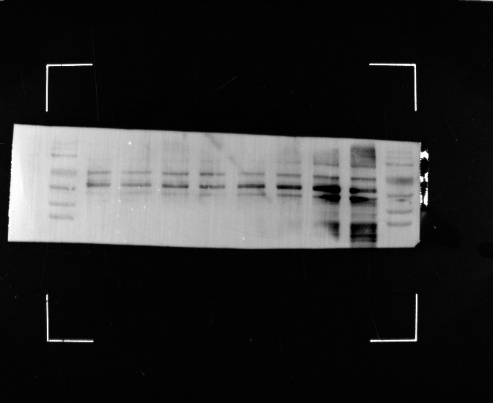


GSDME（4T1）：From left to right, the groups are: control group (CON), 2-Deoxy-D-glucose（2-DG）.


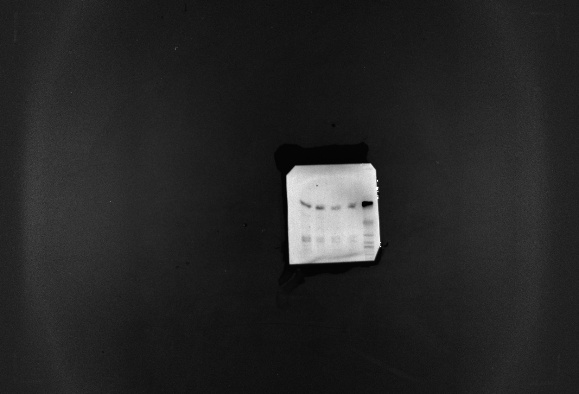

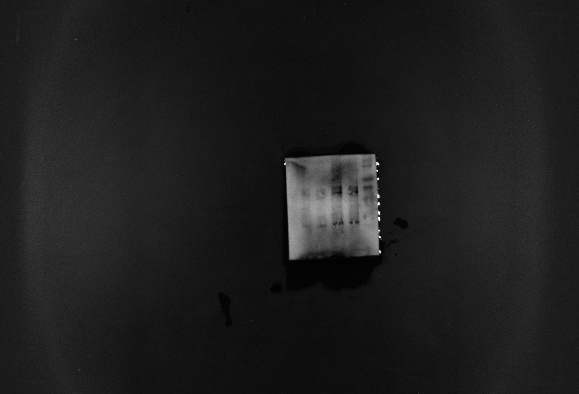


Figure 4. GSDME is the executor of 2-DG-induced pyroptosis.

Caspase-3（EMT6）：From left to right, the groups are: control group (CON), 2-Deoxy-D-glucose（2-DG）. (Both Figure 3 and this figure demonstrate the same result, so the original image is one.)


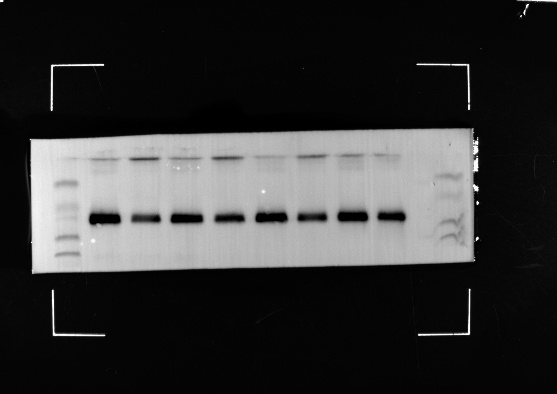


GSDME（EMT6）：From left to right, the groups are: control group (CON), 2-Deoxy-D-glucose（2-DG）. (Both Figure 3 and this figure demonstrate the same result, so the original image is one.)


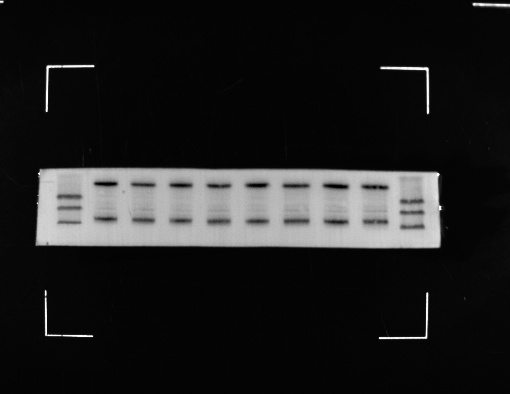

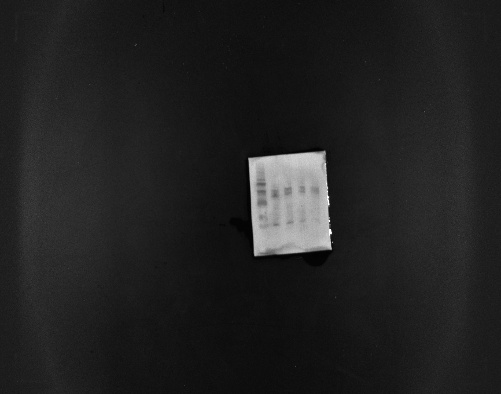


Caspase-3（4T1）：From left to right, the groups are: control group (CON), 2-Deoxy-D-glucose（2-DG）. (Both Figure 3 and this figure demonstrate the same result, so the original image is one.)


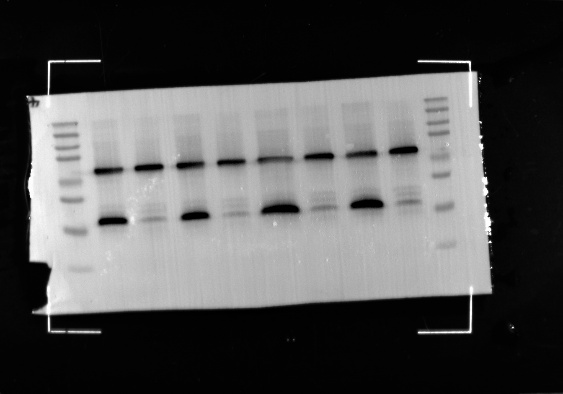


GSDME（4T1）：From left to right, the groups are: control group (CON), 2-Deoxy-D-glucose（2-DG）. (Both Figure 3 and this figure demonstrate the same result, so the original image is one.)


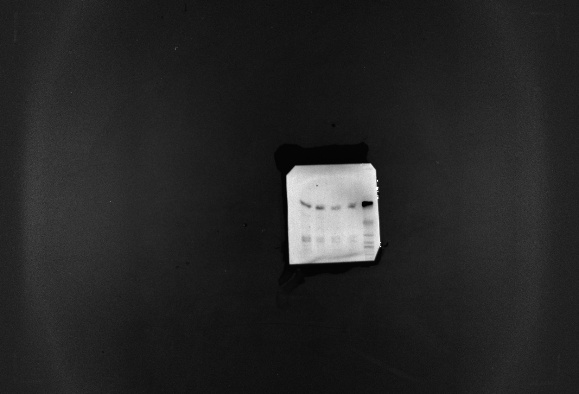

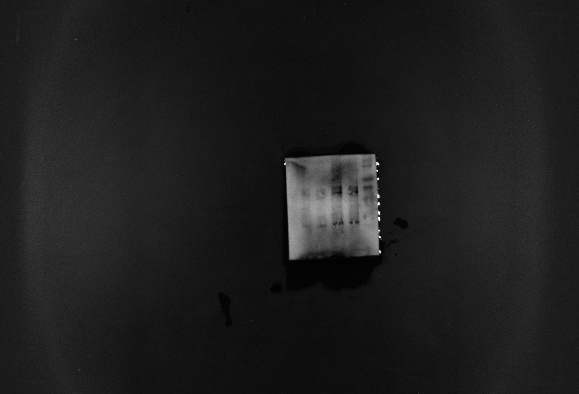


si GSDME（EMT6）：From left to right, the groups are: control group (CON), negative control group (NC) and GSDME - transfected group (siGSDME).


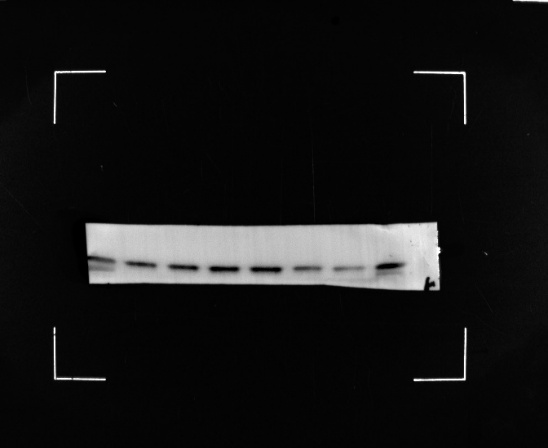

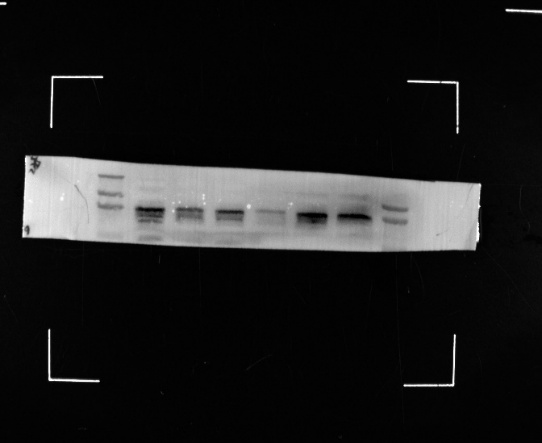


si GSDME（4T1）：From left to right, the groups are: control group (CON), negative control group (NC) and GSDME - transfected group (siGSDME).


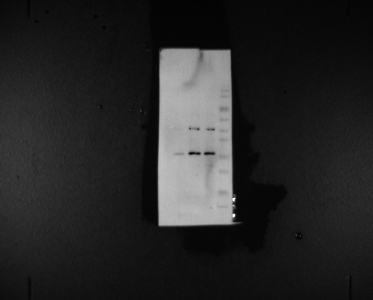

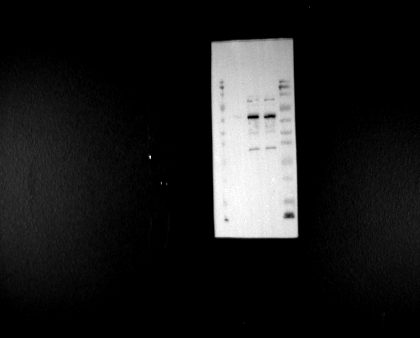


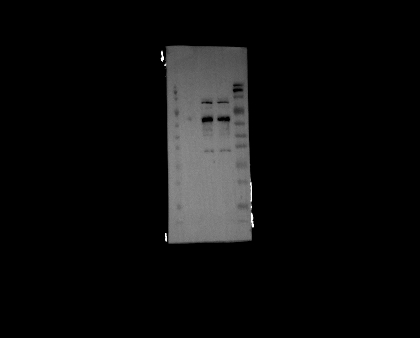


Figure 5. Caspase-3 mediates 2-DG-induced GSDME activation as an upstream factor.

GSDME（EMT6）：From left to right, the groups are: Control group (CON), 2-Deoxy-D-glucose（2-DG）, pyroptosis inhibitor group (Z-VAD-FMK), pyroptosis inhibitor + Pterostilbene group (2-DG+Z-VAD-FMK)


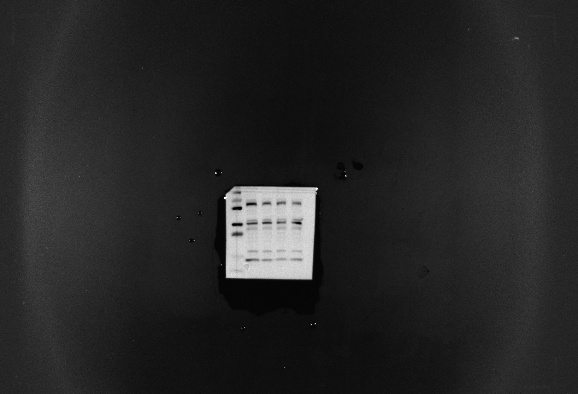

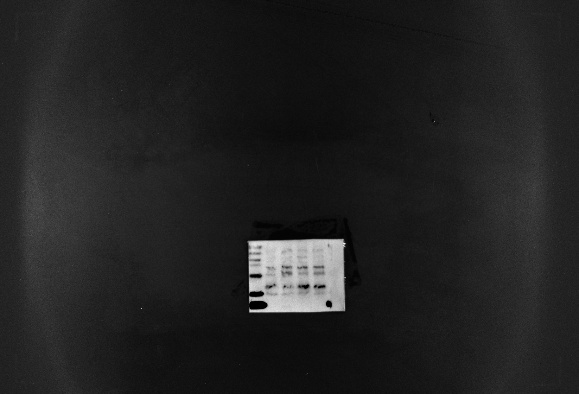


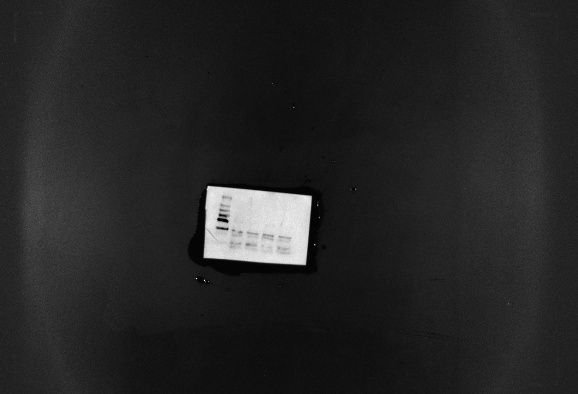


GSDME（4T1）：From left to right, the groups are: Control group (CON), 2-Deoxy-D-glucose（2-DG）, pyroptosis inhibitor group (Z-VAD-FMK), pyroptosis inhibitor + Pterostilbene group (2-DG+Z-VAD-FMK)


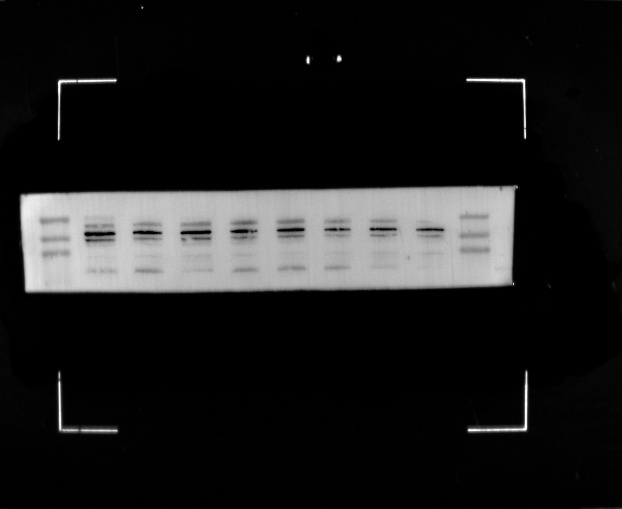

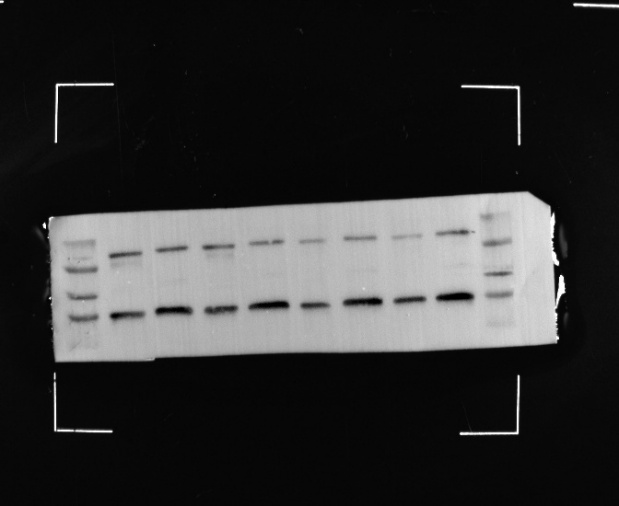


GSDME（EMT6）：From left to right, the groups are: Control group (CON), 2-Deoxy-D-glucose（2-DG）, pyroptosis inhibitor group (Z-DEVD-FMK), pyroptosis inhibitor + Pterostilbene group (2-DG+Z-DEVE-FMK)


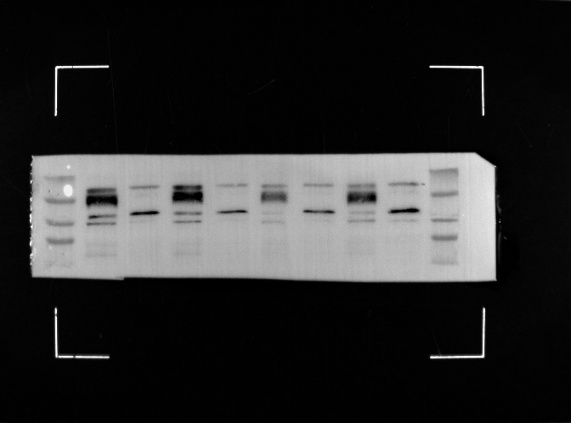

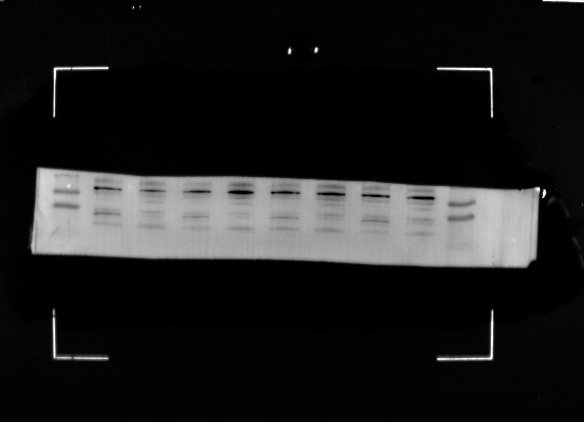


GSDME（4T1）：From left to right, the groups are: Control group (CON), 2-Deoxy-D-glucose（2-DG）, pyroptosis inhibitor group (Z-DEVD-FMK), pyroptosis inhibitor + Pterostilbene group (2-DG+Z-DEVE-FMK)


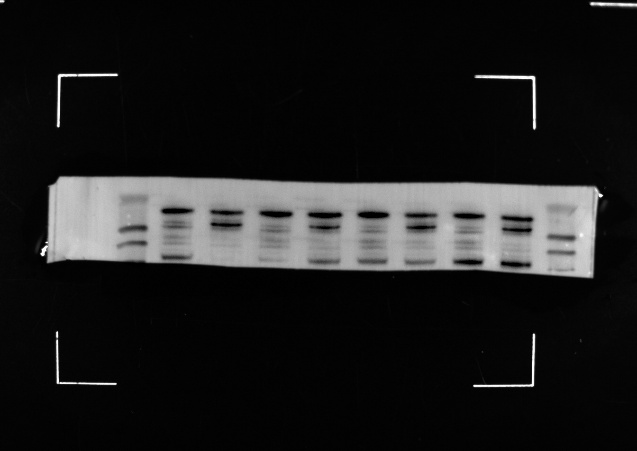

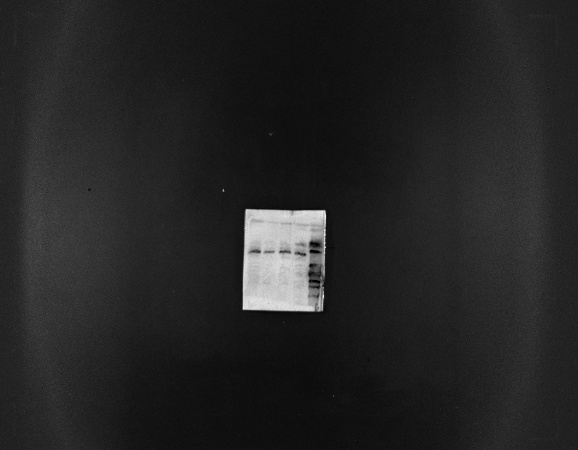


Caspase-3（EMT6）：From left to right, the groups are: Control group (CON), 2-Deoxy-D-glucose（2-DG）, Caspase-3 inhibitor + Pterostilbene group (2-DG+Z-DEVE-FMK)


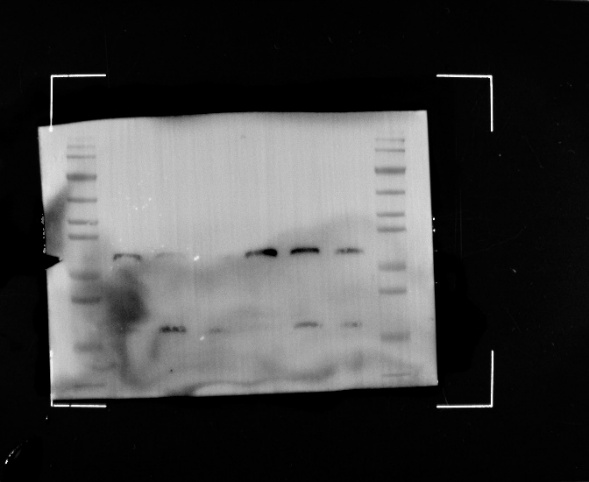

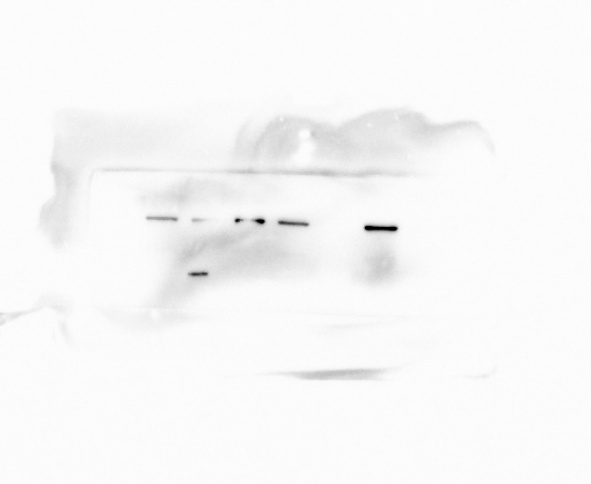


Caspase-3（4T1）：From left to right, the groups are: Control group (CON), 2-Deoxy-D-glucose（2-DG）, Caspase-3 inhibitor + Pterostilbene group (2-DG+Z-DEVE-FMK)


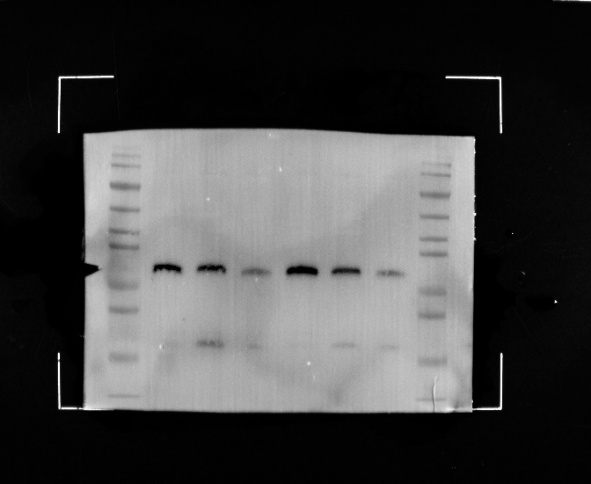

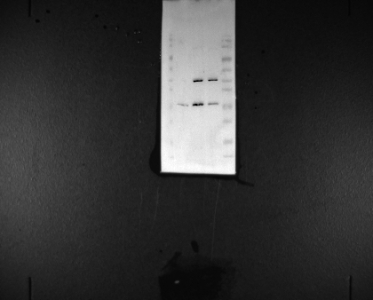


Figure 6. 2-DG induces pyroptosis in EMT6 and 4T1 cells by activating the cAMP/PKA pathway to inhibit HK2.

cAMP（EMT6）：From left to right, the groups are: Control group (CON), 2-Deoxy-D-glucose（2-DG）.


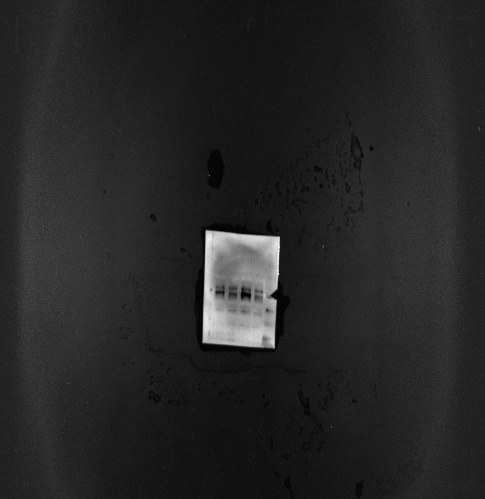

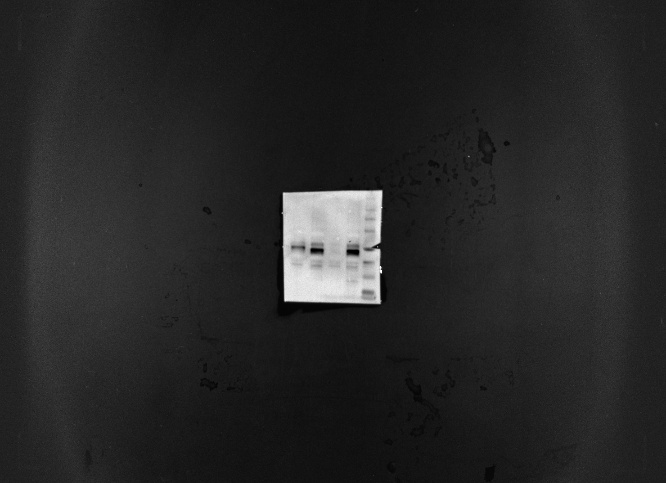


p-cAMP（EMT6）：From left to right, the groups are: Control group (CON), 2-Deoxy-D-glucose（2-DG）.


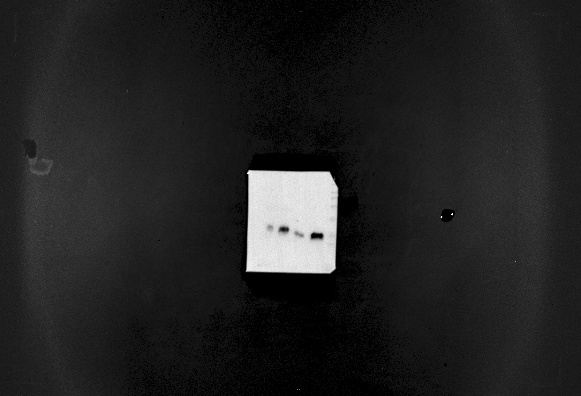

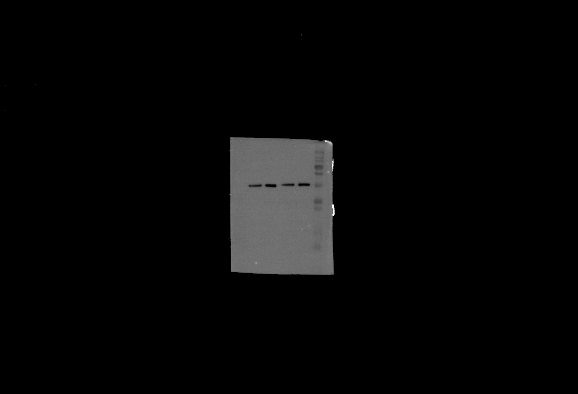


cAMP（4T1）：From left to right, the groups are: Control group (CON), 2-Deoxy-D-glucose（2-DG）.


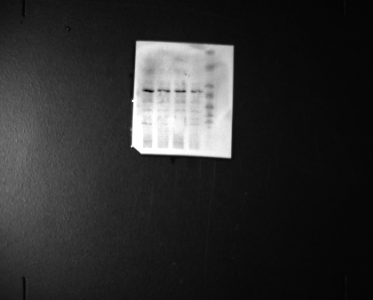

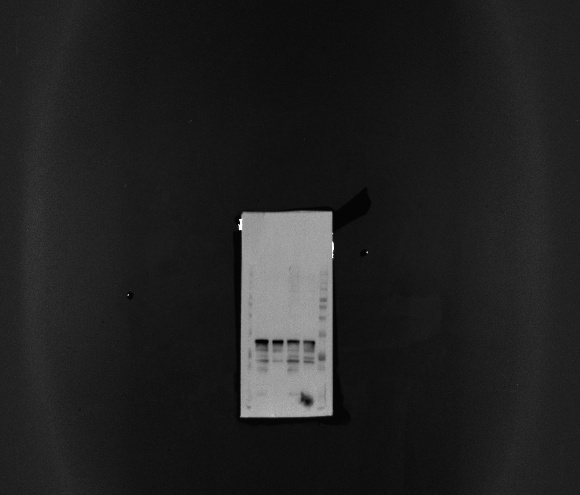


cAMP（EMT6）：From left to right, the groups are: Control group (CON), 2-Deoxy-D-glucose（2-DG）.


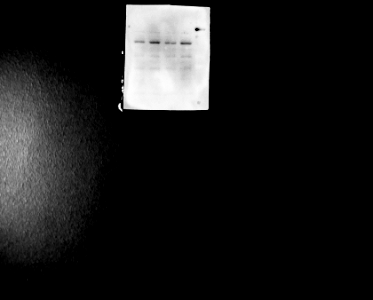

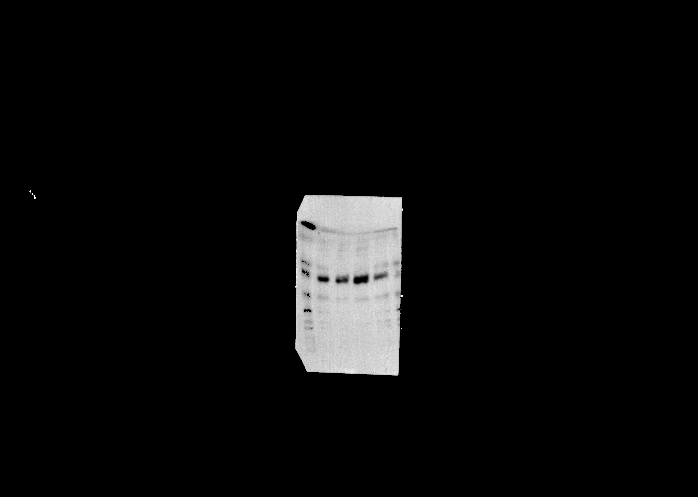


PKA（EMT6）：From left to right, the groups are: Control group (CON), 2-Deoxy-D-glucose（2-DG）.


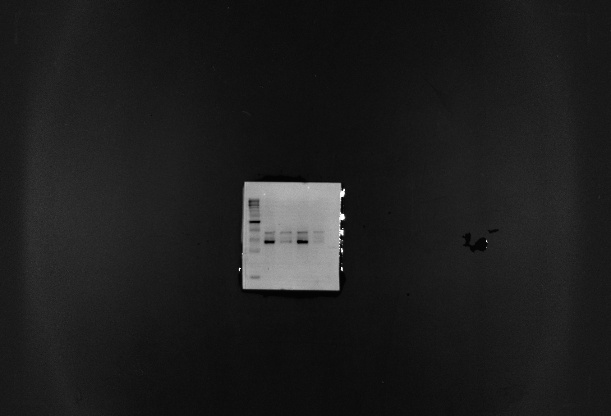

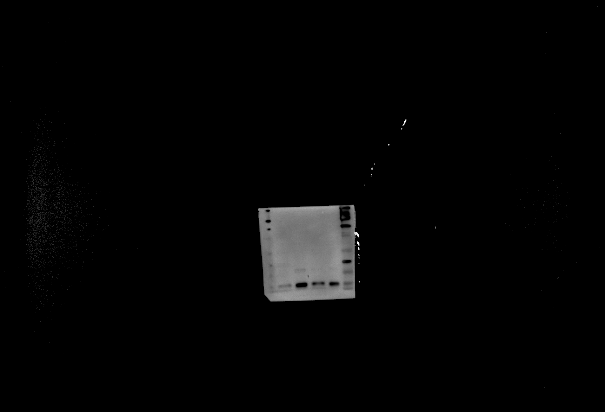


p-PKA（EMT6）：From left to right, the groups are: Control group (CON), 2-Deoxy-D-glucose（2-DG）.


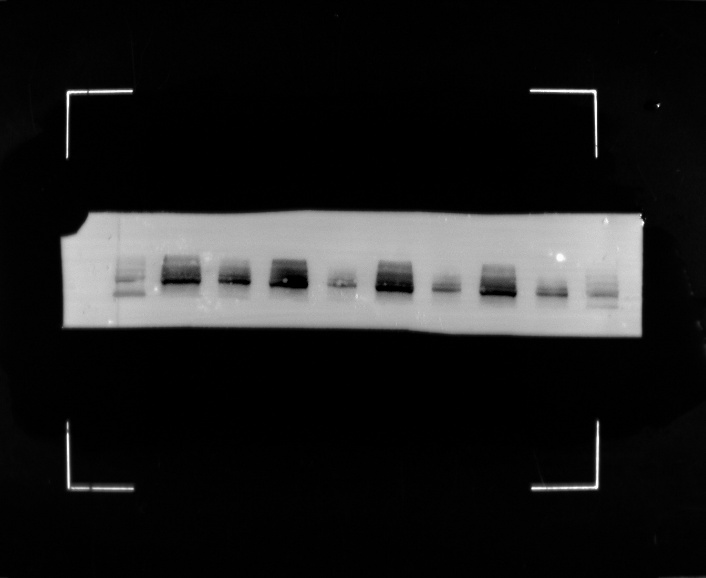


PKA（4T1）：From left to right, the groups are: Control group (CON), 2-Deoxy-D-glucose（2-DG）.


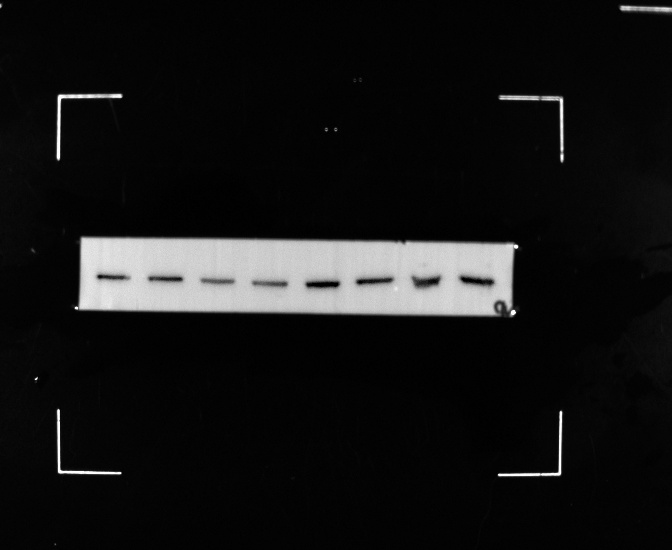


p-PKA（4T1）：From left to right, the groups are: Control group (CON), 2-Deoxy-D-glucose（2-DG）.


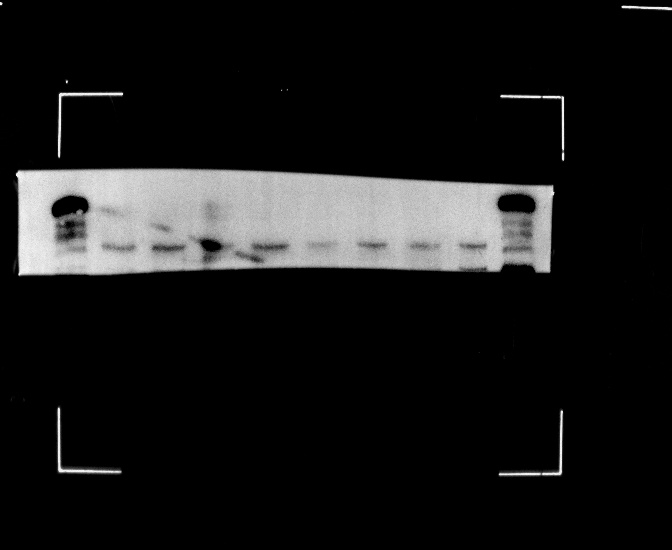


HK2（EMT6）：From left to right, the groups are: Control group (CON), 2-Deoxy-D-glucose（2-DG）.


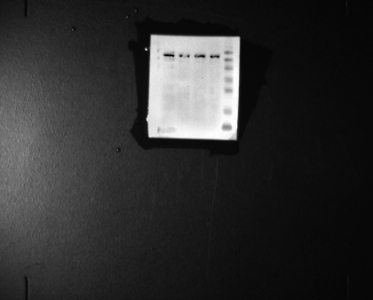

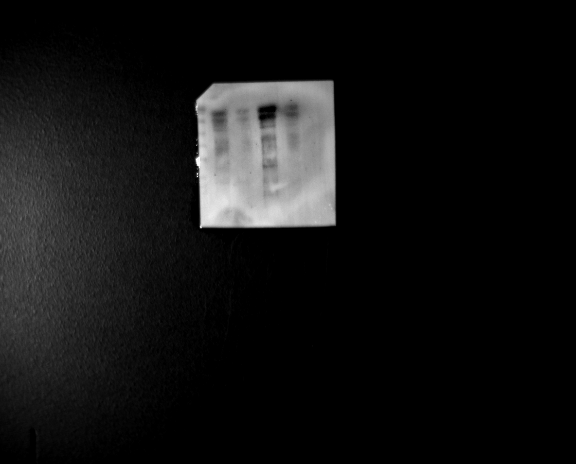


HK2（4T1）：From left to right, the groups are: Control group (CON), 2-Deoxy-D-glucose（2-DG）.


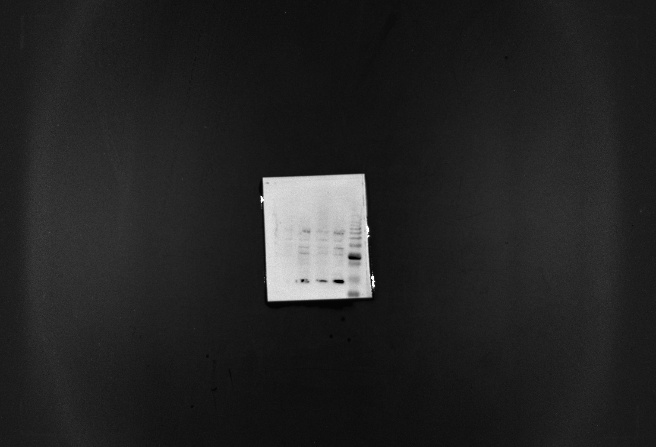

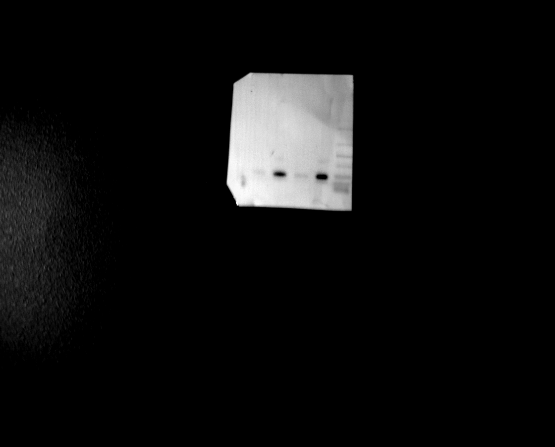


HK2（EMT6）：From left to right, the groups are: Control group (CON), 2-DG group (2-DG), PKA inhibitor group (H-89), PKA inhibitor + 2-DG group (2-DG + H-89).


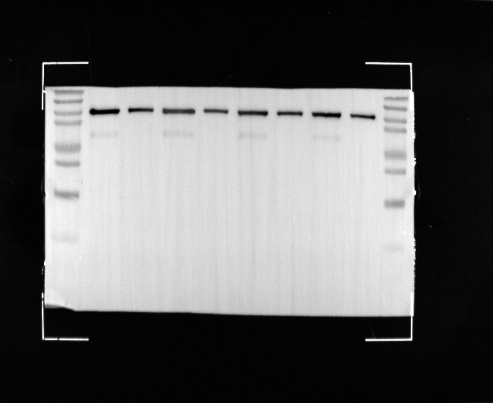

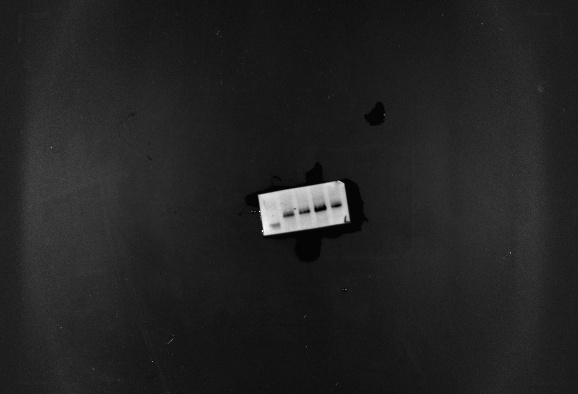


HK2（4T1）：From left to right, the groups are: Control group (CON), 2-DG group (2-DG), PKA inhibitor group (H-89), PKA inhibitor + 2-DG group (2-DG + H-89).


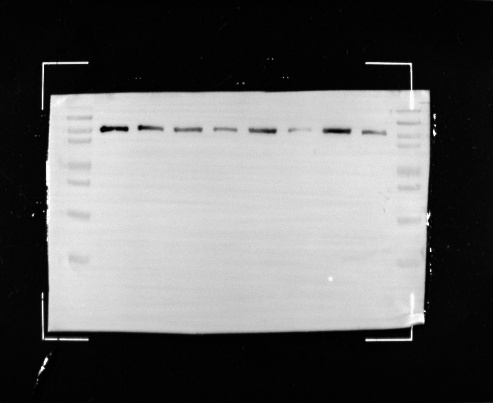

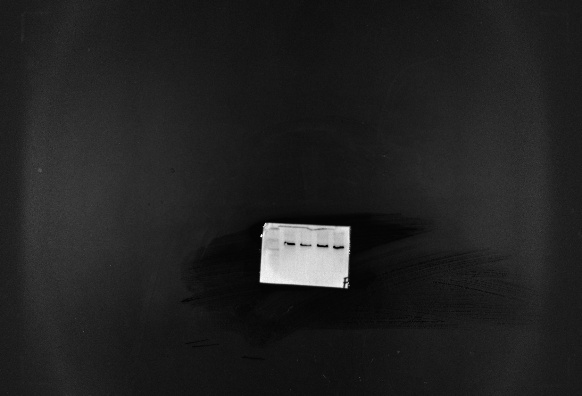


Caspase-3（EMT6）：From left to right, the groups are: Control group (CON), 2-DG group (2-DG), HK2 activator group (c-Src), HK2 activator + 2-DG group (2-DG + c-Src)


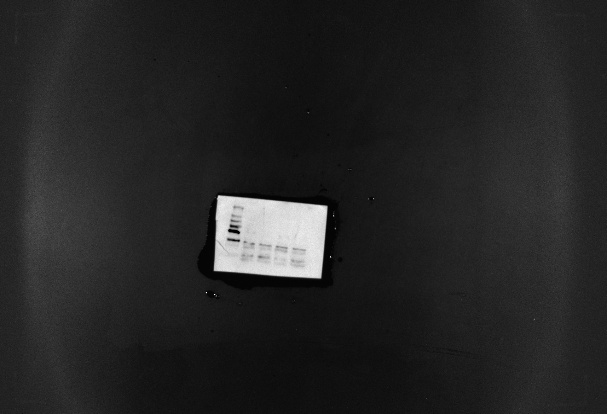

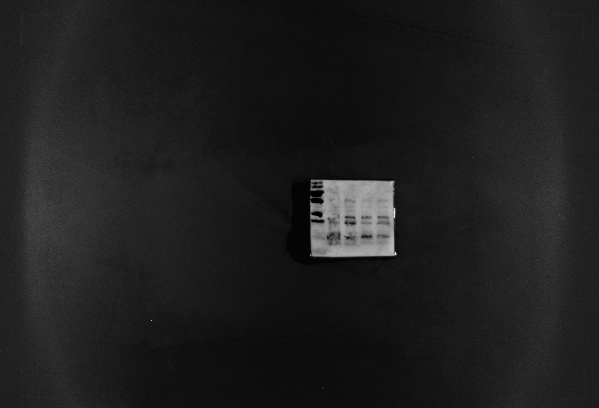


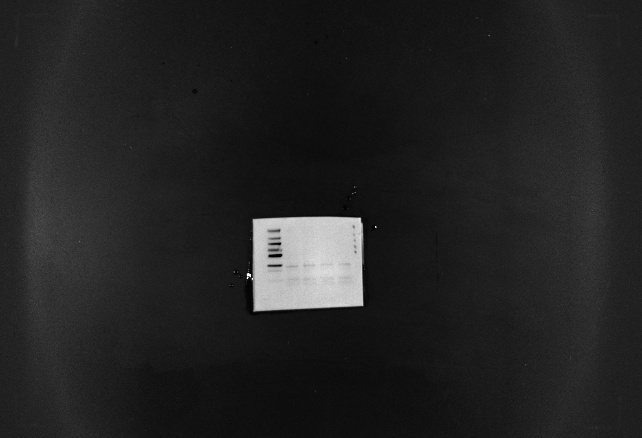


Caspase-3（4T1）：From left to right, the groups are: Control group (CON), 2-DG group (2-DG), HK2 activator group (c-Src), HK2 activator + 2-DG group (2-DG + c-Src)


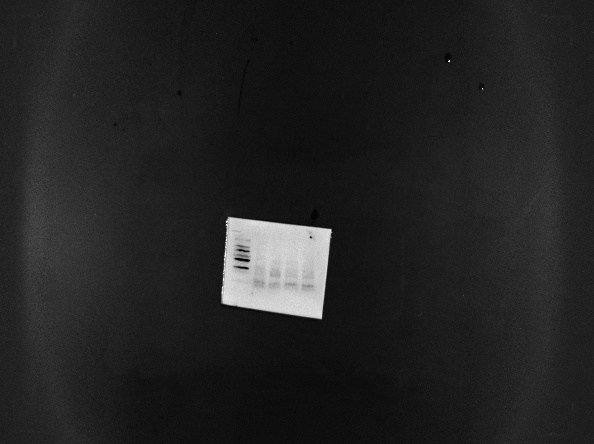

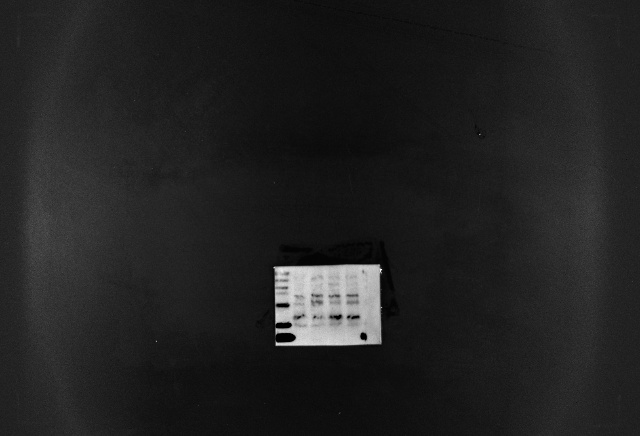


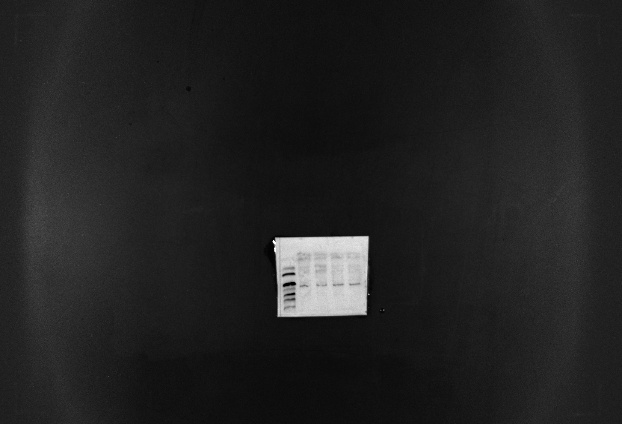


GSDME（EMT6）：From left to right, the groups are: Control group (CON), 2-DG group (2-DG), HK2 activator group (c-Src), HK2 activator + 2-DG group (2-DG + c-Src)


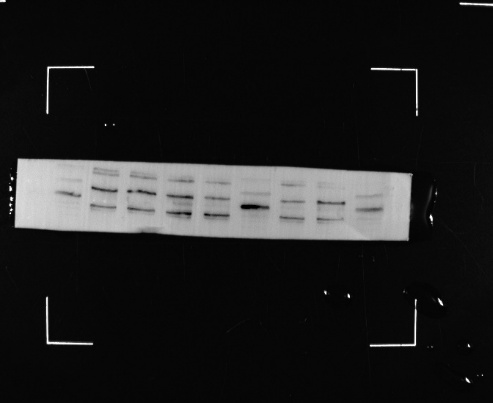

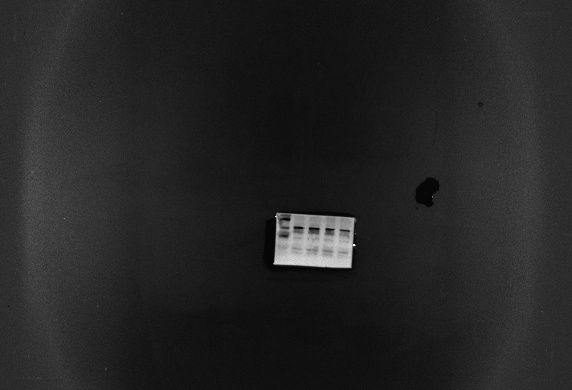


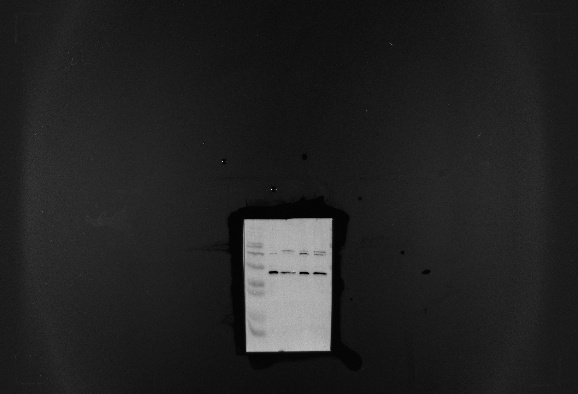


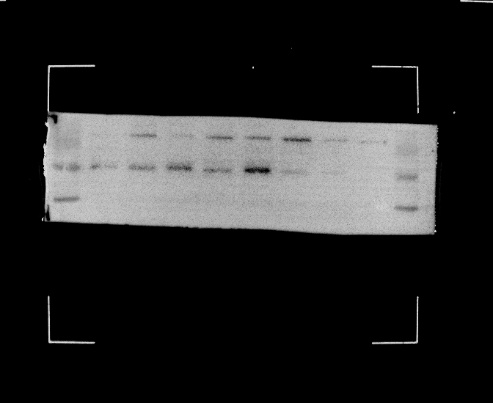


GSDME（4T1）：From left to right, the groups are: Control group (CON), 2-DG group (2-DG), HK2 activator group (c-Src), HK2 activator + 2-DG group (2-DG + c-Src)


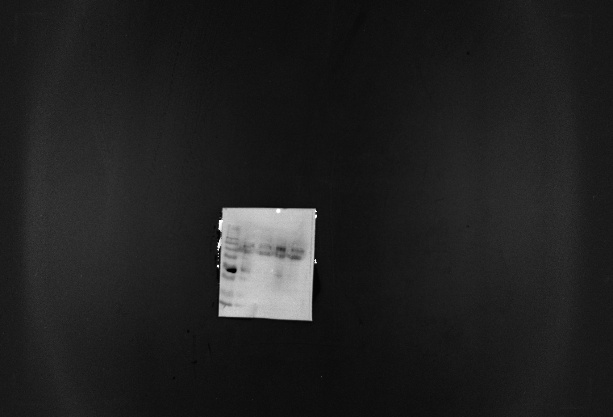

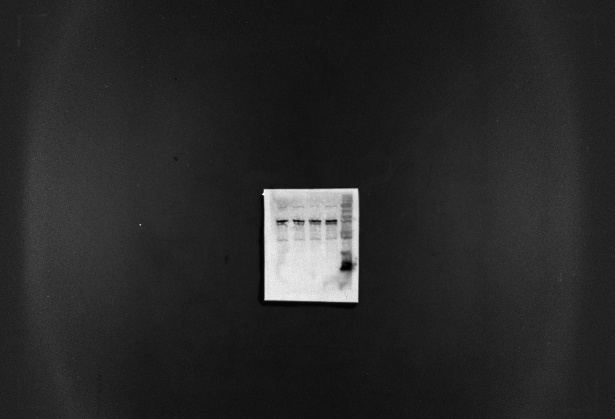


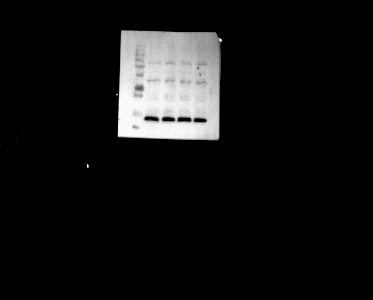


Figure 7. Antitumor effects of 2-DG in vivo.

CREB：From left to right, the groups are: Model group (MOD), 2-DG low-dose group (2-DG-L), 2-DG high-dose group (2-DG-H)


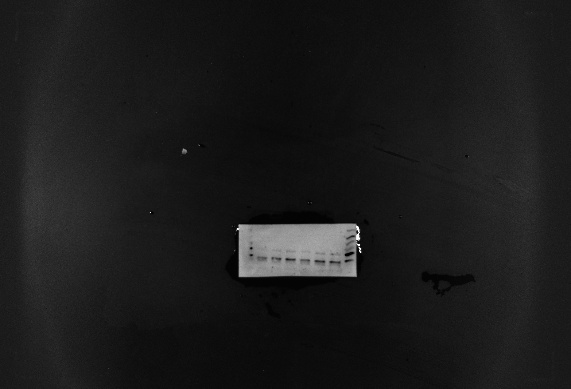

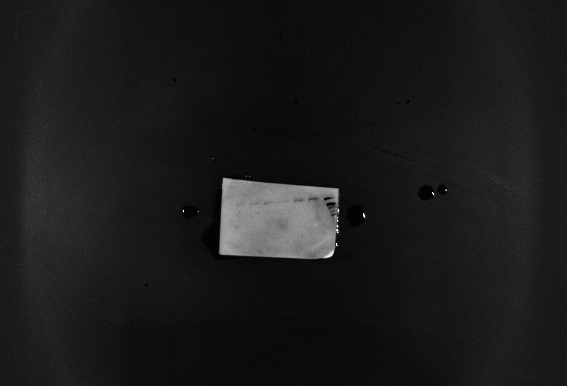


p-CREB：From left to right, the groups are: Model group (MOD), 2-DG low-dose group (2-DG-L), 2-DG high-dose group (2-DG-H)


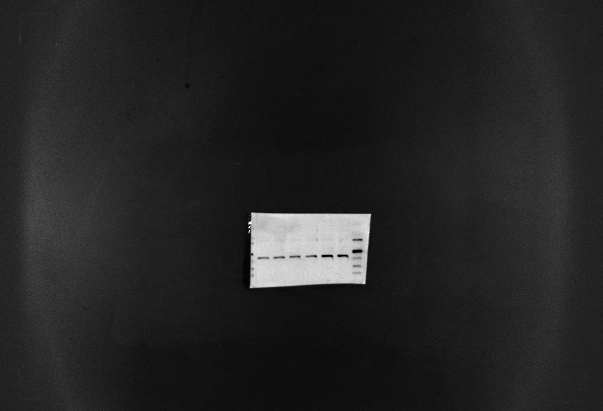

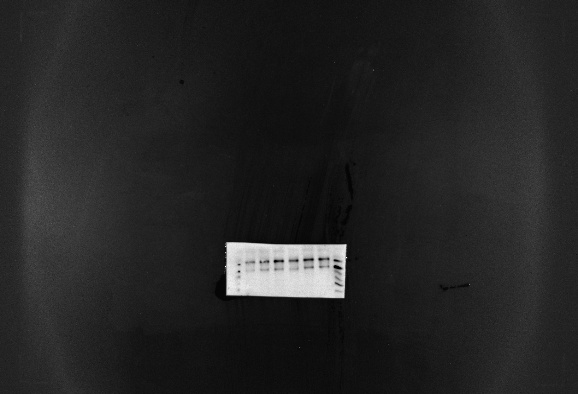


PKA：From left to right, the groups are: Model group (MOD), 2-DG low-dose group (2-DG-L), 2-DG high-dose group (2-DG-H)


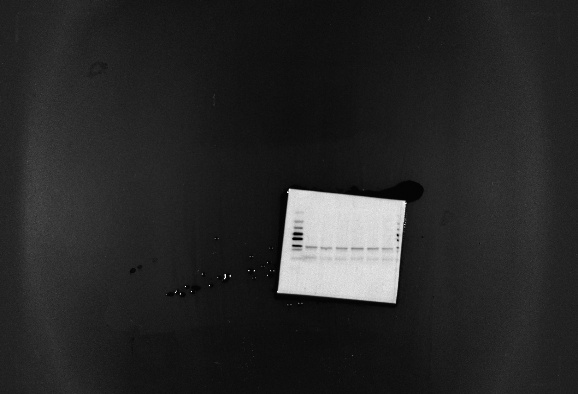

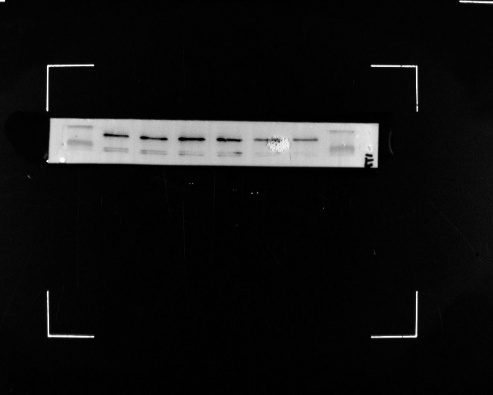


p-PKA：From left to right, the groups are: Model group (MOD), 2-DG low-dose group (2-DG-L), 2-DG high-dose group (2-DG-H)


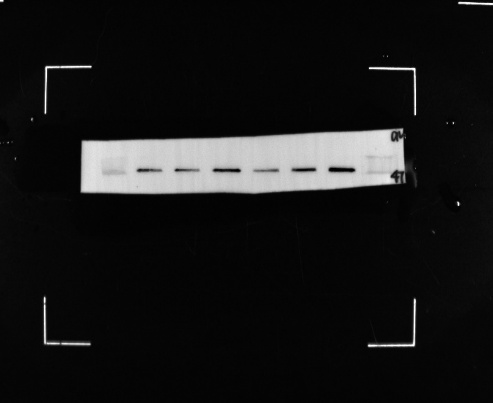
**
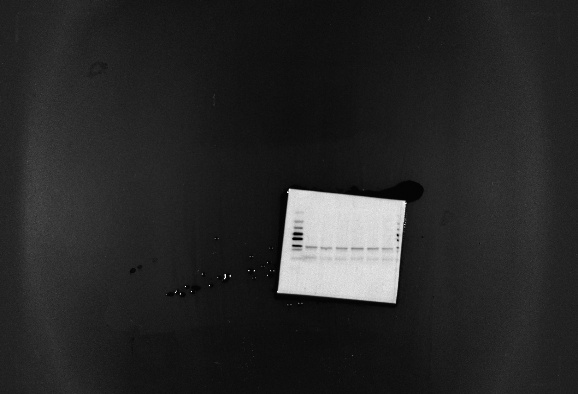
**
